# Kinetic studies on strand displacement in *de novo* designed parallel heterodimeric coiled coils†
†Electronic supplementary information (ESI) available. See DOI: 10.1039/c7sc05342h


**DOI:** 10.1039/c7sc05342h

**Published:** 2018-04-17

**Authors:** Mike C. Groth, W. Mathis Rink, Nils F. Meyer, Franziska Thomas

**Affiliations:** a Georg-August-Universität Göttingen , Institute of Organic and Biomolecular Chemistry , Tammannstraße 2 , 37077 Göttingen , Germany . Email: fthomas@gwdg.de; b Center for Biostructural Imaging of Neurodegeneration , Von-Siebold-Straße 3a , 37075 Göttingen , Germany

## Abstract

Among the protein folding motifs, which are accessible by *de novo* design, the parallel heterodimeric coiled coil is most frequently used in bioinspired applications and chemical biology in general. This is due to the straightforward sequence-to-structure relationships, which it has in common with all coiled-coil motifs, and the heterospecificity, which allows control of association. Whereas much focus was laid on designing orthogonal coiled coils, systematic studies on controlling association, for instance by strand displacement, are rare. As a contribution to the design of dynamic coiled-coil-based systems, we studied the strand-displacement mechanism in obligate heterodimeric coiled coils to investigate the suitability of the dissociation constants (*K*_D_) as parameters for the prediction of the outcome of strand-displacement reactions. We use two sets of heterodimeric coiled coils, the previously reported N-A_*x*_B_*y*_ and the newly characterized C-A_*x*_B_*y*_. Both comprise *K*_D_ values in the μM to sub-nM regime. Strand displacement is explored by CD titration and a FRET-based kinetic assay and is proved to be an equilibrium reaction with half-lifes from a few seconds up to minutes. We could fit the displacement data by a competitive binding model, giving rate constants and overall affinities of the underlying association and dissociation reactions. The overall affinities correlate well with the ratios of *K*_D_ values determined by CD-thermal denaturation experiments and, hence, support the dissociative mechanism of strand displacement in heterodimeric coiled coils. From the results of more than 100 different displacement reactions we are able to classify three categories of overall affinities, which allow for easy prediction of the equilibrium of strand displacement in two competing heterodimeric coiled coils.

## Abstract 2

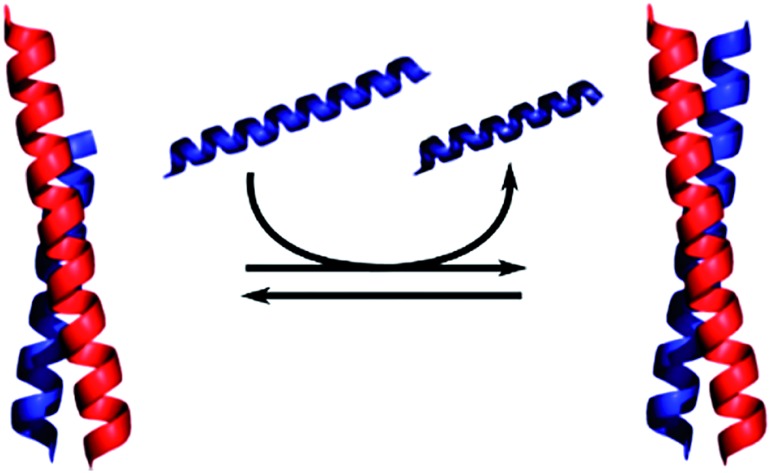
Strand displacement in heterodimeric coiled coils follows a competitive binding mechanism and can be predicted by the ratio of *K*_D_ values.

## Introduction

*De novo* designed protein folding motifs have been increasingly applied in the design of bioinspired systems or biomaterials from the bottom-up.^1^,^2^ Among the protein folds the coiled-coil motif is currently best understood and mostly used in the above-mentioned fields.^3^,^4^ The straightforward sequence-to-structure relationships, mainly the repetitive heptad-repeat sequence pattern of hydrophobic (h) and polar (p) residues – in short hpphppp, mostly denoted as *abcdefg* register – significantly facilitates peptide design.^5^ Currently, reliable design rules are available for coiled-coil dimers to tetramers and higher order oligomers.^6^,^7^

Among the available coiled-coil assemblies the heterodimeric coiled coil has gained significant scientific attention. The heterogeneity of the two coil strands adds a control element to the assembly, which makes it an ideal simplified model for protein–protein interactions.^4^ Therefore, the heterodimeric coiled-coil motif has found widespread use in the design of biomimetic systems,^8^ such as dimerization domain in an engineered EGF receptor^9^ or recognition motif for SNARE-mediated vesicle fusion.^10^,^11^ Furthermore, applications as affinity tags in protein labelling^12^,^13^ or protein purification have been reported.^14^,^15^ Some peptide materials including hydrogels, cages or nanotubes are also based on the heterodimeric coiled-coil interaction as a central design element.^16^–^23^ Other applications include reaction mediation in *e.g.* acyl-transfer reactions,^24^–^27^ and C–H activation.^28^

The simplest rules to design a parallel heterodimeric coiled coil include an isoleucine–leucine core to direct dimerization,^29^ and the introduction of positively charged lysines (Lys) to the *e* and *g* positions of one coil strand and negatively charged glutamic acid residues (Glu) to the *e* and *g* positions of the other coil strand to induce heterospecificity.^30^–^34^ Often, asparagine residues (Asn) at the *a* positions of a central coiled-coil heptad are incorporated as an additional design feature to improve the dimer specificity and to stabilize the parallel orientation.^35^–^39^ However, precise control of association and dissociation, which also includes variation of the strength of association, is highly desirable in many applications. Therefore, efforts have been made to generate orthogonal coiled coils by modulating the charge pattern of the individual coil strands, or by engineering polar recognition motifs into the hydrophobic core.^19^,^40^–^43^ Very recently, an iterative approach to create larger sets of orthogonal heterodimeric coiled coils has been presented.^44^ In comparison, only little has been reported on simple rules to modulate the strength of coiled-coil association. This is important, as it allows engineering of dynamic systems, such as drug delivery devices.^45^ Concepts include modulation of the strengths of the salt bridges or the hydrophobic interactions by varying the lengths of the amino acid side chains at the coiled-coil interface.^46^–^48^

Recently, a set of heterodimeric coiled coils, A_*x*_B_*y*_, was presented, in which the strength of coiled-coil interaction was modulated by the lengths of the individual coil strands.^37^ The design was mainly based on the Hodges EK peptides;^31^ however, the introduction of Asn at a central *a* position was vital for enabling the formation of distinct coiled-coil assemblies, in which the individual strands varied in lengths of 3, 3.5 and 4 heptads, truncated from the N-terminal end. Combination resulted in a set of nine heterodimeric coiled coils with dissociation constants in the μM to sub-nM regime.

The range of *K*_D_ values within the A_*x*_B_*y*_ set of heterodimeric coiled coils should open new routes to the design of dynamic functional systems based on strand displacement. Indeed, *K*_D_ values have often been discussed as parameters to assess such displacement events in heterodimeric coiled coils.^8^,^37^,^49^ However, the mechanism has hardly been studied so far and easy models might not apply as dissociation of heterodimeric coiled coils goes along with unfolding.^30^,^37^ Until now, there is only one application reported, in which strand displacement in heterodimeric coiled coils was used to design a two-state switching system to control the interaction of proteins.^49^ Although displacement was discussed to be thermodynamically driven, no details on the mechanism or the underlying equilibria were revealed. To use strand displacement reliably in the design of functional systems, we need more thorough information. Furthermore, a correlation of strand-displacement equilibria to *K*_D_-value differences would be desirable to facilitate designs. Herein, we present an extensive study of strand displacement in A_*x*_B_*y*_ peptides, which, for clarity reasons, we dub N-A_*x*_B_*y*_, and a similar set of heterodimeric coiled coils, C-A_*x*_B_*y*_, in which the individual peptides are truncated from the C-terminal end. We aim to understand the mechanism of strand displacement in heterodimeric coiled coils by performing CD-titration experiments and a FRET-based kinetic strand-displacement assay. This leads to an easy-to-use rule of thumb to predict outcomes of strand displacements purely based on *K*_D_ values.

## Experimental

### General

Fmoc-protected amino acids were purchased from GL Biochem LTD (Shanghai, China). DIC and Oxyma Pure were obtained from Iris Biotech GmbH (Marktredwitz, Germany). The H-Rink Amide-ChemMatrix® resin was acquired from Sigma Aldrich (Taufkirchen, Germany). DMF used for peptide synthesis was supplied by Fisher Scientific (Schwerte, Germany) and was of peptide grade quality. Acetonitrile used for HPLC was supplied by Fisher Scientific (Schwerte, Germany) with HPLC grade quality. Water used for HPLC and reactions was obtained by purifying deionized water with the purification device Simplicity from Millipore. All other reagents were supplied by Sigma Aldrich (Taufkirchen, Germany), Thermo Fisher Scientific (Langenselbold, Germany), VWR International (Darmstadt, Germany) and Carl Roth (Karlsruhe, Germany). All reagents were of synthesis grade quality and were used as supplied. Unless otherwise stated, biophysical measurements were performed in phosphate buffered saline (PBS, 8.2 mM Na_2_HPO_4_, 1.8 mM K_2_HPO_4_, 137 mM NaCl, 2.7 mM KCl, pH 7.4). Peptide concentrations were determined by UV-absorbance at 280 nm (*ε*_280_(Trp) = 5690 mol^–1^ cm^–1^, *ε*_280_(Tyr) = 1280 mol^–1^ cm^–1^), and, alternatively, at 214 nm using a NanoDrop 2000 spectrophotometer from Thermo Scientific. Extinction coefficients at 214 nm were calculated according to Kuipers *et al.*^50^

### Peptide synthesis

The peptide amides were synthesized on a H-Rink Amide-ChemMatrix® resin on a 0.1 mmol scale on a Liberty Blue CEM microwave-assisted peptide synthesizer. The synthesis was conducted *via* a standard Fmoc/*t*Bu-protocol using DIC and Oxyma Pure as coupling reagents and a solution of piperidine in DMF (1 : 4 (v/v)) for Fmoc-removal. *N*-Acetylation of the peptides was carried out by equilibrating the peptide resin with 5 mL of acetic acid anhydride/pyridine (1 : 9 (v/v)) for 5 min at room temperature. Acidic cleavage from the resin was achieved by a treatment with a mixture of trifluoroacetic acid (TFA)/triisopropylsilane/water (90 : 5 : 5 (v/v/v), 3 h). The resin was washed with additional TFA (5 mL), and the combined TFA fractions were concentrated to a third of the initial volume under a flow of nitrogen. The crude peptide was then precipitated from cold diethylether (40 mL) and isolated by centrifugation and decantation of the supernatant. The precipitate was washed twice with ice-cold diethylether and subsequently dissolved in 5 mL of a 1 : 1 (v/v) mixture of acetonitrile and water and then freeze-dried to give a fine white solid.

### Peptide purification

Peptides were purified by preparative and semi-preparative reversed-phase HPLC using a JASCO chromatography system (pumps PU-2080 Plus, degasser DG-2080-53, detector MD-2010 Plus) at flow rates of 10 mL min^–1^ and 3 mL min^–1^, respectively, and a Nucleodur 100-5-C18 (250 mm by 21 mm, 5 μm) reversed-phase column from Macherey-Nagel for preparative HPLC and a Nucleodur 100-5-C18 ec, (250 mm by 10 mm, 5 μm) reversed-phase column from Macherey-Nagel for semi-preparative HPLC. Linear gradients of water and acetonitrile (buffer A: water, 0.1% TFA, buffer B: acetonitrile, 0.1% TFA) of 30–60% buffer B over 30 min for A peptides and 20–50% buffer B over 30 min for B peptides were used for purification. Chromatograms were monitored at 220 nm wavelengths.

### Peptide characterization

The peptides were characterized by mass spectrometry on a Bruker Autoflex Speed MALDI-TOF mass spectrometer operating in positive-ion reflector mode (matrix: α-cyano-4-hydroxycinnamic acid (CHCA), external calibration). Analytical HPLC measurements were performed using a JASCO chromatography system (pumps PU-2085 Plus, detector MD-2010 Plus, autosampler AS-2055 Plus) and a Nucleodur 100-5-C18 (250 mm by 4.6 mm, 5 μm) reversed-phase column from Macherey-Nagel at a flow rate of 1 mL min^–1^. For peptide characterization a linear gradient of water and acetonitrile (buffer A: water, 0.1% TFA, buffer B: acetonitrile, 0.1% TFA) run from 20–80% buffer B over 20 min for the A peptides and 10–70% buffer B over 20 min for the B peptides were used. Chromatograms were monitored at 220 nm wavelengths.

### Circular dichroism spectroscopy

CD spectra and CD thermal-denaturation profiles were recorded on a JASCO J-1500 CD spectrometer, which was equipped with a JASCO PTC-510 temperature measuring unit. CD spectra were measured at 50 μM peptide concentration in PBS at 20 °C in 1 mm quartz cuvettes from Starna at 50 nm min^–1^ scanning speed. CD-thermal-denaturation experiments were performed by heating from 5 to 95 °C at a heat rate of 40 °C h^–1^. The CD signal at 222 nm was recorded at 1 °C intervals (1 nm interval, 1 nm bandwidth, 16 s response time). The midpoints of the thermal denaturation curves (*T*_m_) were determined from the second derivative of the variable temperature slope. Equilibrium dissociation constants (*K*_D_) were determined using FitDis! 1.0.^51^

### CD-titration experiments

Coiled-coil peptides were mixed in a 1 : 1 ratio in PBS and in 50 μM concentration of the individual coil peptides. After addition of the competitor peptide in half-equimolar and equimolar amounts, CD spectra were recorded at 20 °C in 1 mm quartz cuvettes from Starna at 50 nm min^–1^ scanning speed. For background correction the coil peptides were measured individually at 25 μM and 50 μM concentration. To process the mixing experiments, the obtained CD spectra were corrected by the CD spectra of the individual peptide, which was displaced in the experiment.

### FRET-assay to determine strand-displacement kinetics

The experiments were performed on a Clariostar plate reader from BMG Labtech equipped with a 1 mL dispenser in black Microfluor 1 96 well plates from Thermo Scientific. Prior to experiment, the well plates were equilibrated with a solution of Roche Blocking Reagent in PBS (1 mg mL^–1^, 300 μL per well) for 2 h at 25 °C. Afterwards, the well plates were washed three times with 100 μL PBS. The fluorescently labelled heterodimeric coiled coils (see Tables S1 and S2† entries 7 to 12) were equilibrated in 15 μM concentration in a 1 : 1 ratio in PBS, first at 25 °C for 1 h, and then at 4 °C over night.

Strand-displacement experiments were performed as triple measurements and monitored over 60 s as a decrease of the FRET-acceptor fluorescence (*λ*_ex_ = 270 nm, *λ*_em_ = 540 nm) upon addition of equimolar amounts of a non-labelled competitor peptide (P_comp_). To obtain minimum fluorescence (*F*_min_), another 50 μM P_comp_ were added. The obtained data was baseline-corrected by *F*_min_, normalized to *F*_max_ and fit to a single exponential decay function using OriginPro 8.5G (eqn (1)),

1 *F* = *F*_eq_ + *A*e^–*k*_obs_/*t*^

where *F*_eq_ is the fluorescence at equilibrium, *A* the pre-exponential factor and *k*_obs_ the observed rate constant. Half-lifes were calculated according to eqn (2).2
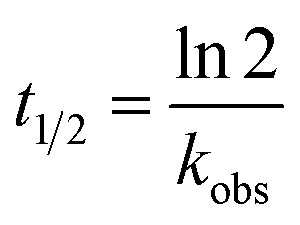



Analysis of the kinetic data was performed by fitting the fluorescence data to a competitive binding model using the program DynaFit.^52^,^53^ DynaFit uses a general numerical method for the determination of rate constants that characterizes simultaneous and competitive binding of a ligand to two receptors presuming a dissociative pathway for receptor displacement. In the context of heterodimeric coiled coils of the type AB the competing receptors can be regarded as A and A_comp_ or as B and B_comp_, respectively. Therefore, strand displacement can be described as follows:
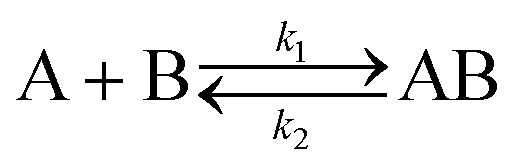
 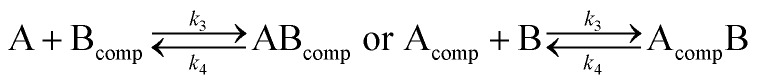


To create the input files for DynaFit, kinetic fluorescence data was converted into concentration of free A or B in the time course of the experiment and competitive reformation of the AB coiled coil. *F*_max_ was assigned to zero conversion of the initial AB coiled-coil complex (15 μM AB and 15 μM P_comp_) and *F*_min_ was assigned to full strand displacement in AB (15 μM AP_comp_ or P_comp_B and 15 μM A or B) to AB_comp_ or A_comp_B, respectively. Same strand-displacement kinetics for coiled coils of the same lengths of the individual strands were presumed whether labelled or unlabelled. Fitting of displacement of A or B from the initial AB complex and re-association, gave relative rate constants *k*_1_ to *k*_4_ from which the overall affinities (*k*_1_*k*_4_/*k*_2_*k*_3_) were calculated.

## Results and discussion

### A set of C-terminally truncated heterodimeric coiled coils

The coiled-coil stability increases with the lengths of the coiled-coil strands as shown for a set of homodimeric coiled coils with chain lengths of one to five heptads.^54^ This can readily be exploited to generally tune the *K*_D_ values of coiled coils. Recently, a set of heterodimeric coiled coils was presented that covered dissociation constants from micromolar to sub-nanomolar concentration (Table 1) as a result from pairing coil strands of 3, 3.5 and 4 heptads in lengths.^37^ Key to the design was a buried asparagine (Asn) pair that not only increased dimer specificity but more importantly aligned the assemblies to form blunt ends at the C-termini and staggered ends at the N-termini. In the following, this set will be dubbed N-truncated set of heterodimeric coiled coils, short N-A_*x*_B_*y*_ with *x* and *y* indicating the lengths of the coil strands, respectively.

**Table 1:** Sequences of N-A_*x*_B_*y*_ and C-A_*x*_B_*y*_ peptides^*a*^

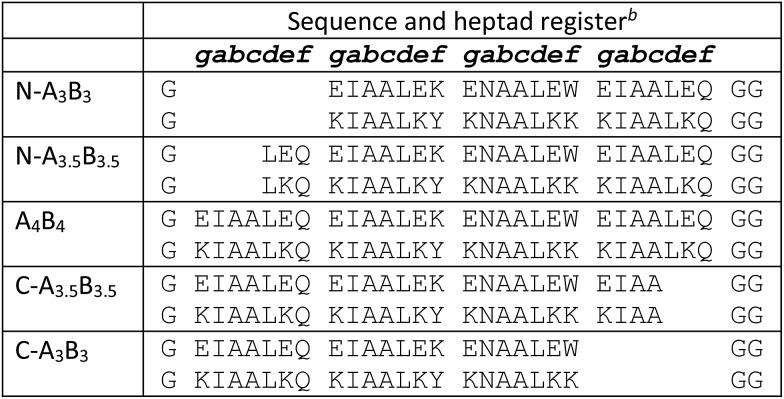

^*a*^For reasons of clarity only the blunt-ended pairs are presented. Combinations of coil strands of different lengths within the N- and C-truncated sets were also studied.

^*b*^Peptides were synthesized as C-terminal amides and were acetylated at the N-termini.

Heterodimeric coiled coils have been widely used as association-mediating tools in biological and biophysical applications.^4^,^55^ To introduce them to *e.g.* biomolecules or surfaces the termini of the coil strands are most commonly addressed. Coiled-coil assemblies with sticky-ended N-termini can complicate designs which rely on N-terminal modification. Therefore, we considered a similar set of heterodimeric coiled coils with varying lengths at the C-termini, C-A_*x*_B_*y*_, as valuable addition to the existing set of heterodimeric coiled-coil building blocks. C-A_*x*_B_*y*_ sequences were designed following the rules for N-A_*x*_B_*y*_,^37^ with the difference that the sequences were shortened from the C-terminal and not from the N-terminal end. In analogy to N-A_*x*_B_*y*_, we chose similar chain lengths and generated a set of nine heterodimeric coiled coils with exclusively blunt-ended N-termini and in parts staggered C-termini (Table 1).

**Fig. 1:**
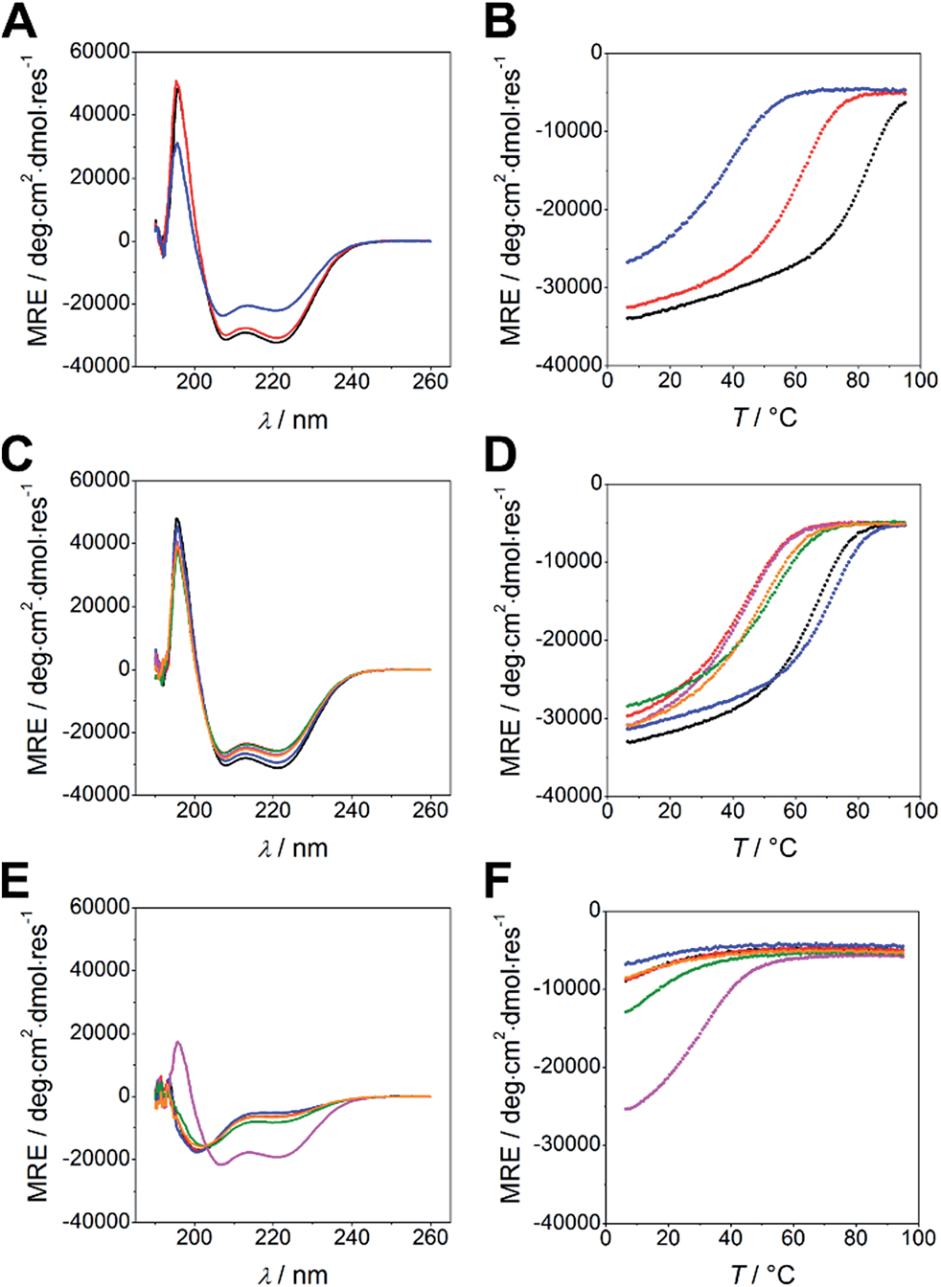
CD spectroscopy and thermal denaturation of C-A_*x*_B_*y*_ peptides. (A) CD spectra and (B) thermal denaturation curves of C-A_*x*_B_*y*_ with equal lengths of the coil strands (C-A_4_B_4_ – black, C-A_3.5_B_3.5_ – red, C-A_3_B_3_ – blue). (C) CD spectra and (D) thermal denaturation curves of C-A_*x*_B_*y*_ with different lengths of the coil strands (C-A_4_B_3.5_ – black, C-A_4_B_3_ – red, C-A_3.5_B_4_ – blue, C-A_3.5_B_3_ – magenta, C-A_3_B_4_ – green, C-A_3_B_3.5_ – orange). (E) CD spectra and (F) thermal denaturation curves of the individual peptides of C-A_*x*_B_*y*_ (C-A_4_ – black, C-A_3.5_ – red, C-A_3_ – blue, C-B_4_ – magenta, C-B_3.5_ – green, C-B_3_ – orange). CD spectra were recorded at 20 °C. All data was measured in PBS at 50 μM peptide concentration.

Peptides were synthesized using microwave-assisted Fmoc/*t*Bu-based solid-phase peptide synthesis and were studied for structure and thermal stability by circular dichroism (CD) spectroscopy. CD spectra of all nine possible combinations of heterodimeric coiled coils show 58 to 85% α-helical structure displayed by the characteristic strong minima at 208 and 222 nm (Fig. 1A and C). Similar to N-A_*x*_B_*y*_ the C-A_*x*_B_*y*_ coiled coils show on-off folding, *i.e.* low tendency to form homomeric assemblies, with the exception of B_4_ which forms weak homodimers at room temperature (Fig. 1E and F).

Thermal denaturation curves were recorded at 222 nm in a temperature gradient from 5–90 °C. All thermal denaturation profiles were obtained as clean sigmoidal curves, from which *T*_M_ values in a range from 38 °C for C-A_3_B_3_ to 81 °C for A_4_B_4_ were calculated (Fig. 1B and D). From the thermal denaturation profiles we determined the dissociation constants *K*_D_ using FitDis! 1.0 by assuming a two-state unfolding process for our heterodimeric coiled coils.^51^ The *K*_D_ values for C-A_*x*_B_*y*_ range from micromolar to sub-nanomolar concentration and are in accordance with the *K*_D_ values obtained for the N-truncated series (Table 2, Fig. S2†). However, *K*_D_ values of N-A_*x*_B_*y*_ were determined differently, more specifically, by recording a series CD-thermal-denaturation profiles across a range of peptide concentrations and linearizing the concentration-dependent melting temperatures to define the *K*_D_ at any given temperature. To ensure comparability, we decided to re-determine the *K*_D_ values of N-A_*x*_B_*y*_ using FitDis! 1.0 and were able to confirm a good correlation with the literature-reported values (Table 3, Fig. S1†).^37^

**Table 2:** Melting temperatures (*T*_M_) and dissociation constants *K*_D_ of C-A_*x*_B_*y*_ peptides^*a*^

*T*_M_ (°C)/*K*_D_ (M)	C-A_3_	C-A_3.5_	C-A_4_
C-B_3_	38.3 ± 0.4/(4.9 ± 3.5) × 10^–6^	43.8 ± 0.4/(2.8 ± 0.5) × 10^–6^	44.1 ± 0.7/(3.1 ± 1.5) × 10^–6^
C-B_3.5_	48.6 ± 0.7/(1.5 ± 0.3) × 10^–6^	62.6 ± 0.4/(8.9 ± 5.8) × 10^–8^	66.5 ± 0.4/(5.5 ± 2.4) × 10^–8^
C-B_4_	52.6 ± 0.4/(8.6 ± 2.1) × 10^–7^	72.3 ± 0.7/(7.3 ± 1.1) × 10^–9^	81.0 ± 0.7/(5.7 ± 1.06) × 10^–10^

^*a*^ *K*_D_s were determined with FitDis! 1.0 (ref. 51) from CD thermal denaturation profiles at 50 μM peptide concentration except for A_4_B_4_, where a CD thermal denaturation profile measured at 25 μM peptide concentration was used to improve the fit.

**Table 3:** Dissociation constants *K*_D_ of N-A_*x*_B_*y*_ peptides^*a*^

*K*_D_ (M)	N-A_3_	N-A_3.5_	N-A_4_
N-B_3_	(4.5 ± 2.2) × 10^–6^	(8.0 ± 7.2) × 10^–7^	(2.5 ± 1.9) × 10^–7^
N-B_3.5_	(1.9 ± 1.1) × 10^–6^	(6.4 ± 3.7) × 10^–9^	(9.2 ± 5.9) × 10^–10^
N-B_4_	(8.8 ± 3.5) × 10^–7^	(1.0 ± 1.1) × 10^–9^	(5.7 ± 1.1) × 10^–10^

^*a*^ *K*_D_s were determined with FitDis! 1.0 (ref. 51) from CD thermal denaturation profiles at 50 μM peptide concentration except for A_4_B_4_, where a CD thermal denaturation profile measured at 25 μM peptide concentration was used to improve the fit. *T*_M_ values have been reported by Thomas *et al.*^37^

Comparison of *T*_M_ and *K*_D_ values for C-A_*x*_B_*y*_ and the N-truncated set of heterodimeric coiled coils revealed slightly reduced coiled-coil stabilities and less well distributed *K*_D_ values. For instance, the *T*_M_ and *K*_D_ of C-A_3.5_B_3_ are equal to the *T*_M_ and *K*_D_ of C-A_4_B_3_. Although stabilities of the heterodimeric coiled coils generally increase with an increase of the length of the hydrophobic core, the lengths of the peptide overhangs at the C-termini apparently have less impact on the overall stabilities than at the N-termini. Interestingly, assemblies with B-peptide overhang are slightly more stable than assemblies, in which the A peptide is longer. This is likely due to the helix macrodipole,^56^ which accounts for a partially positively charged N-terminus and a partially negatively charged C-terminus. Therefore, positively charged amino acids are structure stabilizing when located at the C-terminus and destabilizing when located at the N-terminus. The opposite effects are observed for negatively charged amino acids. If this is the case, slightly higher structure stabilities should have been found for N-A_*x*_B_*y*_ with longer A peptides and have indeed been reported.^37^

### The thermally more stable coiled coil is formed in CD-titration experiments

**Fig. 2:**
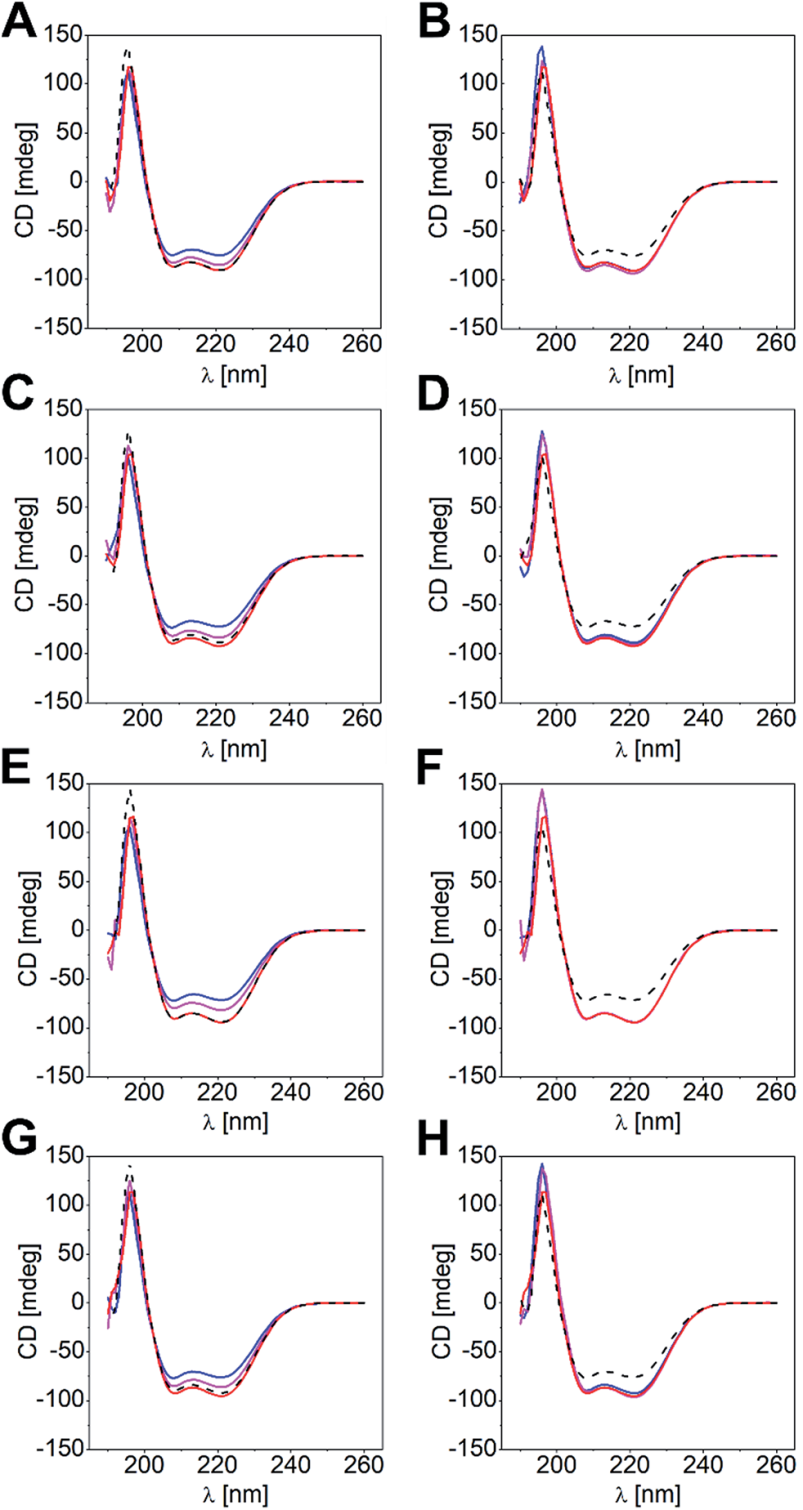
CD spectroscopy of coiled-coil titration experiments. (A) Titration of N-B_3.5_ to N-A_4_B_3_, (B) titration of N-B_3_ to N-A_4_B_3.5_, (C) titration of N-A_3.5_ to N-A_3_B_4_, (D) titration of N-A_3_ to N-A_3.5_B_4_, (E) titration of C-B_3.5_ to C-A_4_B_3_, (F) titration of C-B_3_ to C-A_4_B_3.5_, (G) titration of C-A_3.5_ to C-A_3_B_4_, (H) titration of C-A_3_ to C-A_3.5_B_4_, (0 eq. P_comp_ – blue, 0.5 eq. P_comp_ – magenta, 1 eq. P_comp_ – red, reference spectrum for complete displacement by P_comp_ – black, dashed line). Conditions: 50 μM N/C-A_*x*_B_*y*_, PBS, 20 °C. CD data of the displaced coil peptide was used for baseline correction.

Differences in *K*_D_ values led us to conclude that shorter coil strands could be displaced by the longer ones to give the more stable heterodimeric coiled coil. Likewise, the most stable coiled-coil assembly out of a peptide mixture of three different coil peptides should form in solution. To substantiate this hypothesis, we performed CD-titration experiments of a heterodimeric coiled coil and a competitor peptide (P_comp_: A_comp_, B_comp_), and analyzed the peptide mixture by CD spectroscopy. We determined mixtures with P_comp_ added in half-equimolar and equimolar amounts, respectively. In Fig. 2, the overlaid spectra of four titration experiments are exemplarily shown, namely (a) titration of N-B_3.5_ to N-A_4_B_3_ and v.v. (Fig. 2A and B), (b) titration of N-A_3.5_ to N-A_3_B_4_ and v.v. (Fig. 2C and D), (c) titration of C-B_3.5_ to C-A_4_B_3_ and v.v. (Fig. 2E and F), and (d) titration of C-A_3.5_ to C-A_3_B_4_ and v.v. (Fig. 2G and H; for further results, see Fig. S3 and S4†). The obtained CD data has been corrected by the CD spectra of the supposedly displaced coil strands and compared with the CD spectra of the two competing heterodimeric coiled coils.

As clearly documented in Fig. 2, strand displacement from a coiled-coil assembly is possible in both sets of heterodimeric coiled coils, if the competing peptide interacts more strongly with the complementary coil strand (Fig. 2A, C, E and G). Titration of the respective peptides to the coiled-coil assemblies resulted in a gradual change of the CD spectra, which, in a 1 : 1 ratio of the coiled-coil assembly and the competitor peptide, superimposed well with the expected CD spectra of the more stable coiled-coil assemblies (overlay: black dotted line and red line). In contrast, titration of a shorter and more weakly interacting competitive peptide did not result in significant changes of the CD spectra.

Admittedly, the aforementioned examples reveal differences in *K*_D_ values of about two magnitudes; hence, the driving force of a strand displacement in favour for the more stable coiled-coil pair is high. However, we investigated all possible coiled-coil combinations within N-A_*x*_B_*y*_ and C-A_*x*_B_*y*_, respectively, and found that displacement of shorter coil strands by longer competing peptides is the most frequent scenario. Nonetheless, if the *K*_D_ values were very similar, the mixing experiments gave more intense CD signals, compared to the CD spectra of the two competing heterodimeric coiled-coil pairs (*e.g.* Fig. S3E and F or Fig. S4C and D†). Furthermore, the results were inconclusive for some combinations as the CD spectra of the competing heterodimeric coiled coils were too similar (*e.g.* Fig. S3M and N or Fig. S4Y and Z†).

### Strand displacement in heterodimeric coiled coils follows a competitive binding mechanism

CD-titration experiments indicated the possibility of strand displacement in two competing coiled-coil assemblies. However, this data is purely indicative and not quantifiable. Furthermore, the experiments were performed at reasonably high peptide concentrations and do not reveal the displacement mechanism. Many applications require low peptide concentrations; hence, insights into the kinetics of strand-displacement processes in coiled coils are a requirement for reliable coiled-coil-based designs. Studying the displacement mechanism should provide information about the speed of strand displacement in two competing *de novo* designed heterodimeric coiled coils, and the necessary *K*_D_ difference at which to expect complete strand displacement in favour for the more stable coiled-coil assembly.

Although the coiled-coil interaction is widely understood, studies of the mechanism of strand displacement in heterodimeric coiled coils are scarce. For coiled-coil designs used in coiled-coil ligases displacement *via* a triple-stranded intermediate is anticipated; however, the designs are promiscuous on purpose to maintain a defined and predictable three-dimensional structure during this process.^57^ The heterodimeric coiled coils used herein are non-promiscuous by design, hence, higher-order oligomers during the displacement process can be excluded. We therefore assumed that displacement proceeds *via* a dissociative mechanism.

To approach this problem, we considered a FRET-based strand-displacement assay using fluorescently labelled heterodimeric coiled coils as reporter peptides and non-labelled A and B peptides as competitors. As the introduction of fluorescence labels can influence the coiled-coil interaction,^58^,^59^ we decided to use small fluorescent dyes, more precisely, intrinsically fluorescent tryptophan as FRET-donor and Dansyl as FRET-acceptor. Furthermore, the chromophores were placed at a central *f*-position of the respective coiled-coil strands to avoid fluorophore-related stabilization of the overall coiled-coil assembly (Tables S1 and S2†). Generally, A peptides were labelled intrinsically with tryptophan and B peptides with Dansyl by modifying the side chain of an *f*-positioned lysine. As competitor peptides parent B peptides were suited as B_comp_ (Tables S1 and S2,† entries 4–6), whereas for A_comp_ chromophore-free A peptides were synthesized (Tables S1 and S2,† entries 13–15).

**Fig. 3:**
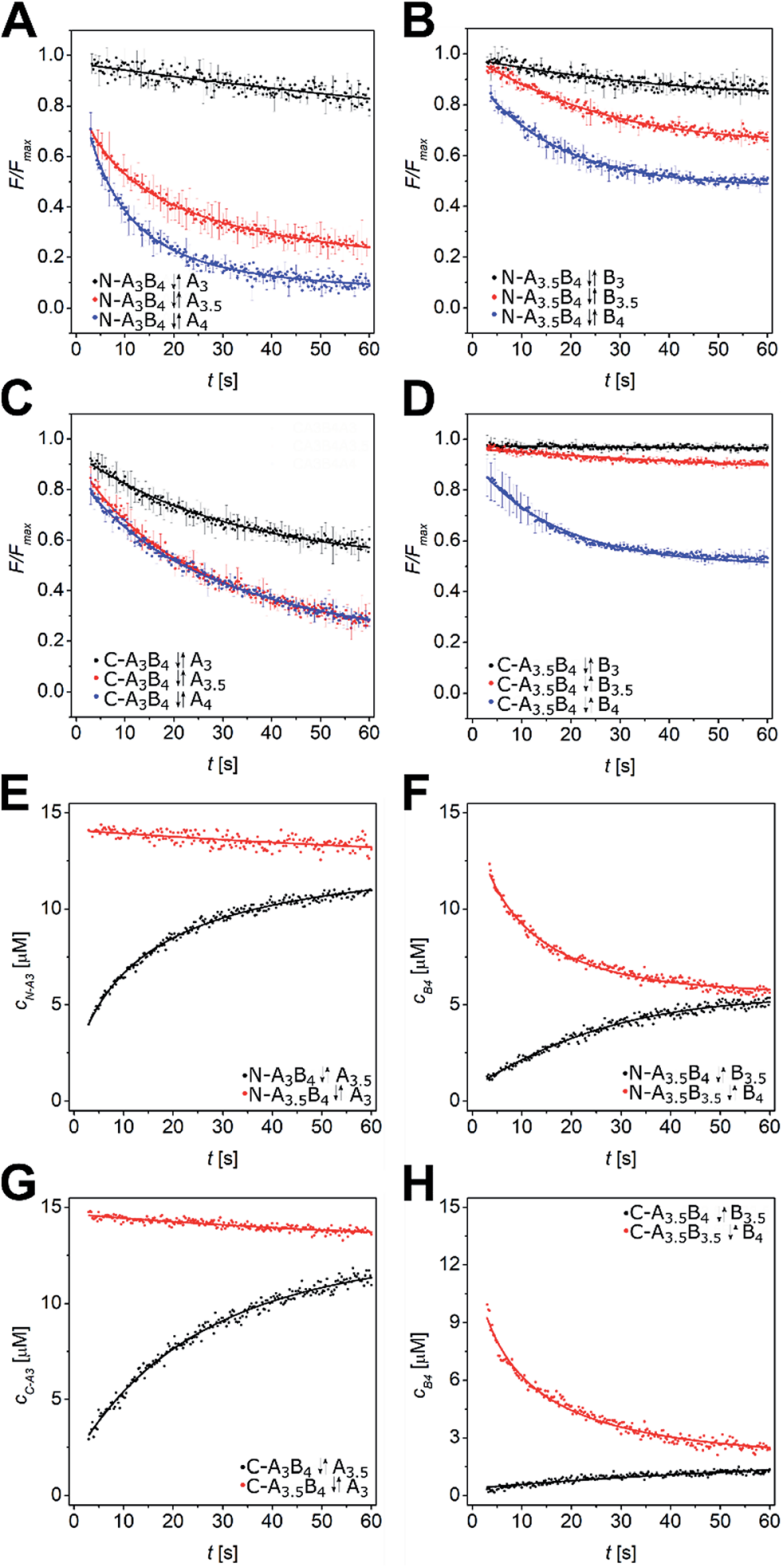
Strand-displacement kinetics in heterodimeric coiled coils. (A–D) Normalized time-resolved fluorescence decrease upon strand displacement in A_*x*_B_*y*_ peptides. Exemplarily, strand displacements in N-A_3_B_4_ by N-A_comp_ (A), N-A_3.5_B_4_ by N-B_comp_ (B), C-A_3_B_4_ by C-A_comp_ (C), and C-A_3.5_B_4_ by C-B_comp_ (D) are shown. Data is fit by a single exponential decay model. (E–H) Least-squares fits of strand displacement in N-A_3_B_4_ by A_3.5_ (E), N-A_3.5_B_4_ by B_3.5_ (F), C-A_3_B_4_ by A_3.5_ (G), and C-A_3.5_B_4_ by B_3.5_ (H) using a competitive binding model in DynaFit^53^ (forward reaction – black, backward reaction – red). Conditions: 15 μM peptide concentration, PBS (pH 7.4), room temperature, readout: *λ*_ex_ = 270 nm, *λ*_em_ = 540 nm. Measurements were performed as triplicates.

We performed a time-resolved FRET-based displacement assay by mixing labelled A and B peptides at 15 μM peptide concentration and monitoring the FRET signal as a decrease of acceptor fluorescence over 60 s upon injection of equimolar amounts of P_comp_. The obtained data was background corrected by the minimum FRET fluorescence (*F*_min_), and normalized by maximum FRET fluorescence *F*_max_. In case of partial or complete strand displacement, a decrease of the FRET signal was observed approaching a plateau in most of the measurements. This is explained by dissociation of the preformed labelled coiled coil and re-association to a mixture of heterodimeric coiled coils depending on the rates of dissociation and association of the respective coiled-coil assemblies. The kinetic data were fit well by a single exponential model (see ESI†) with observed exchange rates (*k*_obs_) from 0.01 s^–1^ to 0.1 s^–1^ and respective half-lifes from 7 s to 70 s (Tables S5 and S6†). In Fig. 3A–D the strand-displacement kinetics of N-A_3_B_4_-A_comp_, N-A_3.5_B_4_-B_comp_, C-A_3_B_4_-A_comp_, and C-A_3.5_B_4_-B_comp_ are shown. It is clearly noticeable that strand displacement is more likely with larger difference in *K*_D_. The length of the competitor strand is less important as demonstrated by displacement of the B_4_-coil strand in N-A_3.5_B_4_ by N-B_3.5comp_ (Fig. 3B). B_4_ is significantly displaced although N-B_3.5comp_ is shorter. However, the *K*_D_ value of competing N-A_3.5_B_3.5_ is only marginally higher (Table 2), hence, at equilibrium, a mixture of both assemblies is present.

**Fig. 4:**
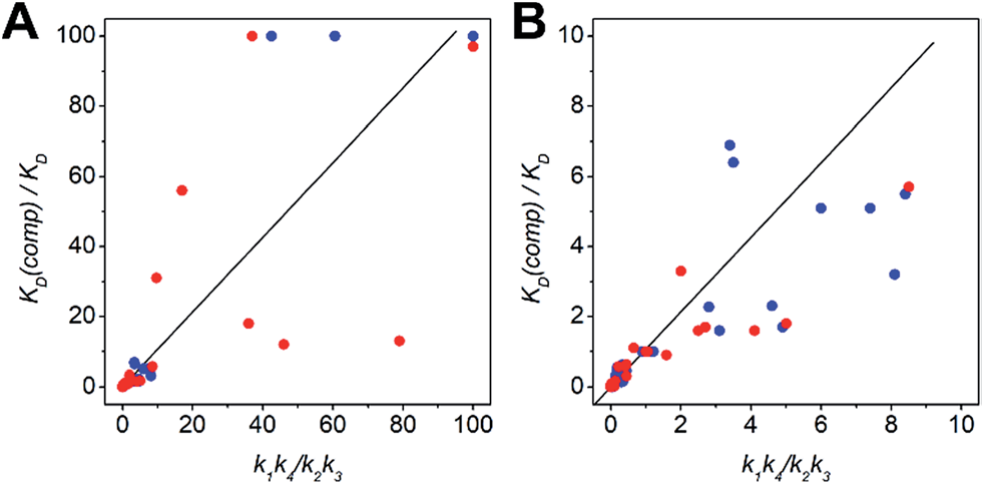
Scatter plots depicting the correlation between the ratio of *K*_D_ values and overall affinities. (A) Scatter plot including ratios upto 100. (B) Low-range scatter plot (colour-key: N-A_*x*_B_*y*_ – blue, C-A_*x*_B_*y*_ – red). The *K*_D_ values were determined by CD-thermal denaturation experiments. The overall affinities (*k*_1_*k*_4_/*k*_2_*k*_3_) result from processing of the kinetic data to a competitive binding mechanism in DynaFit. For clarity, ratios > 100 are not depicted in the plots. This data is shown in Tables S7 and S8.†

The kinetic data from the FRET assay revealed an equilibrium reaction for strand displacement in heterodimeric coiled coils, which is interpreted as a competition of P_comp_ and the respective coil strand for association to the complementary coil strand. As mentioned above, we hypothesized a dissociative mechanism for the strand displacement. We assumed that a competition model should describe strand displacement in good approximation, although coiled-coil interaction is obligate. Therefore, the measured kinetic data were fitted to competitive binding mechanism using the program DynaFit.^52^,^53^ As changes in concentration of free displaced (forward reaction) and replaced (backward reaction) A or B peptide are fit, we converted the FRET-acceptor fluorescence signal to peptide concentration to generate the input files required for DynaFit as described in the Materials and methods section. Simultaneous fitting of both events, displacement of A or B from the initial AB coiled coil and reassociation, gave rate constants *k*_1_ to *k*_4_ from which the overall affinities (*k*_1_*k*_4_/*k*_2_*k*_3_) were calculated (Tables S7 and S8†). As exemplarily shown in Fig. 3, the competitive binding model applied well to the strand-displacement data and the obtained overall affinities showed a good correlation to the ratio of *K*_D_ values obtained from CD thermal denaturation curves (Fig. 4, Tables S7 and S8†). This supports the competition model for strand displacement in heterodimeric coiled coils. The ratio of the *K*_D_ values of the two competing coiled coils should give the equilibrium concentration and therefore, should be suitable to predict the outcome of a displacement reaction.

We performed more than 100 displacement reactions, from which we were able to deduce an assignment of the obtained values. In Fig. 3E–H four typical graphical outputs of our DynaFit data analysis are shown with the black traces depicting the forward reactions and the red traces the backward reactions. Except for N-A_3.5_B_3.5_-B_4_ (Fig. 3F) the positions of strand-displacement equilibria lie either fully on the side of the strand-displacement products (Fig. 3E and G) or on the side of the strand-displacement educts (Fig. 3H). If this is the case, the overall affinities gave either values less than 0.1 for complete or almost complete strand displacement or greater than 10.0 for no or only little strand displacement. If strand displacement reaction resulted in a mixture of both competing heterodimeric coiled coils at equilibrium (Fig. 3F), overall affinities gave values between 10.0 and 0.1. We state that these ranges of overall affinities or *K*_D_ values can be applied as a reliable rule-of-thumb to assess strand-displacement equilibria in any competing heterodimeric coiled coils, if the assemblies are non-promiscuously heterodimeric.

## Conclusion

In summary, we have presented a thorough study on single strand-displacement kinetics in *de novo* designed parallel heterodimeric coiled coils. We investigated two sets of well-characterized heterodimeric coiled coils, which exhibit dissociation constants from the μM to the sub-nM regime. Peptide design is based on Hodges EK peptides,^31^ but is extended by Asn residues at a central *a* position to increase the specificity for parallel-oriented dimeric assemblies. Furthermore, this additional design feature allows formation of defined coiled-coil assemblies with variable chain lengths, a key-requirement for easy modulation of the strength of coiled-coil association. Design and biophysical characterization of the N-A_*x*_B_*y*_ peptides have been previously reported.^37^ Variation of *K*_D_ values was achieved by truncating the individual coiled-coil strands from the N-terminal end. We expanded the existing set of well-characterized heterodimeric coiled coils by adding C-A_*x*_B_*y*_, which is based on the same design rules as N-A_*x*_B_*y*_, except the fine-tuning of the strength of association is achieved by truncating the individual coil strands from the C-terminal end. We consider C-A_*x*_B_*y*_ as a useful addition to the coiled-coil toolkit as blunt-ended N-termini facilitate applications, in which N-terminal modified coiled coils are involved. C-A_*x*_B_*y*_ peptides were characterized by CD spectroscopy, and *K*_D_ values were determined from CD-thermal-denaturation profiles. Although the resulting assemblies showed an overall slightly reduced stability compared to N-A_*x*_B_*y*_ the determined *K*_D_ values covered a similar range of concentrations.

We studied strand displacement in both sets of heterodimeric coiled coils. CD-titration experiments indicated a preference for the formation of the thermodynamically more stable coiled coil in most of the studied combinations. However, coiled-coil mixtures with similar *K*_D_ values of the two competing assemblies gave inconclusive CD data. Using a FRET-based assay, we characterized strand displacements in our sets of heterodimeric coiled coils as equilibrium reactions with half-lifes of about 7 to 70 s. The kinetic data were fit well by a competitive binding model from which the rate constants of association and dissociation of the competing coiled-coil assemblies, and hence, the overall affinities were obtained. The overall affinities correlate well with the respective ratio of *K*_D_ values obtained from CD data supporting the competitive binding mechanism of the strand displacement in heterodimeric coiled coils.

The ratio of *K*_D_ values can be used to predict the outcome of a strand-displacement reaction. Based on the results of 108 possible strand-displacement reactions, we were able to correlate the ratio of *K*_D_ values to the position of the strand-displacement equilibrium and to classify three categories: *K*_D_ ratios of above 10.0 correlate with very slow to no strand displacement, whereas ratios below 0.1 indicate complete displacement reactions. With ratios of *K*_D_ values between 10.0 and 0.1 the formation of coiled-coil mixtures is highly probable.

Our aim was to find a reliable rule to assess strand displacement in heterodimeric coiled coils. Very often the *K*_D_ values of coiled coils are known and discussed as potential parameters to predict strand displacement.^8^,^37^ Best to our knowledge, there is only one very recent study that correlates *K*_D_ differences and strand displacement;^49^ however, only selected coiled coils were investigated and an analysis of the underlying mechanism has not been undertaken. Furthermore, the coiled coils applied in that study have previously been proven to form promiscuous assemblies as they are lacking the required core-asparagine residues.^35^–^37^ We studied an extended coiled-coil library and thus were able to identify the reaction mechanism, which allows us to use the ratio of *K*_D_ values of the competing coiled coils as parameter to reliably predict the outcome of a strand-displacement reaction without further kinetic experiments. We believe that the abovementioned rule of thumb is easy to adapt to other sets of heterodimeric coiled coils based on similar designs, and hence, will facilitate future designs of dynamic coiled-coil-based systems.

## Conflicts of interest

There are no conflicts to declare.

## Supplementary Material

Supplementary information
