# Supplementary material for: Kinetic studies on strand displacement in de novo designed parallel heterodimeric coiled coils
Source: Chem Sci. 2018 Apr 17;9(18):4308–16. doi: 10.1039/c7sc05342h (PMC5944379; doi:10.1039/c7sc05342h)
Supplement: Supplementary file 1 [file SC-009-C7SC05342H-s001.pdf]

Electronic Supplementary Material (ESI) for Chemical Science.
This journal is © The Royal Society of Chemistry 2018

Supporting Information

Kinetic studies on strand displacement in de novo designed parallel heterodimeric coiled coils

Mike C. Groth,[a] W. Mathis Rink,[a] Nils F. Meyer,[a] and Franziska Thomas*[a, b]

[a]Institute of Organic and Biomolecular Chemistry, Georg-August-Universität Göttingen, Tammannstr. 2, 37077 Göttingen, Germany

[b]Center for Biostructural Imaging of Neurodegeneration, von-Siebold-Straße 3a, 37075 Göttingen, Germany

*To correspondence should be addressed: fthomas@gwdg.de

Table of Content

1. Peptide sequences....S2
2. Determination of unfolding constants S4
3. CD-titration experiments....S10
4. FRET-based strand-displacement assay S22
5. MALDI-TOF mass spectrometry and HPLC S52
6. CD spectroscopy and thermal denaturation of *C*-truncated heterodimeric coiled coils S59
7. References....S64

1. Peptide sequences

Table S1. Sequences of the *N*-terminally truncated set of *de novo* designed heterodimeric coiled coils. K^*-Dansyl-labelled lysine residue.

	**N-A_xB_y**	**Sequence and heptad register[b]**
		gabcdef gabcdef gabcdef gabcdef
**1**	N-A_3	G EIAALEK ENAALEW EIAALEQ GG
**2**	N-$A_{3.5}$	G LEQ EIAALEK ENAALEW EIAALEQ GG
**3**	A_4	G EIAALEQ EIAALEK ENAALEW EIAALEQ GG
**4**	N-B_3	G KIAALKY KNAALKK KIAALKQ GG
**5**	N-$B_{3.5}$	G LKQ KIAALKY KNAALKK KIAALKQ GG
**6**	B_4	G KIAALKQ KIAALKY KNAALKK KIAALKQ GG
**7**	N-A_3^*	G EIAALEW ENAALEK EIAALEQ GG
**8**	N-$A_{3.5}^*$	G LEQ EIAALEW ENAALEK EIAALEQ GG
**9**	A_4^*	G EIAALEQ EIAALEW ENAALEK EIAALEQ GG
**10**	N-B_3^*	G KIAALKK* KNAALKK KIAALKQ GG
**11**	N-$B_{3.5}^*$	G LKQ KIAALKK* KNAALKK KIAALKQ GG
**12**	B_4^*	G KIAALKQ KIAALKK* KNAALKK KIAALKQ GG
**13**	N-A_3_W15K (N-A_{3comp})	G EIAALEK ENAALEK EIAALEQ GG
**14**	N-$A_{3.5}$_W18K (N-$A_{3.5comp}$)	G LEQ EIAALEK ENAALEK EIAALEQ GG
**15**	A_4_W22K (A_{4comp})	G EIAALEQ EIAALEK ENAALEK EIAALEQ GG

Table S2. Sequences of the *C*-terminally truncated set of *de novo* designed heterodimeric coiled coils. K*-Dansyl-labelled lysine residue.

	$C\text{-}A_xB_y$	Sequence and heptad register[b]
		gabcdef gabcdef gabcdef gabcdef
**1**	$C\text{-}A_3$	`G EIAALEQ EIAALEK ENAALEW GG`
**2**	$C\text{-}A_{3.5}$	`G EIAALEQ EIAALEK ENAALEW EIAA GG`
**3**	A_4	`G EIAALEQ EIAALEK ENAALEW EIAALEQ GG`
**4**	$C\text{-}B_3$	`G KIAALKQ KIAALKY KNAALKK GG`
**5**	$C\text{-}B_{3.5}$	`G KIAALKQ KIAALKY KNAALKK KIAA GG`
**6**	B_4	`G KIAALKQ KIAALKY KNAALKK KIAALKQ GG`
**7**	$C\text{-}A_3^*$	`G EIAALEQ EIAALEW ENAALEK GG`
**8**	$C\text{-}A_{3.5}^*$	`G EIAALEQ EIAALEW ENAALEK EIAA GG`
**9**	A_4^*	`G EIAALEQ EIAALEW ENAALEK EIAALEQ GG`
**10**	$C\text{-}B_3^*$	`G KIAALKQ KIAALKK* KNAALKK GG`
**11**	$C\text{-}B_{3.5}^*$	`G KIAALKQ KIAALKK* KNAALKK KIAA GG`
**12**	B_4^*	`G KIAALKQ KIAALKK* KNAALKK KIAALKQ GG`
**13**	$C\text{-}A_3$_W22K ($C\text{-}A_{3comp}$)	`G EIAALEQ EIAALEK ENAALEK GG`
**14**	$C\text{-}A_{3.5}$_W22K ($C\text{-}A_{3.5comp}$)	`G EIAALEQ EIAALEK ENAALEK EIAA GG`
**15**	A_4_W22K (A_{4comp})	`G EIAALEQ EIAALEK ENAALEK EIAALEQ GG`

A_4 and B_4 are the same in both sets of heterodimeric coiled coils.

2. Determination of unfolding constants

Table S3. Equilibrium constants of dissociation K_D of the N-A_xB_y peptides obtained from the thermal denaturation curves at 50 µM coiled-coil concentration (N-A_3B_3 – N-$A_4B_{3.5}$) and 25 µM (A_4B_4) using FitDis! analysis software.[1]

**K_D (M)**	**N-A_3**	**N-$A_{3.5}$**	**A_4**
**N-B_3**	$(4.5 \pm 2.2)\ 10^{-6}$	$(8.0 \pm 7.2)\ 10^{-7}$	$(2.5 \pm 1.9)\ 10^{-7}$
**N-$B_{3.5}$**	$(1.9 \pm 1.1)\ 10^{-6}$	$(6.4 \pm 3.7)\ 10^{-9}$	$(9.2 \pm 5.9)\ 10^{-10}$
**B_4**	$(8.8 \pm 3.5)\ 10^{-7}$	$(1.0 \pm 1.1)\ 10^{-9}$	$(5.7 \pm 1.1)\ 10^{-10}$

Table S4. Equilibrium constants of dissociation K_D of the C-A_xB_y peptides obtained from the thermal denaturation curves at 50 µM coiled-coil concentration (C-A_3B_3 – C-$A_4B_{3.5}$) and 25 µM (A_4B_4) using FitDis! analysis software.[1]

**K_D (M)**	**C-A_3**	**C-$A_{3.5}$**	**A_4**
**C-B_3**	$(4.9 \pm 3.5) \cdot 10^{-6}$	$(2.8 \pm 0.5) \cdot 10^{-6}$	$(3.1 \pm 1.5) \cdot 10^{-6}$
**C-$B_{3.5}$**	$(1.5 \pm 3.0) \cdot 10^{-6}$	$(8.9 \pm 5.8) \cdot 10^{-8}$	$(5.5 \pm 2.4) \cdot 10^{-8}$
**B_4**	$(8.6 \pm 2.1) \cdot 10^{-7}$	$(7.3 \pm 1.1) \cdot 10^{-9}$	$(5.7 \pm 1.06)\ 10^{-10}$

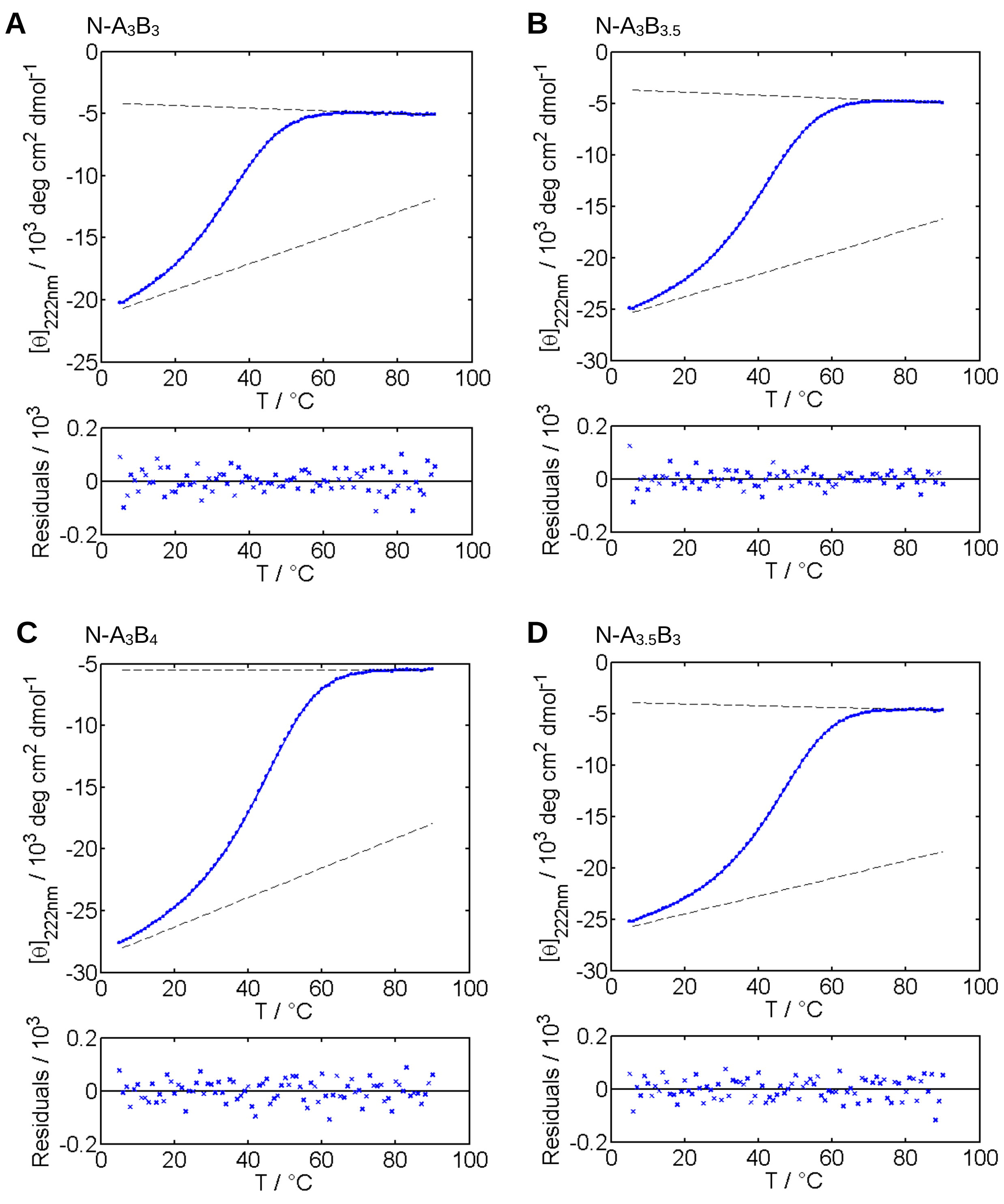
A
N-A3B3
B
N-A3B3.5
C
N-A3B4
D
N-A3.5B3
[θ]222nm / 10^3 deg cm^2 dmol^-1
Residuals / 10^3
T / °C

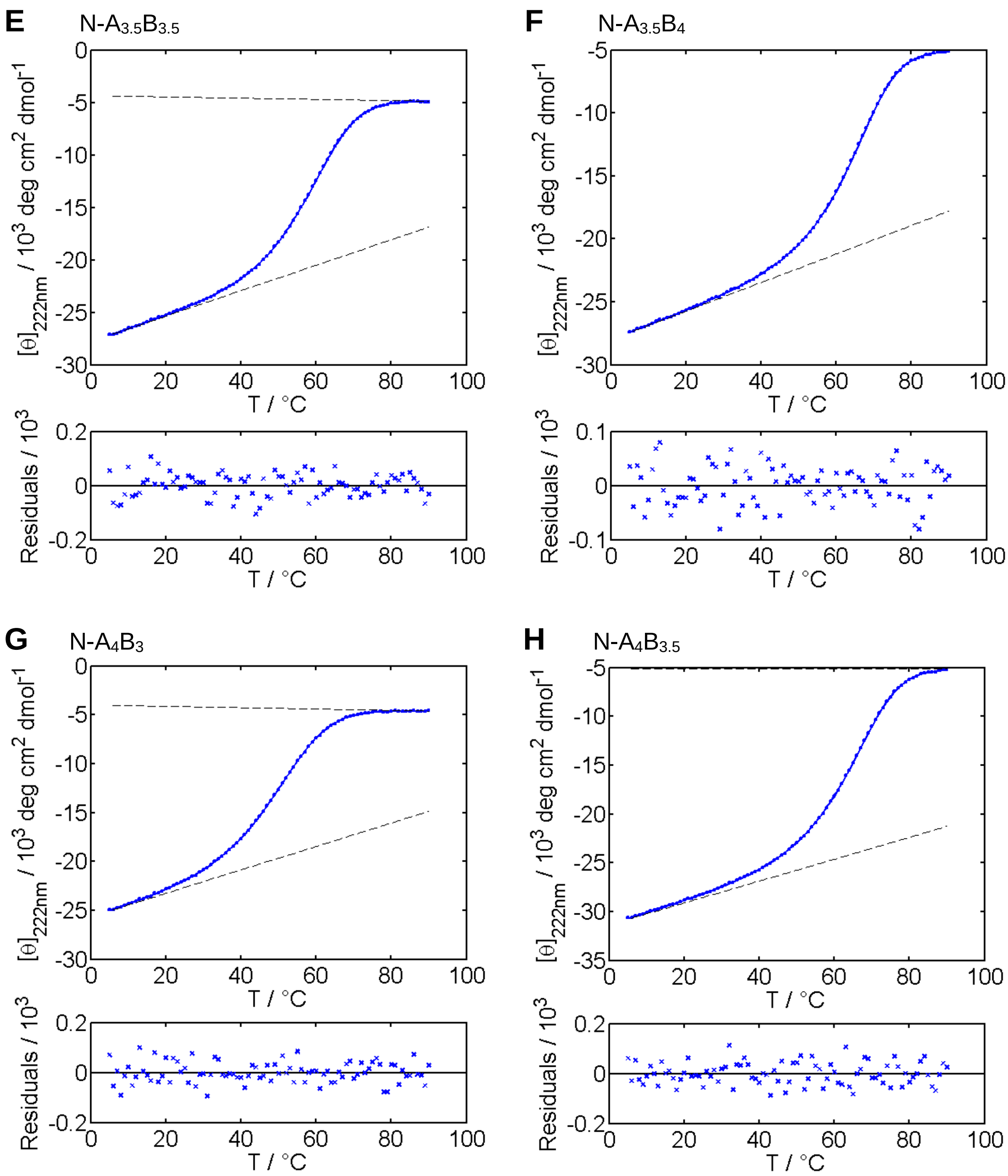
E
N-A3.5B3.5
F
N-A3.5B4
G
N-A4B3
H
N-A4B3.5
[θ]222nm / 10^3 deg cm^2 dmol^-1
Residuals / 10^3
T / °C

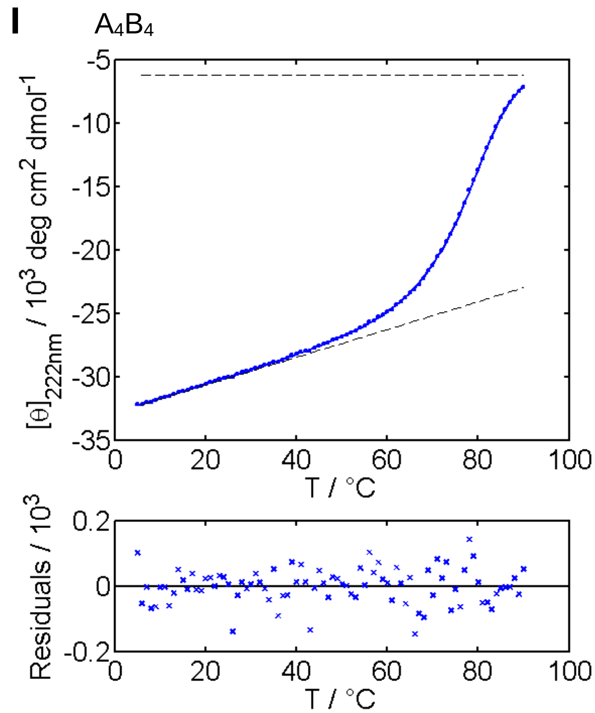

Figure S1. CD-thermal denaturation curves of the N-A_xB_y peptides fit to a two-state unfolding model (top) and residuals depicting the quality of the fit (bottom). A) N-A_3B_3, B) N-$A_3B_{3.5}$, C) N-A_3B_4, D) N-$A_{3.5}B_3$, E) N-$A_{3.5}B_{3.5}$, F) N-$A_{3.5}B_4$, G) N-A_4B_3, H) N-$A_4B_{3.5}$, I) A_4B_4. Thermal denaturation curves were recorded at 50 μM coiled-coil concentration (A_4B_4: 25 μM coiled-coil concentration) in PBS buffer. Data were processed using the FitDis! analysis software.[1]

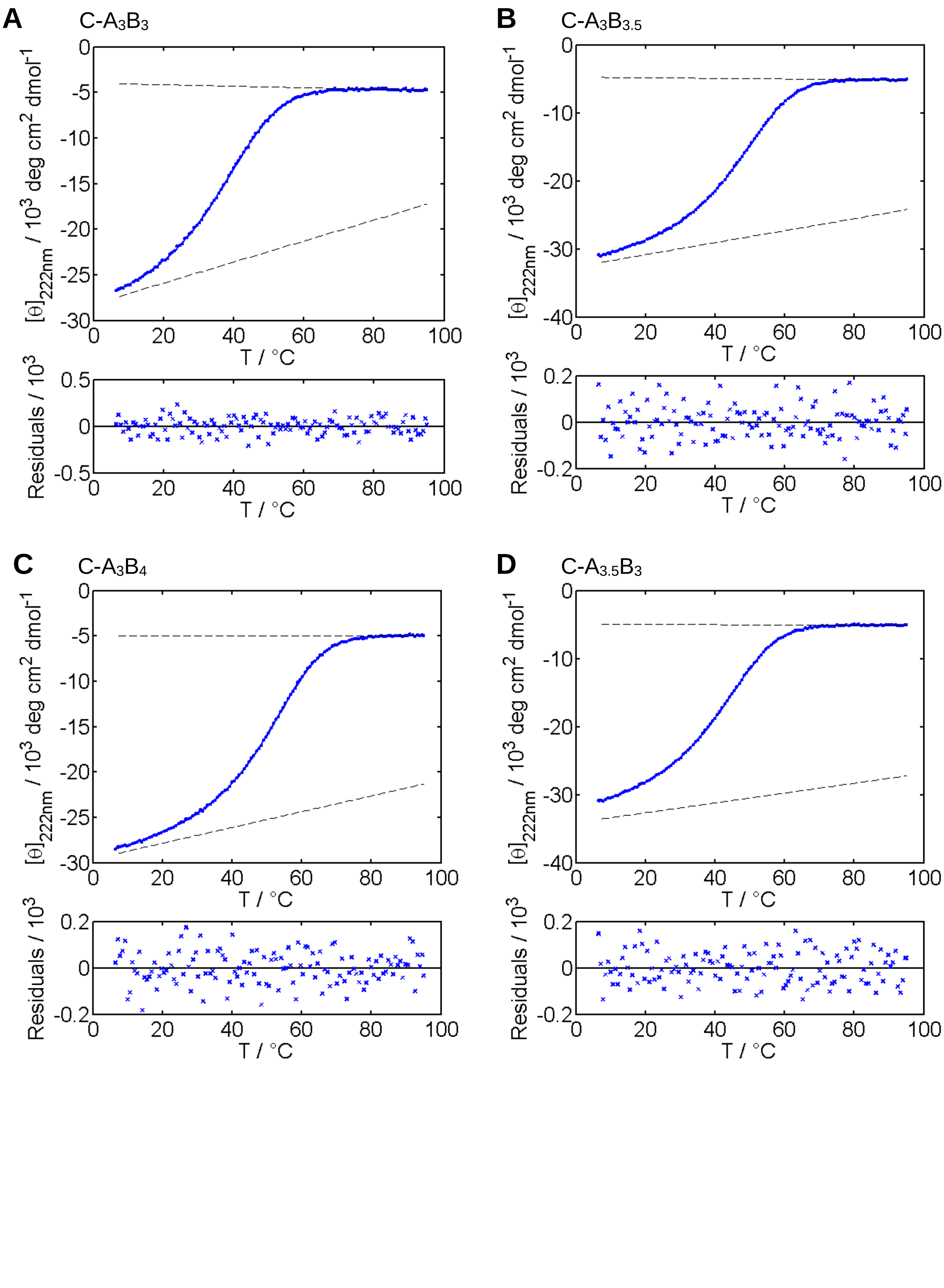
A
C-A3B3
B
C-A3B3.5
C
C-A3B4
D
C-A3.5B3
[θ]222nm / 10^3 deg cm^2 dmol^-1
Residuals / 10^3
T / °C

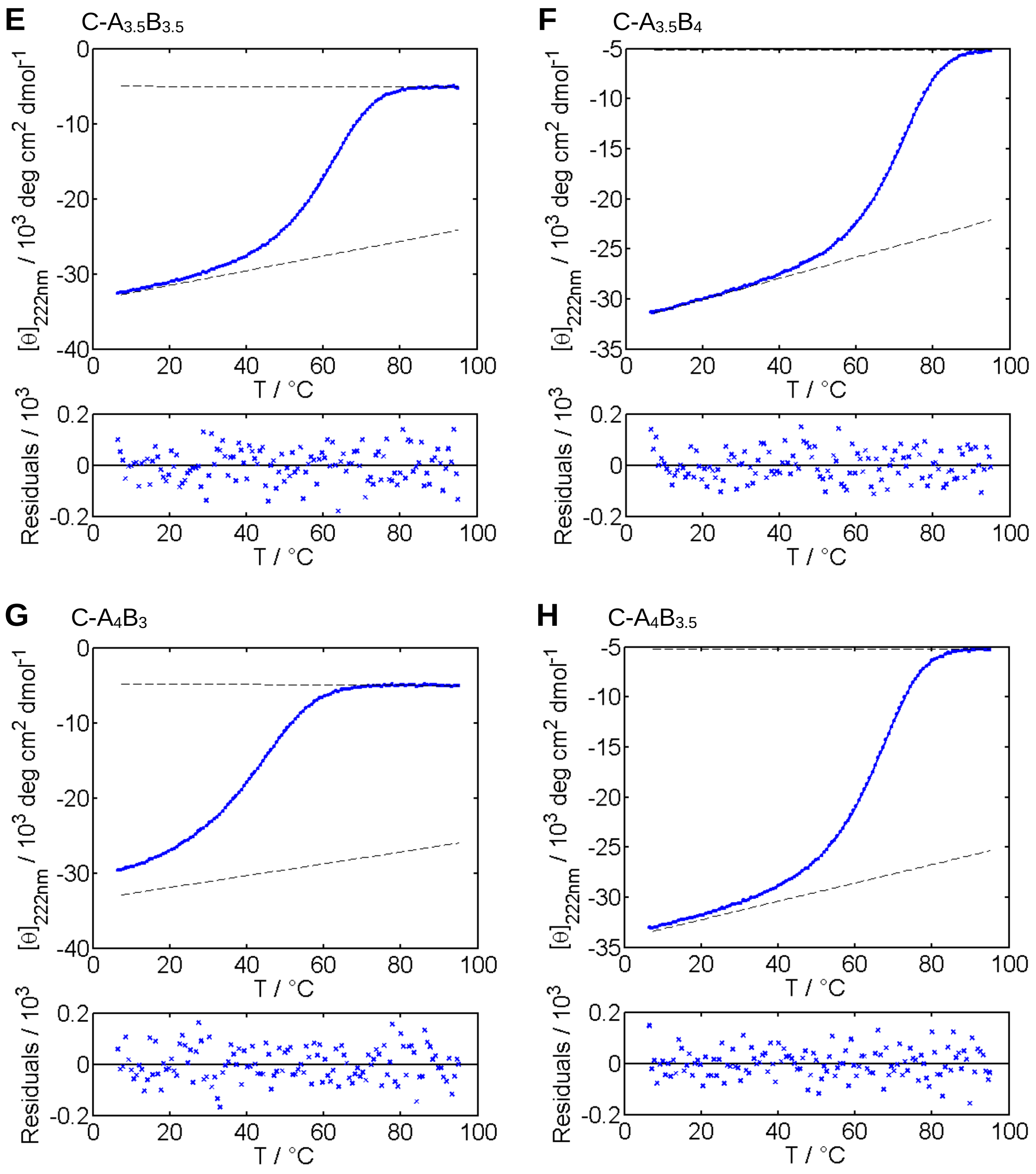

Figure S2. CD-thermal denaturation curves of the C-A_xB_y peptides fit to a two-state unfolding model (top) and residuals depicting the quality of the fit (bottom). A) C-A_3B_3, B) C-$A_3B_{3.5}$, C) C-A_3B_4, D) C-$A_{3.5}B_3$, E) C-$A_{3.5}B_{3.5}$, F) C-$A_{3.5}B_4$, G) C-A_4B_3, H) C-$A_4B_{3.5}$. Thermal denaturation curves were recorded at 50 μM coiled-coil concentration (A_4B_4: 25 μM coiled-coil concentration) in PBS buffer. Data were processed using the FitDis! analysis software.[1]

3. CD-titration experiments

A N-A_3B_3 ↑↓ $A_{3.5}$

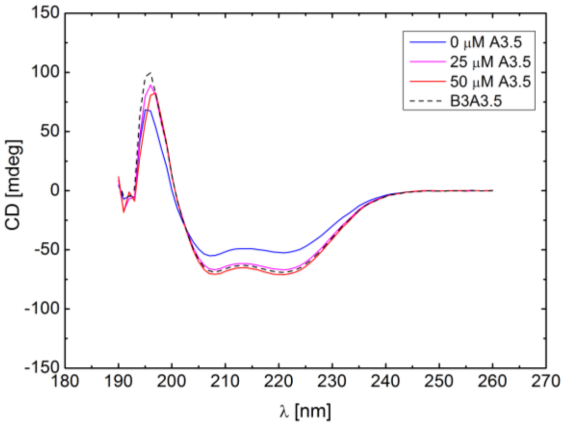

B N-$A_{3.5}B_3$ ↑↓ A_3

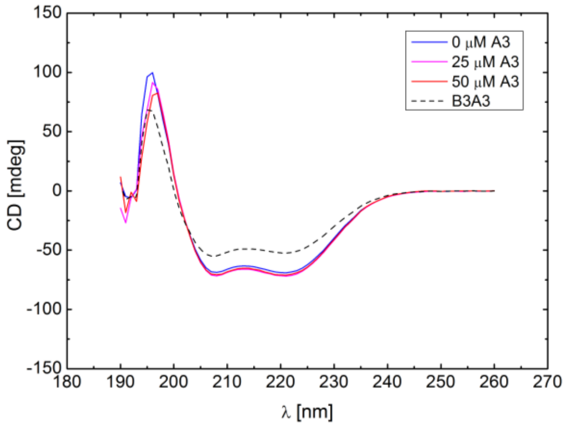

C N-A_3 B_3 ↑↓ A_4

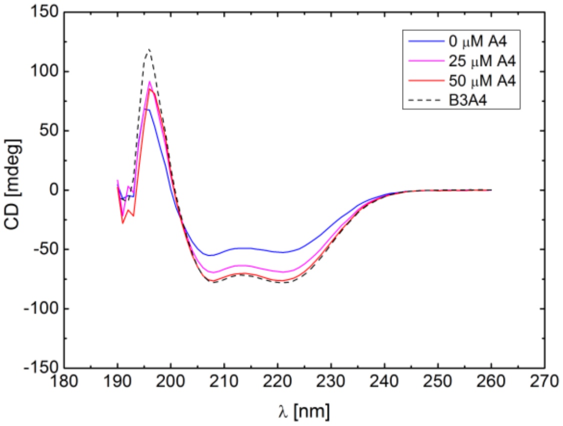

D N-A_4B_3 ↑↓ A_3

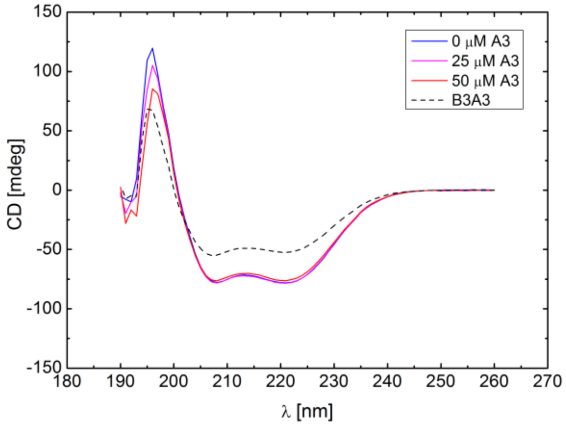

E N-A_3B_3 ↑↓ $B_{3.5}$

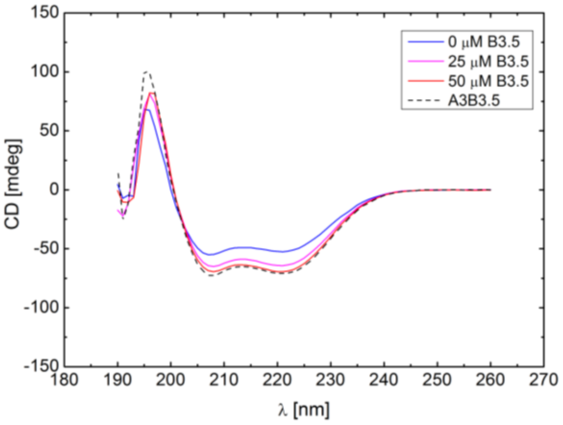

F N-$A_3B_{3.5}$ ↑↓ B_3

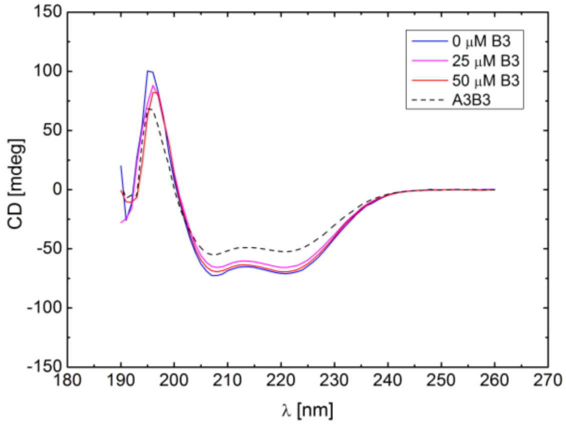

G N-A_3B_3 ↿⇂ B_4

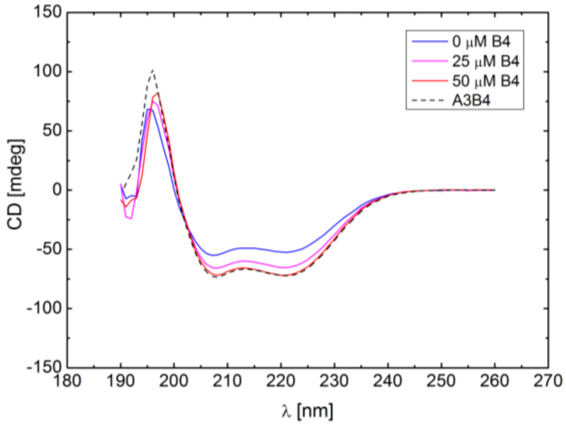

H N-A_3B_4 ↿⇂ B_3

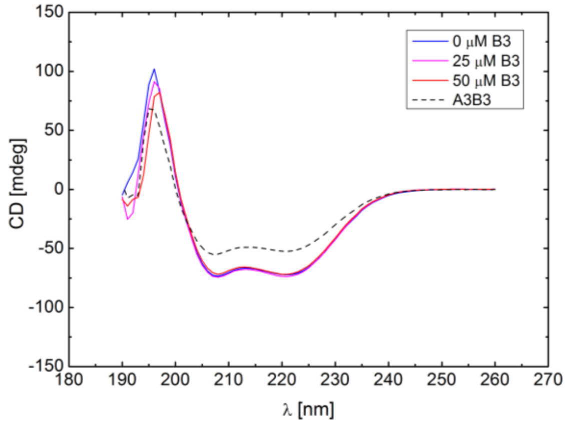

I N-$A_3B_{3.5}$ ↿⇂ $A_{3.5}$

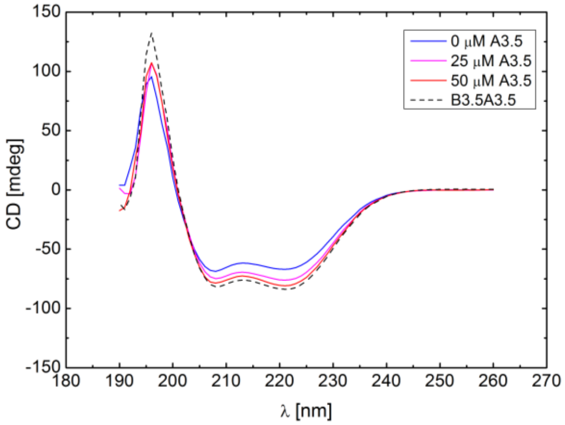

J N-$A_{3.5}$ $B_{3.5}$ ↿⇂ A_3

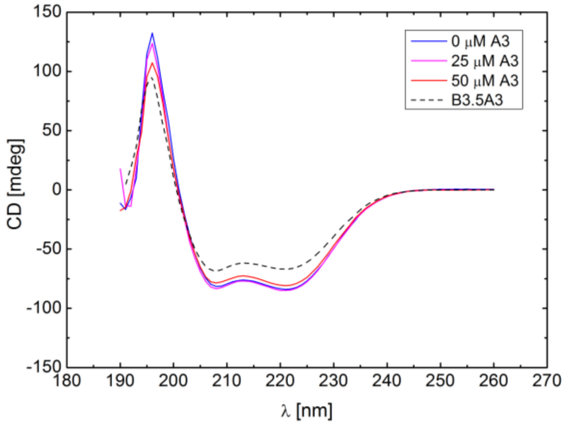

K N-$A_3B_{3.5}$ ↿⇂ A_4

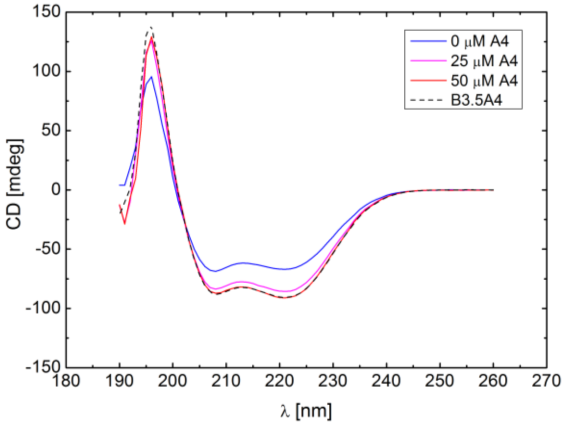

L N-$A_4B_{3.5}$ ↿⇂ A_3

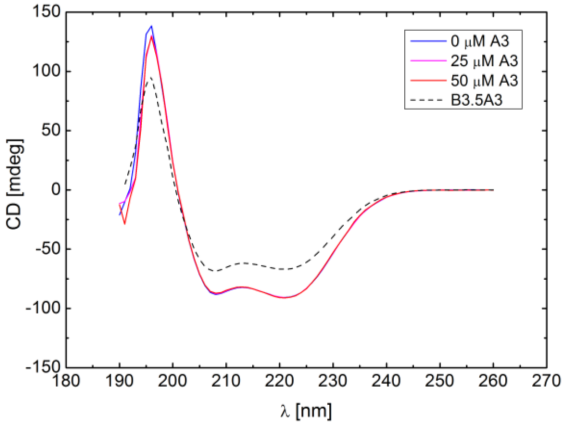

M N-$A_3B_{3.5}$ ↿⇂ B_4

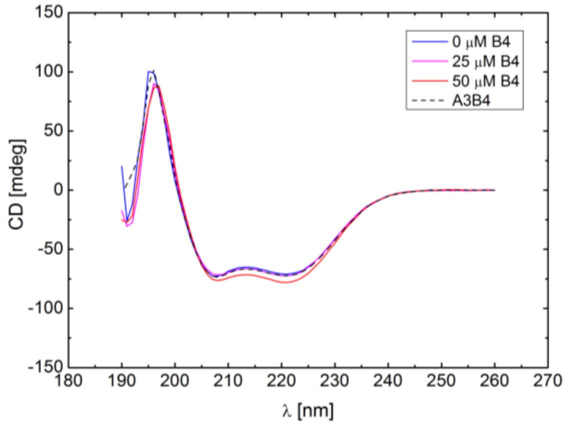

N N-A_3B_4 ↿⇂ $B_{3.5}$

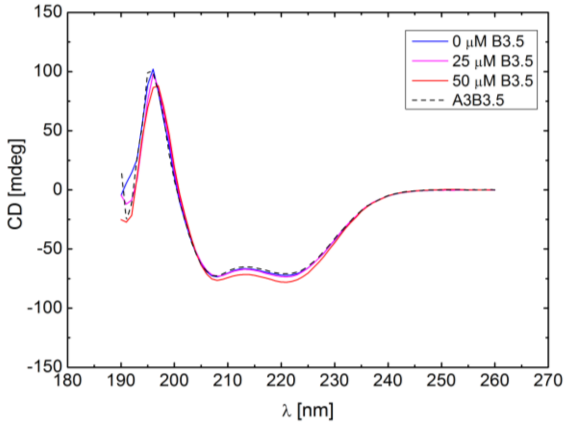

O N-A_3B_4 ↿⇂ $A_{3.5}$

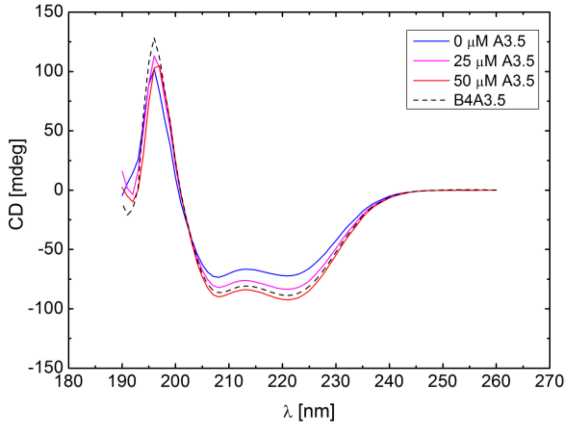

P N-$A_{3.5}B_4$ ↿⇂ A_3

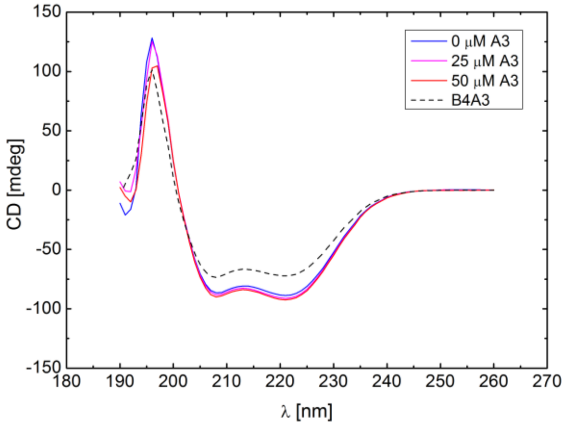

Q N-A_3B_4 ↿⇂ A_4

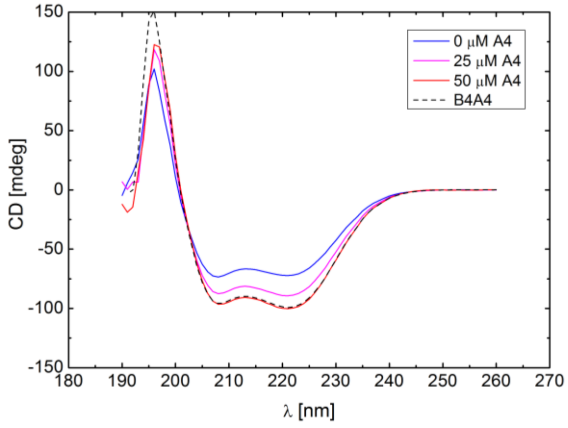

R N-A_4B_4 ↿⇂ A_3

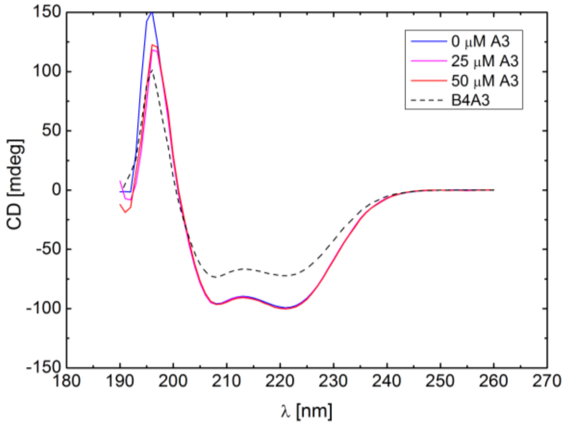

S N-$A_{3.5}B_3$ $\uparrow\downarrow$ A_4

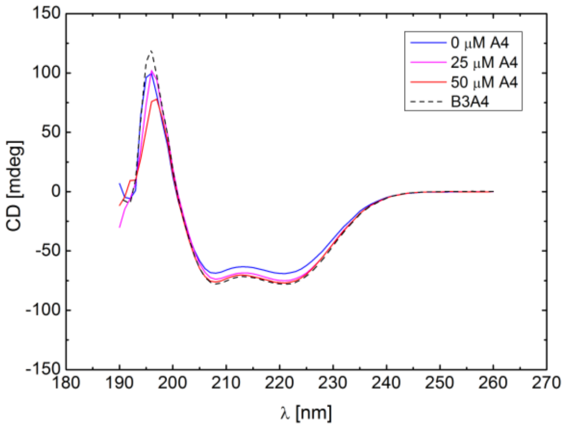

T N-A_4B_3 $\uparrow\downarrow$ $A_{3.5}$

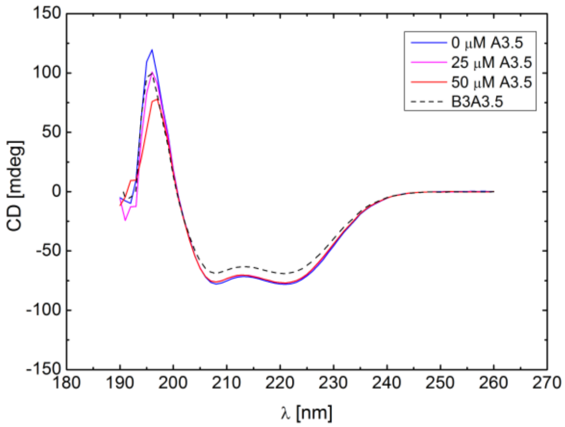

U N-$A_{3.5}B_3$ $\uparrow\downarrow$ $B_{3.5}$

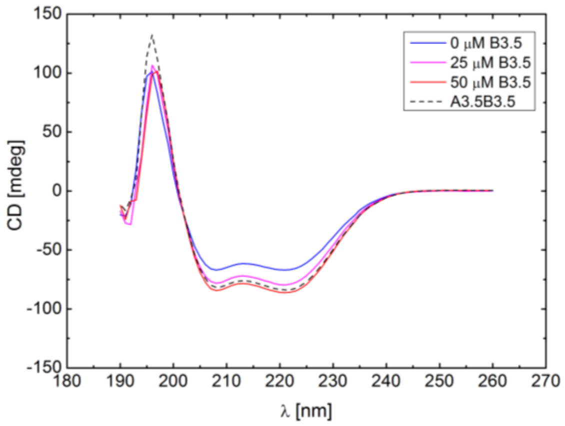

V N-$A_{3.5}B_{3.5}$ $\uparrow\downarrow$ B_3

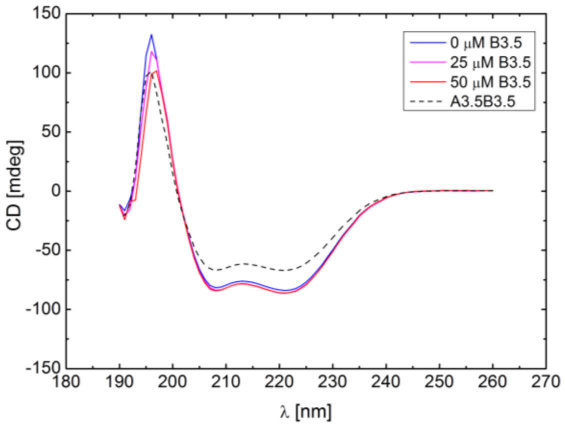

W N-$A_{3.5}B_3$ $\uparrow\downarrow$ B_4

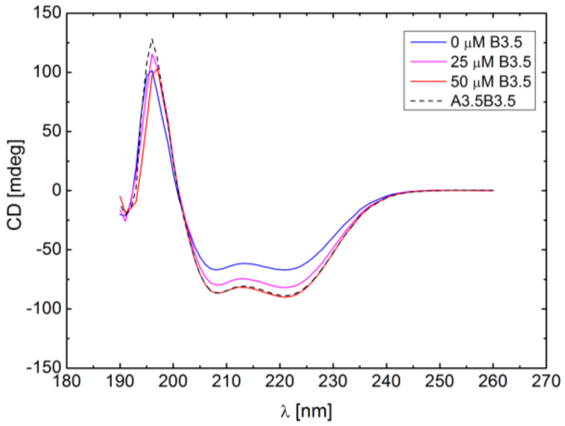

X N-$A_{3.5}B_4$ $\uparrow\downarrow$ B_3

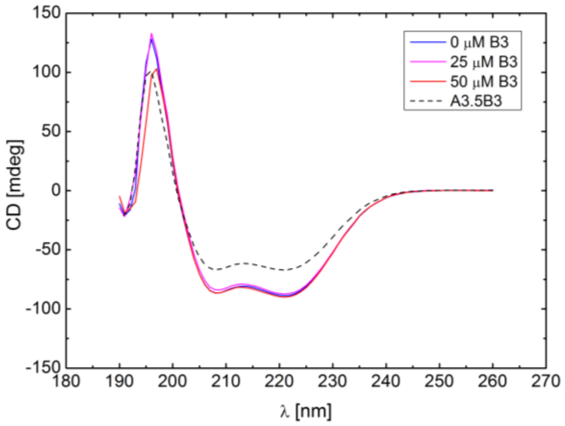

Y N-$A_{3.5}B_{3.5}$ ⇅ A_4

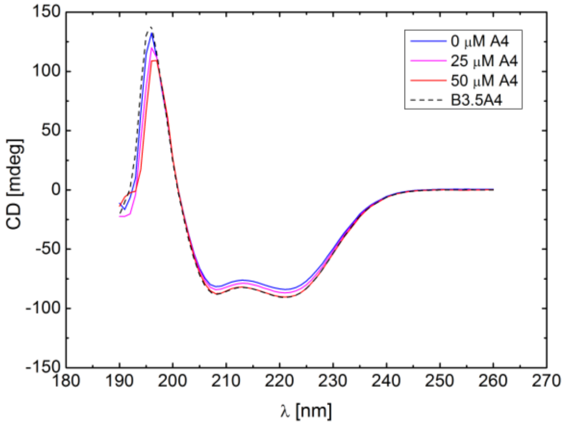

Z N-$A_4B_{3.5}$ ⇅ $A_{3.5}$

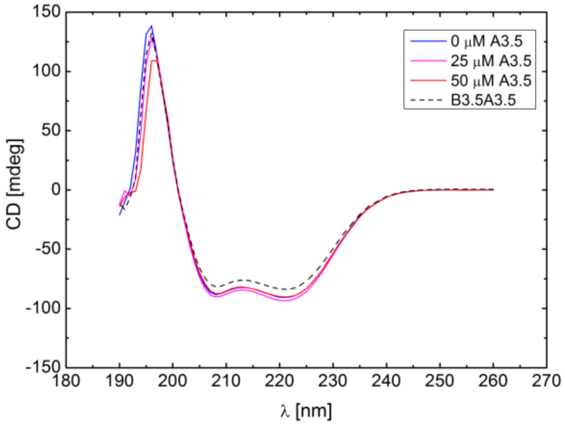

Aa N-$A_{3.5}B_{3.5}$ ⇅ B_4

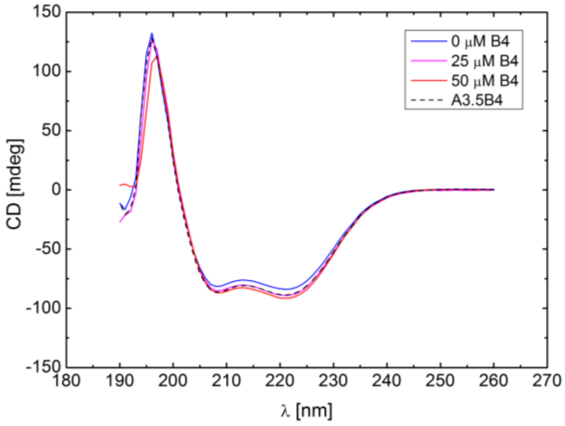

Bb N-$A_{3.5}B_4$ ⇅ $B_{3.5}$

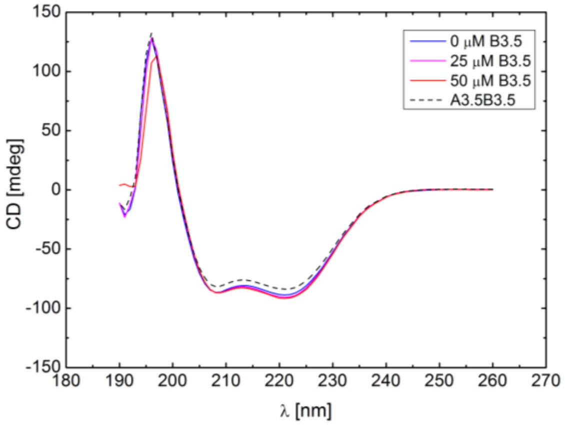

Cc N-$A_{3.5}B_4$ ⇅ A_4

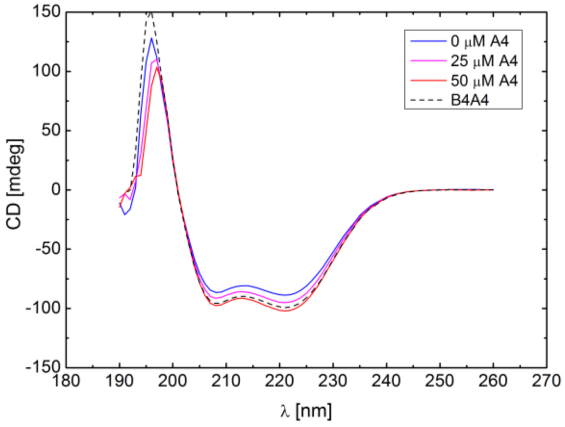

Dd N-A_4B_4 ⇅ $A_{3.5}$

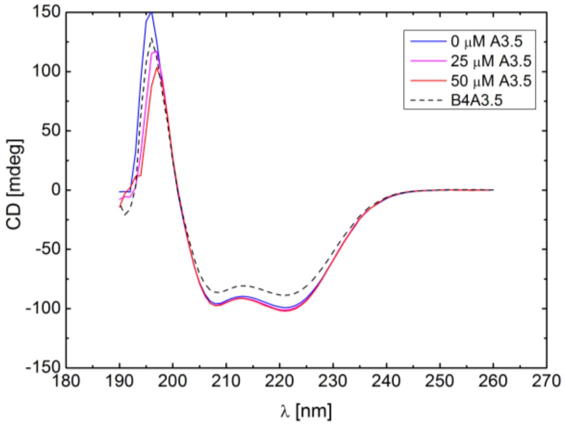

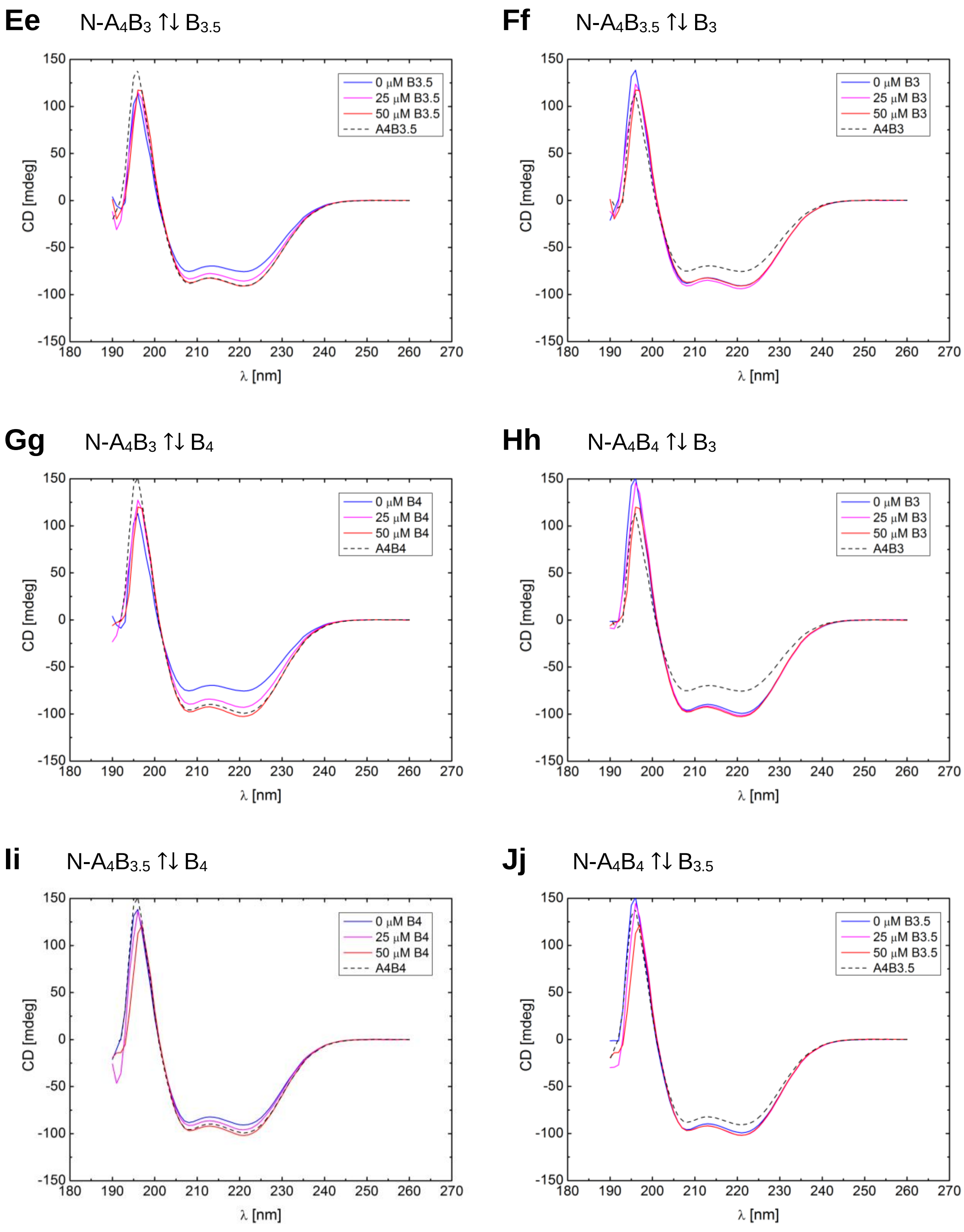

Figure S3. CD spectra of CD-titration experiments using the *N*-terminal truncated set of heterodimeric coiled coils. The most stable heterodimeric coiled coil is predominantly formed in solution.

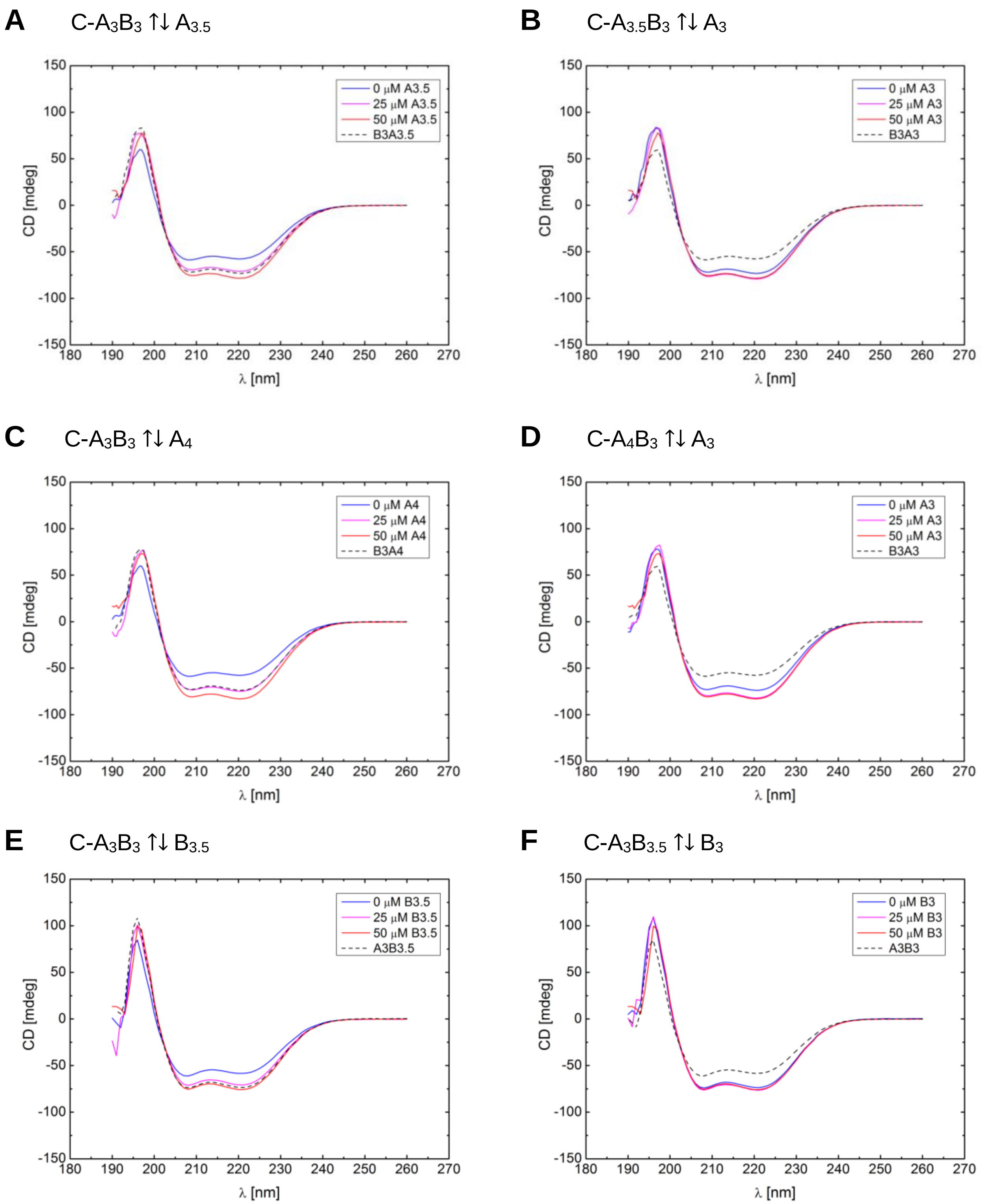
A
C-A3B3 ↑↓ A3.5
0 μM A3.5
25 μM A3.5
50 μM A3.5
B3A3.5
B
C-A3.5B3 ↑↓ A3
0 μM A3
25 μM A3
50 μM A3
B3A3
C
C-A3B3 ↑↓ A4
0 μM A4
25 μM A4
50 μM A4
B3A4
D
C-A4B3 ↑↓ A3
0 μM A3
25 μM A3
50 μM A3
B3A3
E
C-A3B3 ↑↓ B3.5
0 μM B3.5
25 μM B3.5
50 μM B3.5
A3B3.5
F
C-A3B3.5 ↑↓ B3
0 μM B3
25 μM B3
50 μM B3
A3B3
CD [mdeg]
λ [nm]

G C-A_3B_3 ⇅ B_4

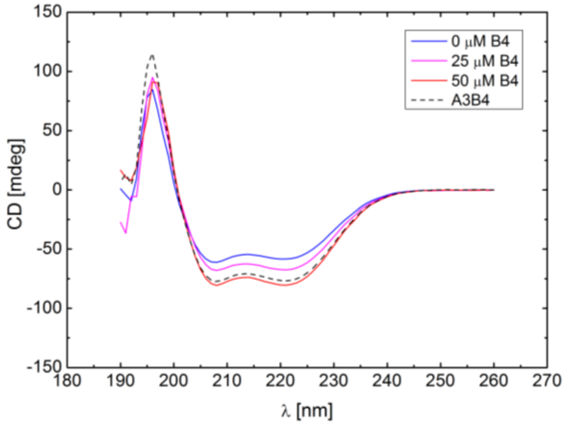

H C-A_3B_4 ⇅ B_3

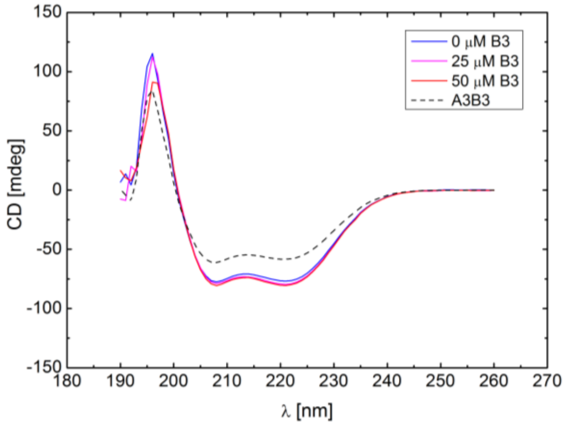

I C-$A_3B_{3.5}$ ⇅ $A_{3.5}$

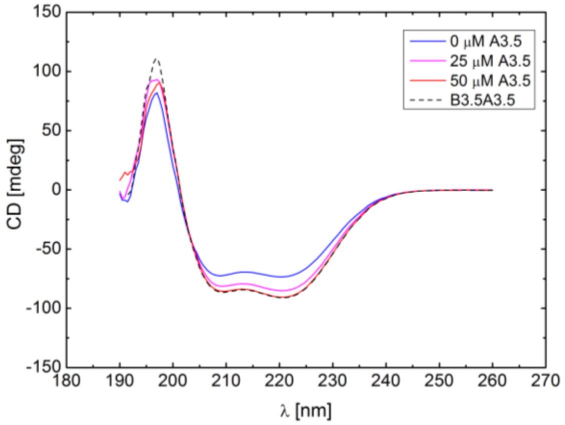

J C-$A_{3.5}B_{3.5}$ ⇅ A_3

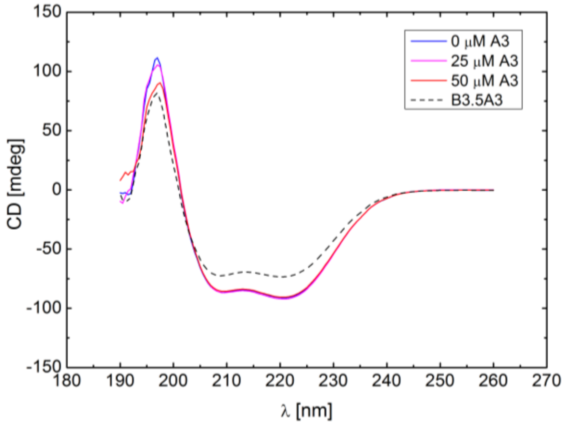

K C-$A_3B_{3.5}$ ⇅ A_4

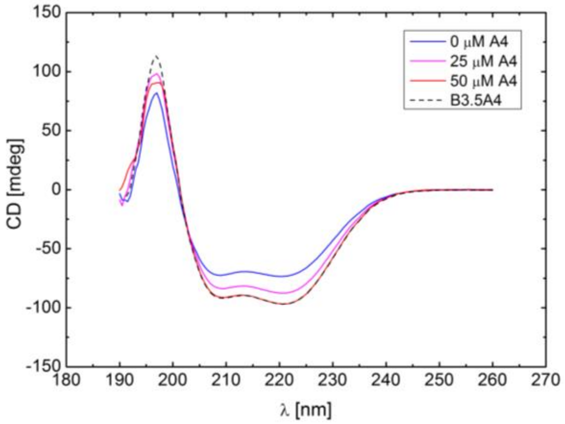

L C-$A_4B_{3.5}$ ⇅ A_3

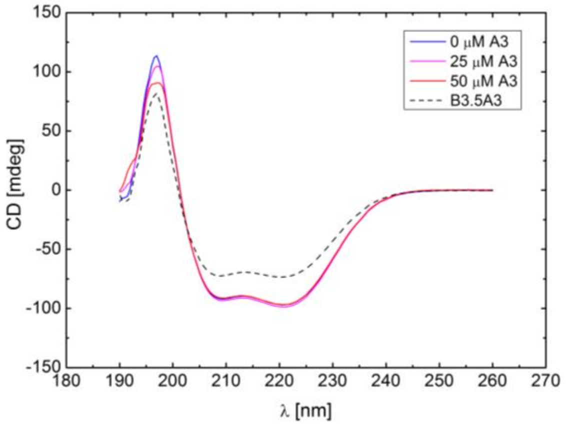

M C-$A_3B_{3.5}$ ↑↓ B_4

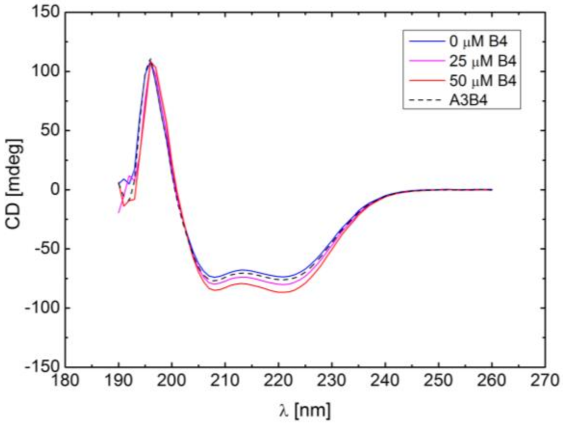

N C-A_3B_4 ↑↓ $B_{3.5}$

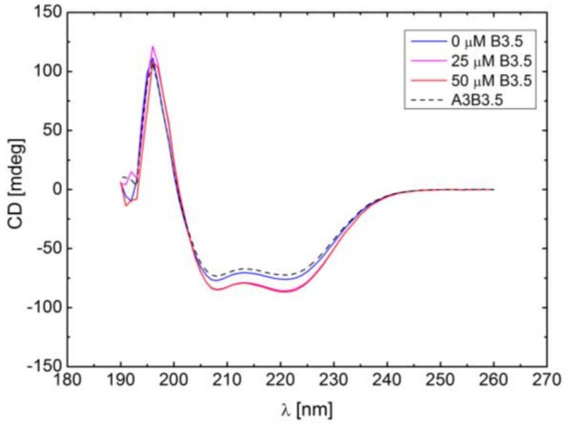

O C-A_3B_4 ↑↓ $A_{3.5}$

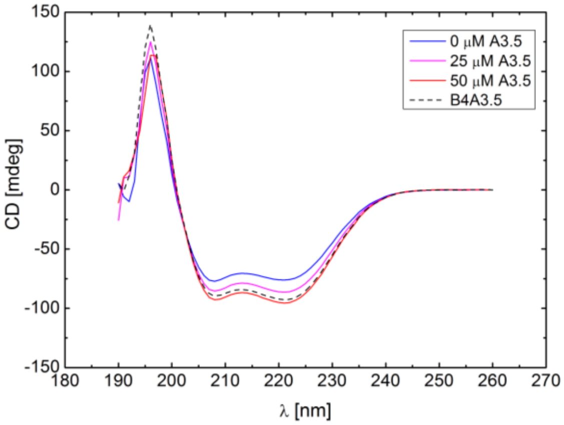

P C-$A_{3.5}B_4$ ↑↓ A_3

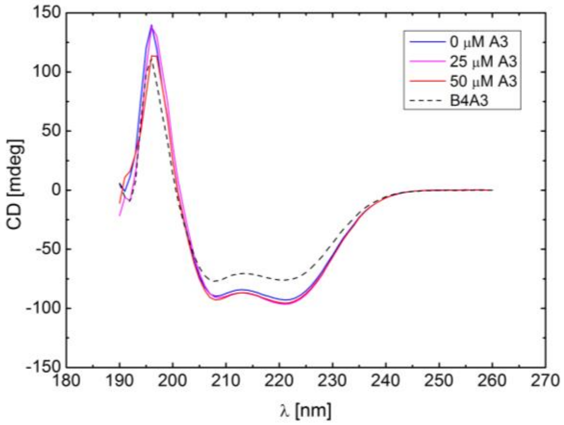

Q C-A_3B_4 ↑↓ A_4

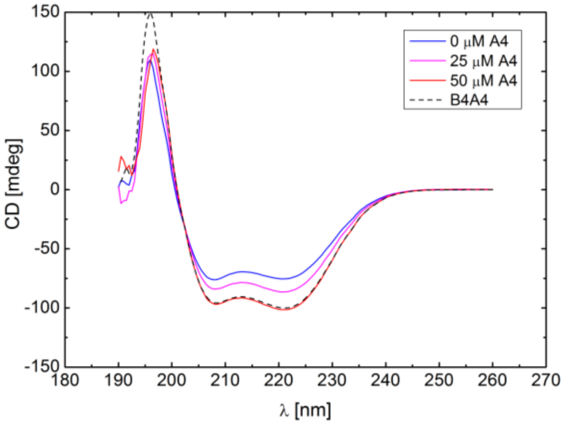

R C-A_4B_4 ↑↓ A_3

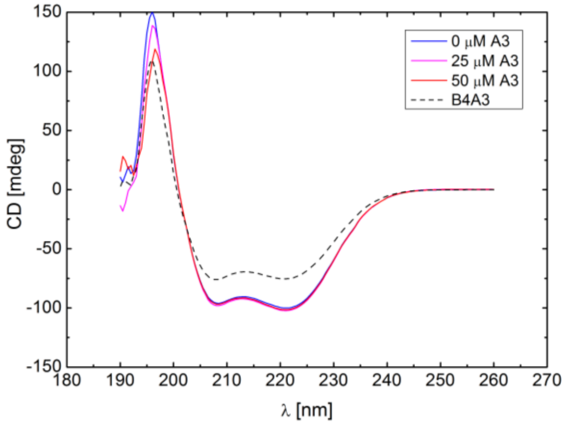

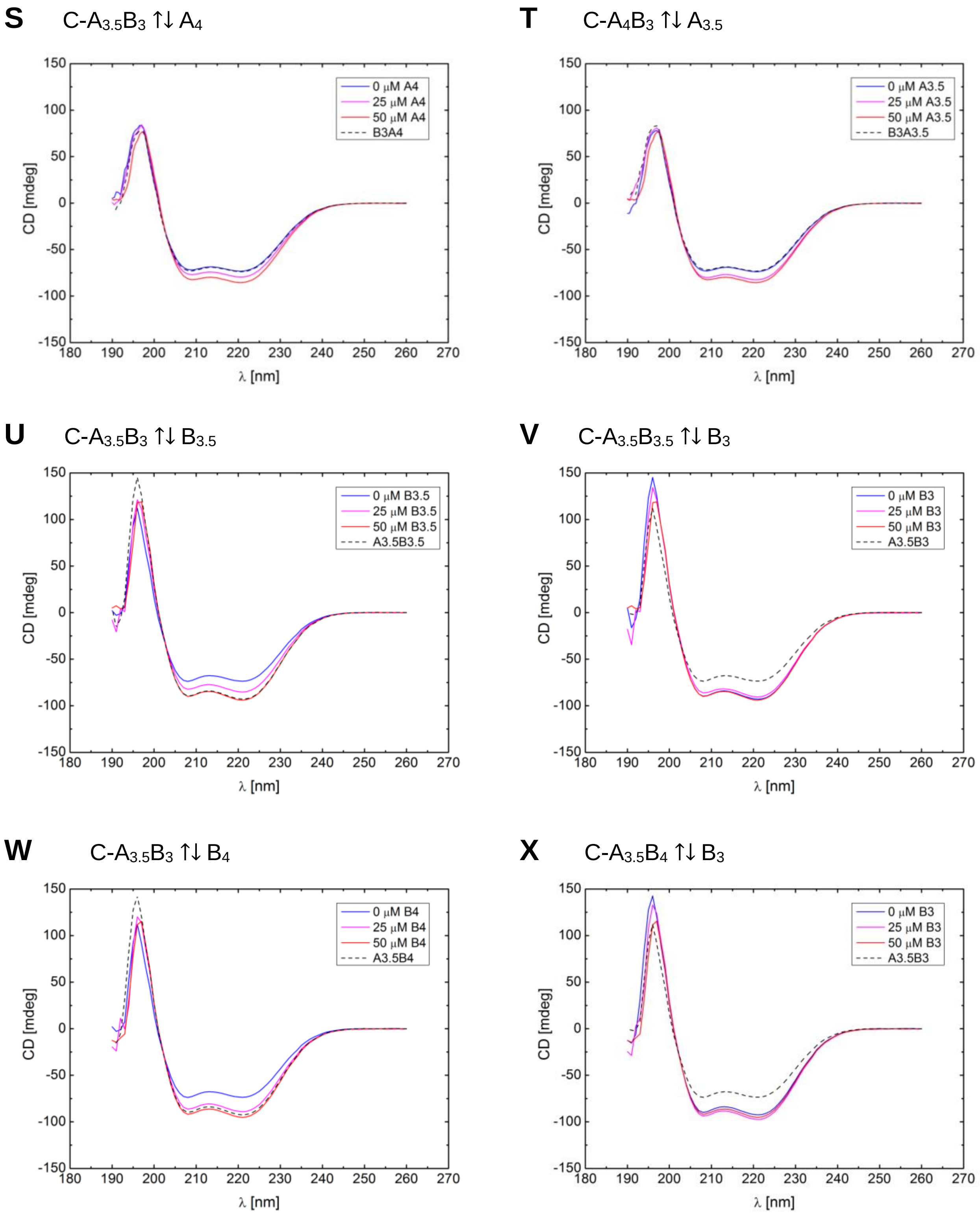
S
C-A3.5B3 ↑↓ A4
0 μM A4
25 μM A4
50 μM A4
B3A4
T
C-A4B3 ↑↓ A3.5
0 μM A3.5
25 μM A3.5
50 μM A3.5
B3A3.5
U
C-A3.5B3 ↑↓ B3.5
0 μM B3.5
25 μM B3.5
50 μM B3.5
A3.5B3.5
V
C-A3.5B3.5 ↑↓ B3
0 μM B3
25 μM B3
50 μM B3
A3.5B3
W
C-A3.5B3 ↑↓ B4
0 μM B4
25 μM B4
50 μM B4
A3.5B4
X
C-A3.5B4 ↑↓ B3
0 μM B3
25 μM B3
50 μM B3
A3.5B3
CD [mdeg]
λ [nm]

Y C-$A_{3.5}B_{3.5}$ ↑↓ A_4

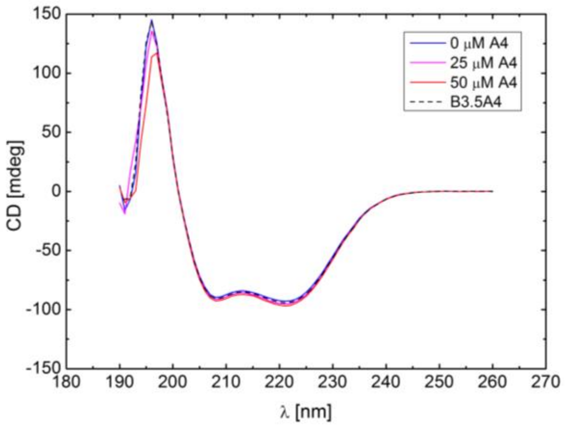

Z C-$B_{3.5}A_4$ ↑↓ $A_{3.5}$

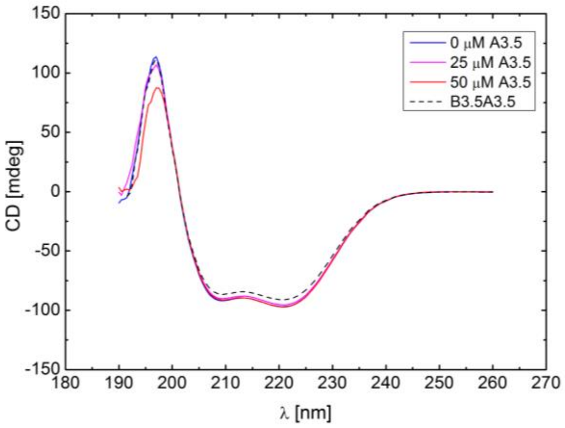

Aa C-$A_{3.5}B_{3.5}$ ↑↓ B_4

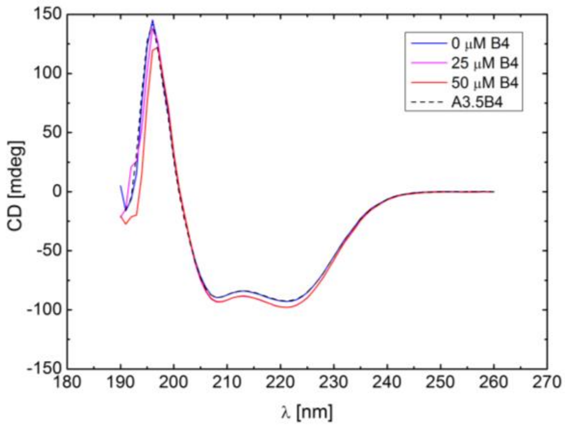

Bb C-$A_{3.5}B_4$ ↑↓ $B_{3.5}$

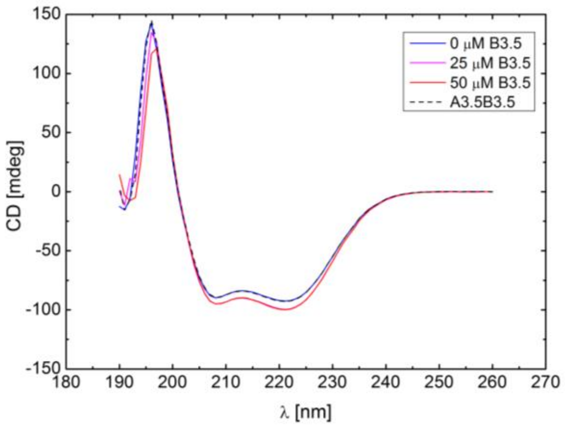

Cc C-$A_{3.5}B_4$ ↑↓ A_4

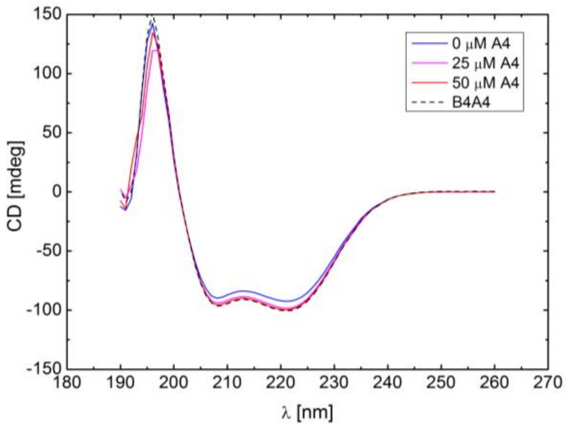

Dd C-A_4B_4 ↑↓ $A_{3.5}$

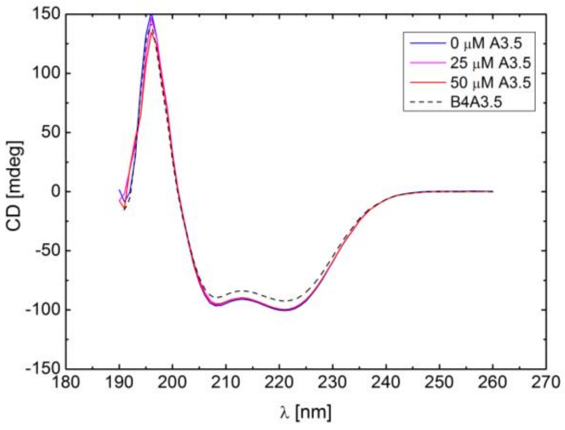

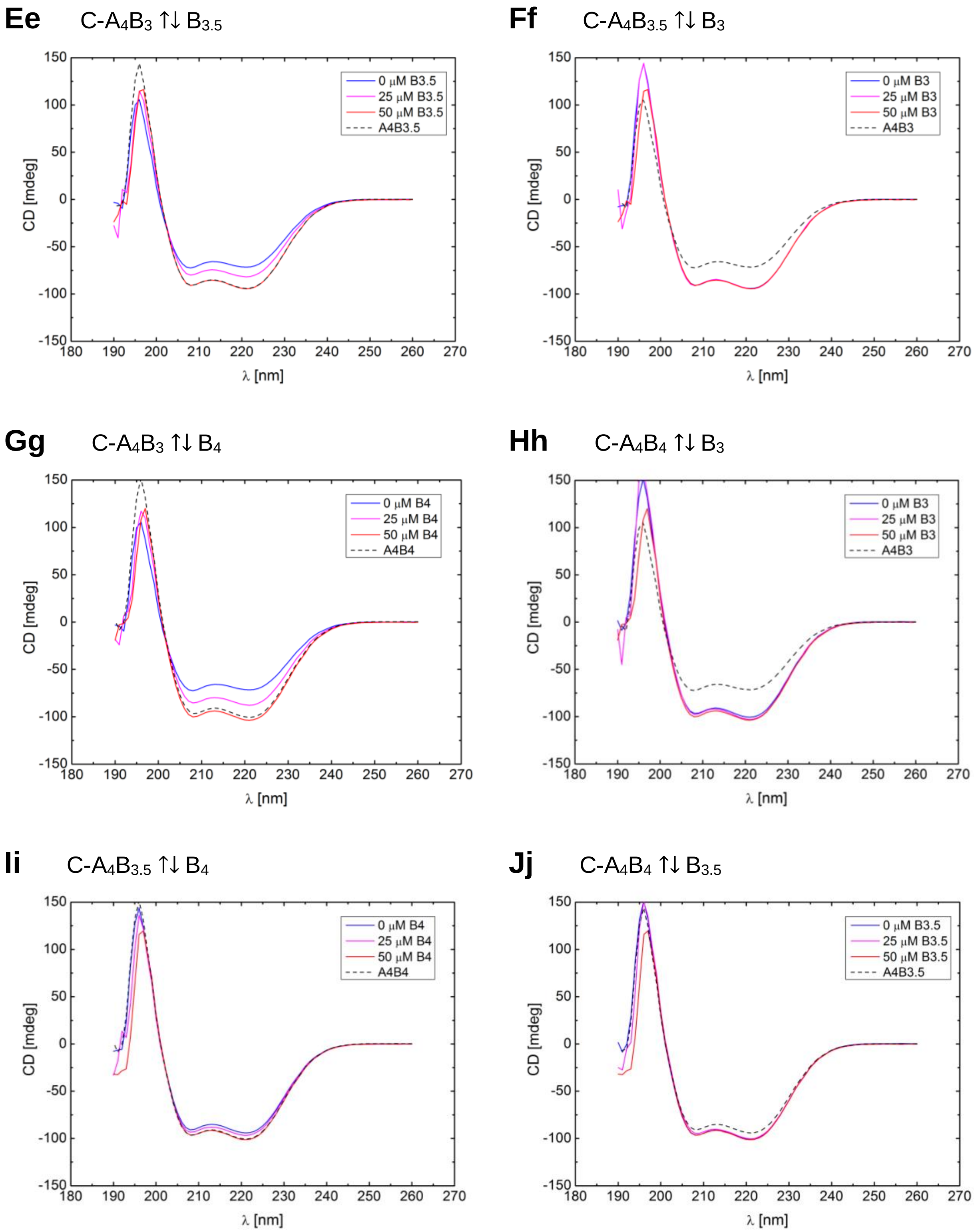

Figure S4. CD spectra of CD-titration experiments experiments using the *C*-terminal truncated set of heterodimeric coiled coils. The most stable heterodimeric coiled coil is predominantly formed in solution.

4. FRET-based strand-displacement assay

Time-resolved measurement of strand displacement monitored by FRET

To measure strand-displacement kinetics, A strands with intrinsically fluorescent Trp and Dansyl-labelled B-strands were used as FRET-donor-acceptor pairs (λ_{ex} = 270 nm, λ_{em} = 540 nm, Table S1). Labelled AB pairs were equilibrated at 15 µM concentration in PBS at 20 °C for 1 h and then at 4 °C for another 15 h in *Microfluor 1* 96 well plates, which were treated with 300 µL per well *Roche® Blocking reagent* (1 mg/mL) for 1 h and then washed with PBS (3x 100 µL) prior to use. We used a *Clariostar* platereader form *BMG Labtech* equipped with a 1 mL dispenser to record decrease of the FRET signal as a result of strand exchange. Strand displacement was performed by injecting equimolar amounts of non-labeled A and B strands (competitor peptide P_{comp}), respectively. First, maximum fluorescence (F_{max}) over 50 s was measured. Then, 1.5 µL of 1 mM P_{comp} were added and strand displacement was followed over 60 s at 540 nm. To obtain minimum fluorescence (F_{min}), another 5 µL of P_{comp} were added. Exemplarily, in Figure S5 time-resolved decrease of FRET signal in N-A_3B_4 upon the addition of non-labelled A_4 is shown. The obtained data was baseline-corrected by F_{min}, normalized to F_{max} and fitted to a single exponential decay (equation 1, Figures S6-S7).

$$\boldsymbol{F = F_{eq} + F_0 e^{-k_{obs}/t}} \quad (1)$$

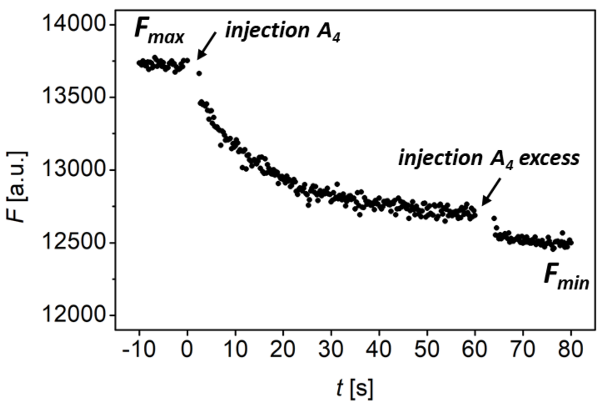

Figure S5. Strand displacement in N-A_3B_4 by A_4. Labelled N-A_3 and B_4 were equilibrated at 15 µM peptide concentration in PBS. Change in Dansyl fluorescence was recorded at 540 nm. After 50 s equimolar amounts of non-labelled A_4 were added and fluorescence decrease was monitored for 60 s. To determine F_{min}, another 5 µL non-labelled A_4 were added.

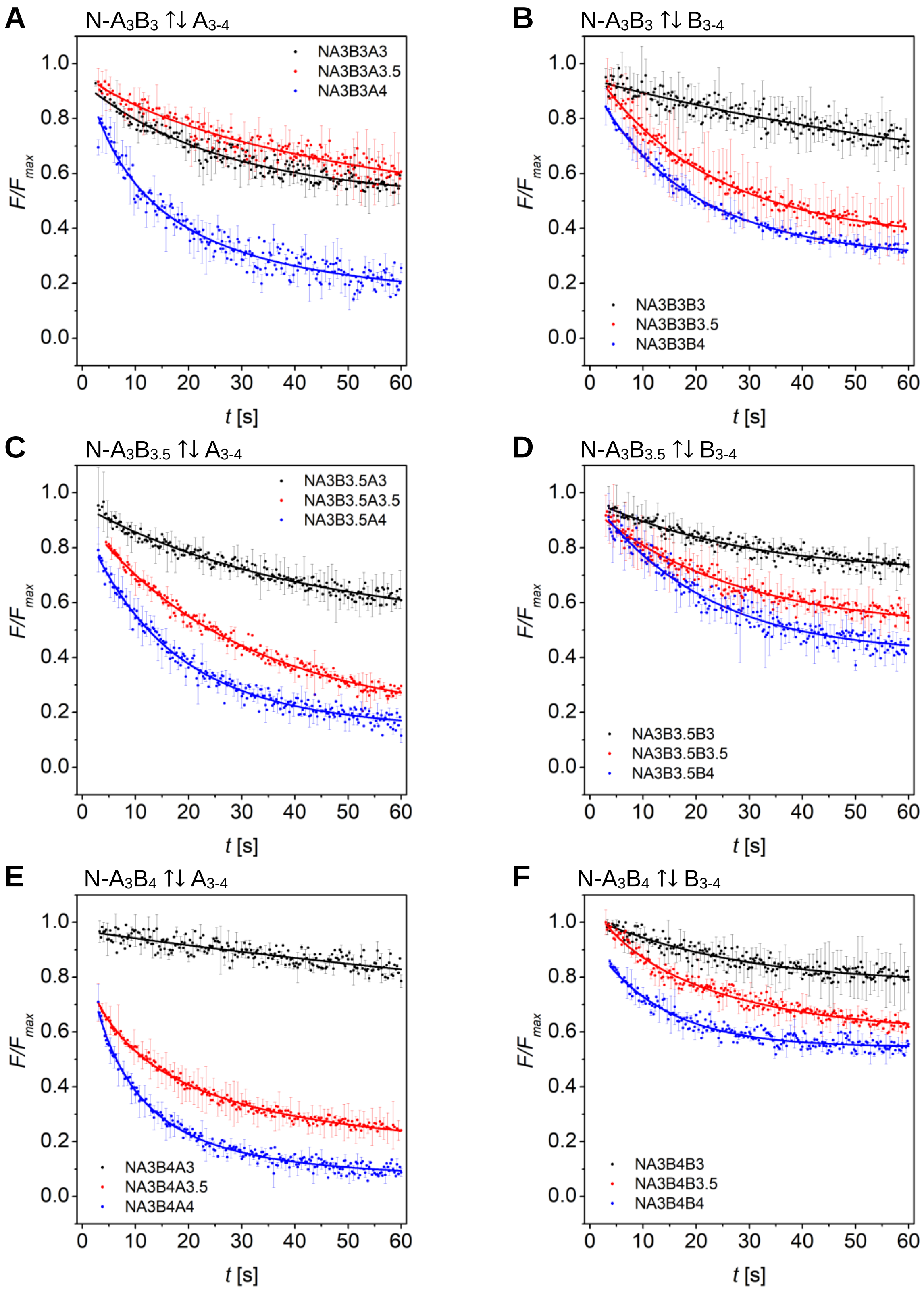

A
N-A3B3 ↿⇂ A3-4
NA3B3A3
NA3B3A3.5
NA3B3A4
B
N-A3B3 ↿⇂ B3-4
NA3B3B3
NA3B3B3.5
NA3B3B4
C
N-A3B3.5 ↿⇂ A3-4
NA3B3.5A3
NA3B3.5A3.5
NA3B3.5A4
D
N-A3B3.5 ↿⇂ B3-4
NA3B3.5B3
NA3B3.5B3.5
NA3B3.5B4
E
N-A3B4 ↿⇂ A3-4
NA3B4A3
NA3B4A3.5
NA3B4A4
F
N-A3B4 ↿⇂ B3-4
NA3B4B3
NA3B4B3.5
NA3B4B4
F/Fmax
t [s]

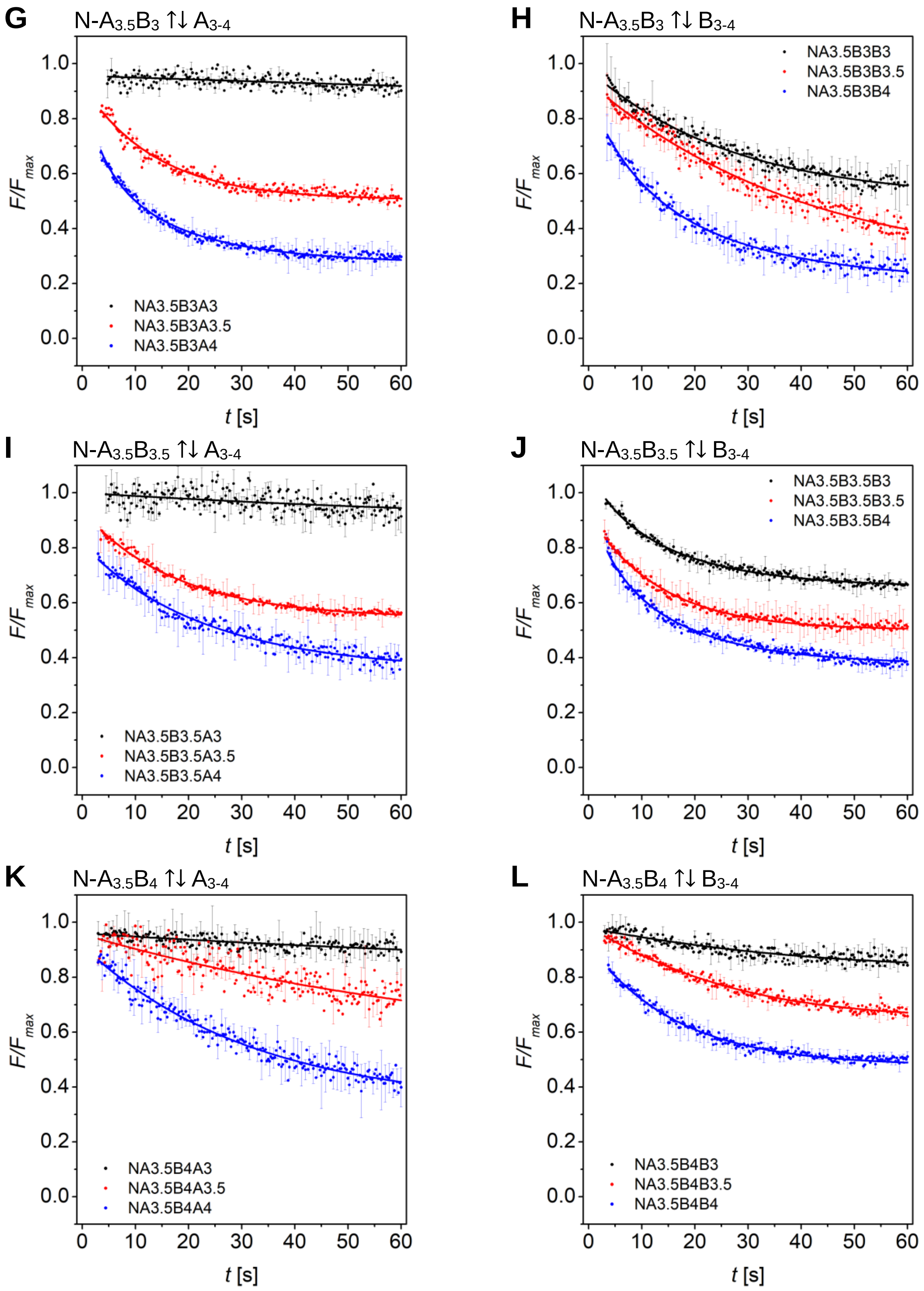
G
N-A3.5B3 ↑↓ A3-4
NA3.5B3A3
NA3.5B3A3.5
NA3.5B3A4
H
N-A3.5B3 ↑↓ B3-4
NA3.5B3B3
NA3.5B3B3.5
NA3.5B3B4
I
N-A3.5B3.5 ↑↓ A3-4
NA3.5B3.5A3
NA3.5B3.5A3.5
NA3.5B3.5A4
J
N-A3.5B3.5 ↑↓ B3-4
NA3.5B3.5B3
NA3.5B3.5B3.5
NA3.5B3.5B4
K
N-A3.5B4 ↑↓ A3-4
NA3.5B4A3
NA3.5B4A3.5
NA3.5B4A4
L
N-A3.5B4 ↑↓ B3-4
NA3.5B4B3
NA3.5B4B3.5
NA3.5B4B4
F/Fmax
t [s]

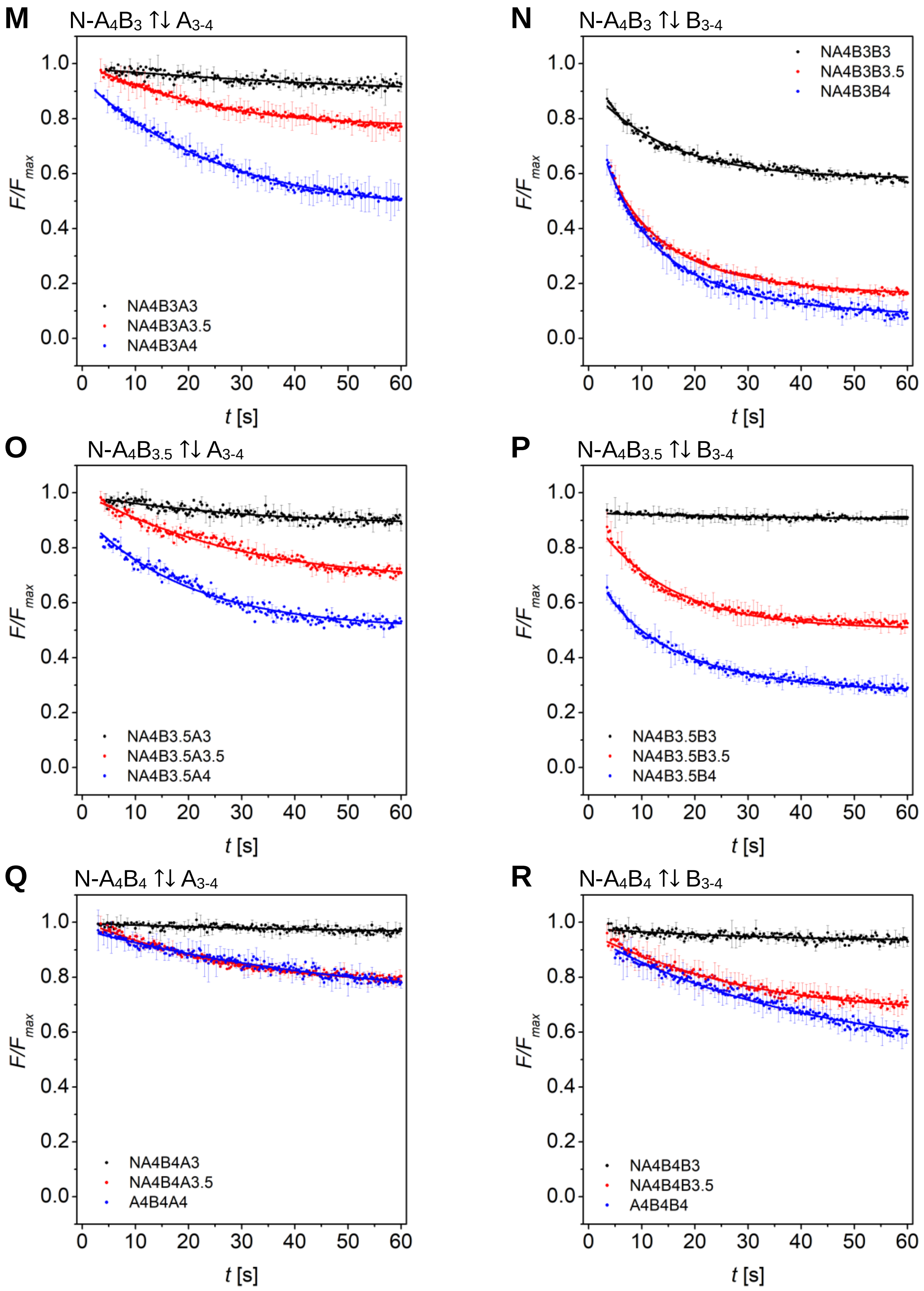

Figure S6. Normalized time-resolved fluorescence decrease upon strand displacement in N-A_xB_y peptides. Fluorescence data was recorded at Dansyl fluorescence at 540 nm (λ_{ex} = 270 nm). Data were baseline-corrected by F_{min} and normalized to F_{max}, respectively, and is the average of three measurements.

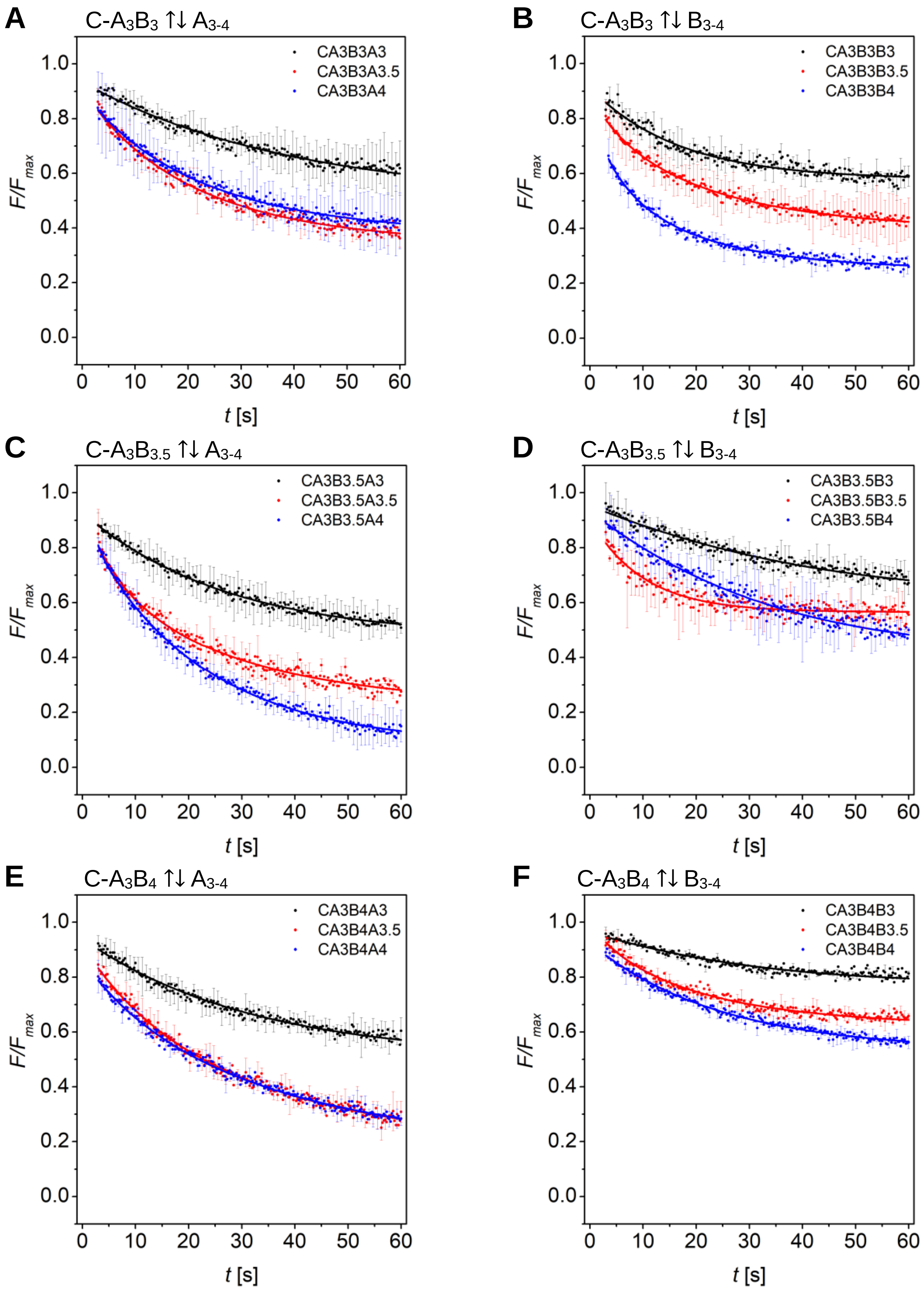
A
C-A3B3 ↿⇂ A3-4
CA3B3A3
CA3B3A3.5
CA3B3A4
B
C-A3B3 ↿⇂ B3-4
CA3B3B3
CA3B3B3.5
CA3B3B4
C
C-A3B3.5 ↿⇂ A3-4
CA3B3.5A3
CA3B3.5A3.5
CA3B3.5A4
D
C-A3B3.5 ↿⇂ B3-4
CA3B3.5B3
CA3B3.5B3.5
CA3B3.5B4
E
C-A3B4 ↿⇂ A3-4
CA3B4A3
CA3B4A3.5
CA3B4A4
F
C-A3B4 ↿⇂ B3-4
CA3B4B3
CA3B4B3.5
CA3B4B4
F/Fmax
t [s]

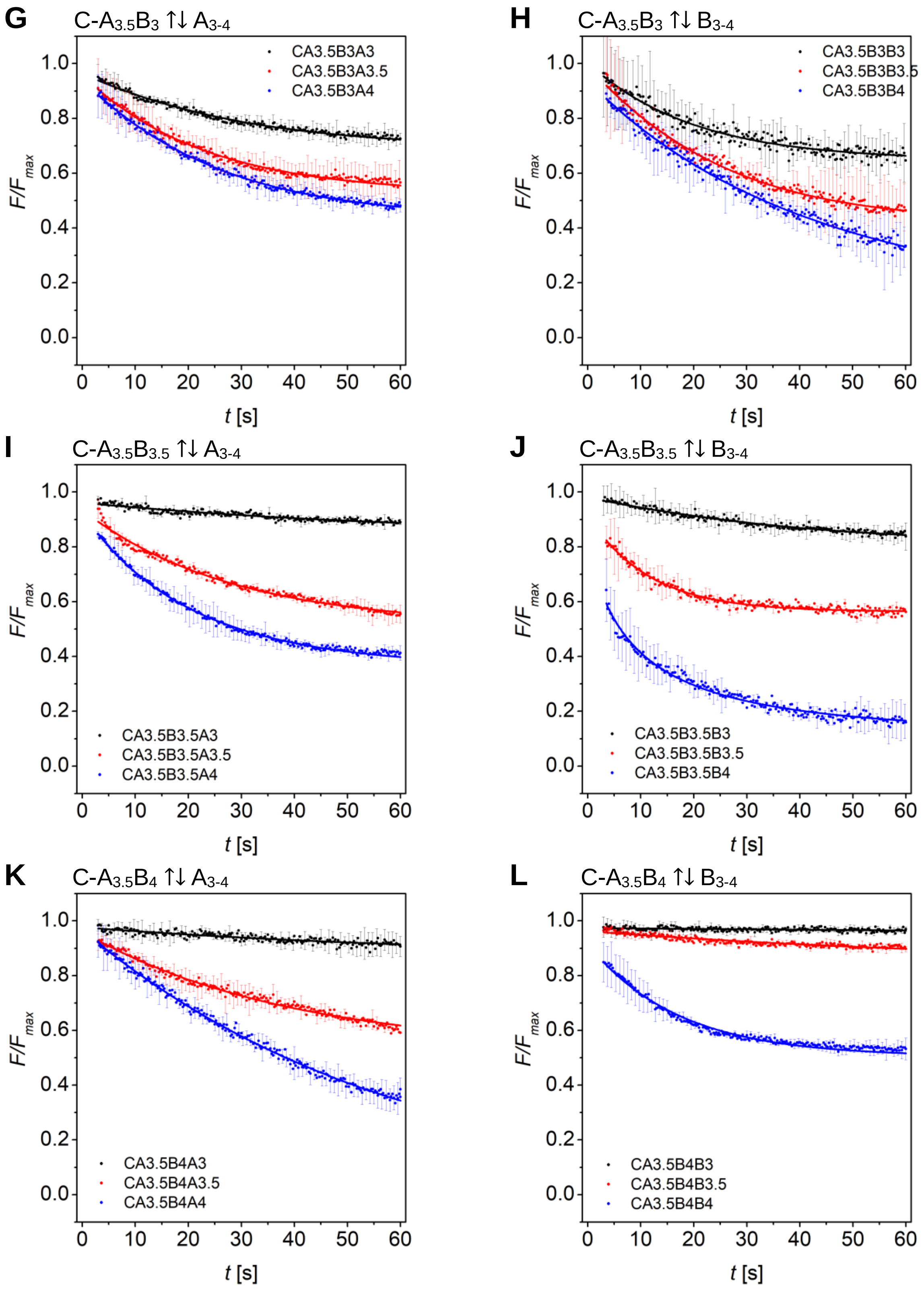
G
C-A3.5B3 ↑↓ A3-4
CA3.5B3A3
CA3.5B3A3.5
CA3.5B3A4
H
C-A3.5B3 ↑↓ B3-4
CA3.5B3B3
CA3.5B3B3.5
CA3.5B3B4
I
C-A3.5B3.5 ↑↓ A3-4
CA3.5B3.5A3
CA3.5B3.5A3.5
CA3.5B3.5A4
J
C-A3.5B3.5 ↑↓ B3-4
CA3.5B3.5B3
CA3.5B3.5B3.5
CA3.5B3.5B4
K
C-A3.5B4 ↑↓ A3-4
CA3.5B4A3
CA3.5B4A3.5
CA3.5B4A4
L
C-A3.5B4 ↑↓ B3-4
CA3.5B4B3
CA3.5B4B3.5
CA3.5B4B4
F/Fmax
t [s]
0.0
0.2
0.4
0.6
0.8
1.0
0
10
20
30
40
50
60

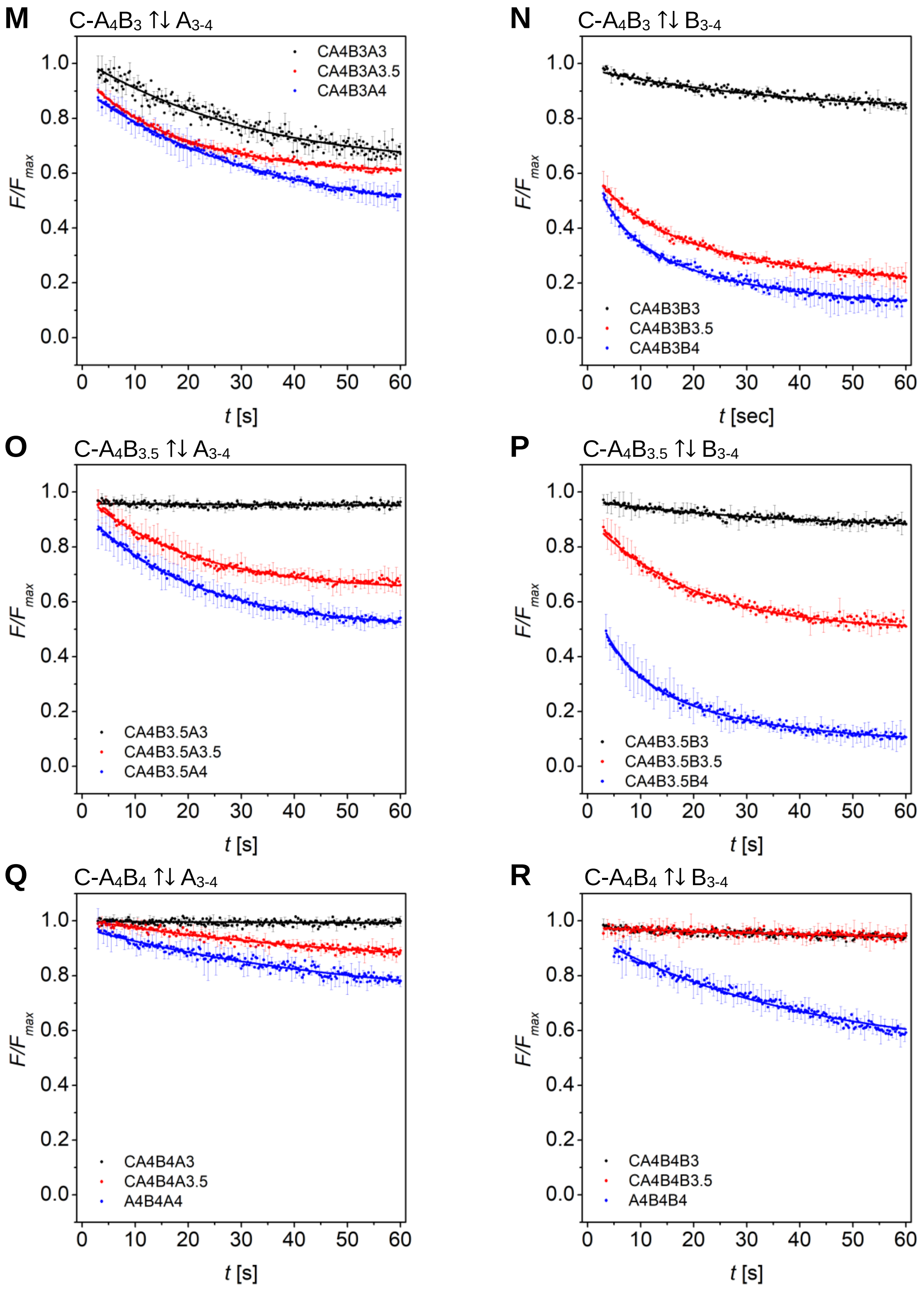

Figure S7. Normalized time-resolved fluorescence decrease upon strand displacement in C-A_xB_y peptides. Fluorescence data was recorded at Dansyl fluorescence at 540 nm (λ_{ex} = 270 nm). Data were baseline-corrected by F_{min} and normalized to F_{max}, respectively, and is the average of three measurements.

Table S5. Best-fit values of observed rate constants (k_{obs}) and half-lifes ($t_{1/2}$) of strand displacement in N-A_xB_y peptides. Values were obtained from a FRET-based kinetic assay and data fitting by a single exponential decay model (k_{obs}, $t_{1/2}$), and by a competitive binding model using DynaFit.[2,3] Overall affinities (k_1k_4/k_2k_3) match well with the ratios of the K_D values determined from CD thermal denaturation curves.

N-A_xB_y peptide		P_{comp}	k_{obs} [s^{-1}]	$t_{1/2}$ [s]
N-A_3	B_3	A_3	0.037 ± 0.003	17.3 ± 1.4
		$A_{3.5}$	0.025 ± 0.003	27.7 ± 3.3
		A_4	0.063 ± 0.003	11.0 ± 0.5
		B_3	0.010 ± 0.004	69.3 ± 27.7
		$B_{3.5}$	0.046 ± 0.002	15.1 ± 0.7
		B_4	0.055 ± 0.001	12.6 ± 0.2
	$B_{3.5}$	A_3	0.025 ± 0.002	27.7 ± 2.2
		$A_{3.5}$	0.032 ± 0.001	21.7 ± 0.5
		A_4	0.058 ± 0.002	12.0 ± 0.4
		B_3	0.033 ± 0.003	21.0 ± 1.9
		$B_{3.5}$	0.045 ± 0.002	15.4 ± 0.7
		B_4	0.042± 0.002	16.5 ± 0.8
	B_4	A_3	n.a.[a]	n.a.[a]
		$A_{3.5}$	0.057 ± 0.001	12.2 ± 0.2
		A_4	0.085 ± 0.002	8.2 ± 0.2
		B_3	0.040 ± 0.004	17.3 ± 1.7
		$B_{3.5}$	0.048 ± 0.002	14.4 ± 0.6
		B_4	0.076 ± 0.004	9.1 ± 0.5
N-$A_{3.5}$	B_3	A_3	n.a.[a]	n.a.[a]
		$A_{3.5}$	0.082 ± 0.003	8.5 ± 0.3
		A_4	0.087 ± 0.002	8.0 ± 0.2
		B_3	0.037 ± 0.002	18.7 ± 1.0
		$B_{3.5}$	0.029 ± 0.003	23.9 ± 2.5
		B_4	0.064 ± 0.002	10.8 ± 0.3
	$B_{3.5}$	A_3	n.a.[a]	n.a.[a]
		$A_{3.5}$	0.056 ± 0.001	12.4 ± 0.2
		A_4	0.045 ± 0.002	15.4 ± 0.7
		B_3	0.068 ± 0.002	10.2 ± 0.3
		$B_{3.5}$	0.085 ± 0.002	8.2 ± 0.2
		B_4	0.071 ± 0.002	9.8 ± 0.3
	B_4	A_3	n.a.[a]	n.a.[a]
		$A_{3.5}$	0.024 ± 0.005	28.9 ± 6.0
		A_4	0.033 ± 0.001	21.0 ± 0.6
		B_3	0.048 ± 0.005	14.4 ± 1.5
		$B_{3.5}$	0.040 ± 0.002	17.3 ± 0.9
		B_4	0.065 ± 0.001	10.7 ± 0.2
N-A_4	B_3	A_3	0.032 ± 0.008	21.7 ± 5.4
		$A_{3.5}$	0.033 ± 0.001	21.0 ± 0.6
		A_4	0.039 ± 0.001	17.8 ± 0.5
		B_3	0.068 ± 0.002	10.2 ± 0.3
		$B_{3.5}$	0.079 ± 0.001	8.8 ± 0.1
		B_4	0.080 ± 0.001	8.7 ± 0.1
	$B_{3.5}$	A_3	0.034 ± 0.006	20.4 ± 3.6
		$A_{3.5}$	0.027 ± 0.002	25.7 ± 1.9
		A_4	0.043 ± 0.002	16.1 ± 0.7
		B_3	n.a.[a]	n.a.[a]
		$B_{3.5}$	0.092 ± 0.001	7.5 ± 0.1
		B_4	0.072 ± 0.001	9.6 ± 0.1
	B_4	A_3	n.a.[a]	n.a.[a]
		$A_{3.5}$	0.042 ± 0.002	16.5 ± 0.8
		A_4	0.019 ± 0.002	36.5 ± 3.8
		B_3	n.a.[a]	n.a.[a]
		$B_{3.5}$	0.042 ± 0.002	16.5 ± 0.8
		B_4	0.012 ± 0.001	57.8 ± 4.8

[a]Data did not fit the single exponential model.

Table S6. Best-fit values of observed rate constants (k_{obs}) and half-lifes ($t_{1/2}$) of strand displacement in C-A_xB_y peptides. Values were obtained from a FRET-based kinetic assay and data fitting by a single exponential decay model (k_{obs}, $t_{1/2}$), and by a competitive binding model using DynaFit.[2,3] Overall affinities (k_1k_4/k_2k_3) match well with the ratios of the K_D values determined from CD thermal denaturation curves.

C-A_xB_y peptide		P_{comp}	k_{obs} [s^{-1}]	$t_{1/2}$ [s]
C-A_3	B_3	A_3	0.034 ± 0.002	20.4 ± 1.2
		$A_{3.5}$	0.050 ± 0.002	13.9 ± 0.6
		A_4	0.044 ± 0.001	15.8 ± 0.4
		B_3	0.059 ± 0.003	11.7 ± 0.6
		$B_{3.5}$	0.057 ± 0.002	12.2 ± 0.4
		B_4	0.078 ± 0.002	8.9 ± 0.2
	$B_{3.5}$	A_3	0.037 ± 0.001	18.7 ± 0.5
		$A_{3.5}$	0.055 ± 0.002	12.6 ± 0.5
		A_4	0.048 ± 0.001	14.4 ± 0.3
		B_3	0.033 ± 0.002	21.0 ± 1.3
		$B_{3.5}$	0.102 ± 0.006	6.8 ± 0.4
		B_4	0.031 ± 0.002	22.4 ± 1.4
	B_4	A_3	0.039 ± 0.001	17.8 ± 0.5
		$A_{3.5}$	0.039 ± 0.001	17.8 ± 0.5
		A_4	0.036 ± 0.001	19.3 ± 0.5
		B_3	0.050 ± 0.003	13.9 ± 0.8
		$B_{3.5}$	0.060 ± 0.002	11.6 ± 0.4
		B_4	0.041 ± 0.001	16.9 ± 0.4
C-$A_{3.5}$	B_3	A_3	0.041 ± 0.001	16.9 ± 0.4
		$A_{3.5}$	0.050 ± 0.001	13.9 ± 0.3
		A_4	0.040 ± 0.001	17.3 ± 0.4
		B_3	0.050 ± 0.002	13.9 ± 0.6
		$B_{3.5}$	0.040 ± 0.001	17.3 ± 0.4
		B_4	0.024 ± 0.001	28.9 ± 1.2
	$B_{3.5}$	A_3	0.030 ± 0.004	23.1 ± 3.1
		$A_{3.5}$	0.036 ± 0.001	19.3 ± 0.5
		A_4	0.053 ± 0.001	13.1 ± 0.3
		B_3	0.032 ± 0.002	21.7 ± 1.4
		$B_{3.5}$	0.088 ± 0.003	7.9 ± 0.3
		B_4	0.060 ± 0.002	11.6 ± 0.4
	B_4	A_3	n.a.[a]	n.a.[a]
		$A_{3.5}$	0.022 ± 0.001	31.5 ± 1.4
		A_4	0.017 ± 0.001	40.8 ± 2.4
		B_3	n.a.[a]	n.a.[a]
		$B_{3.5}$	0.046 ± 0.004	15.1 ± 1.3
		B_4	0.074 ± 0.001	9.4 ± 0.1
C-A_4	B_3	A_3	0.025 ± 0.003	27.7 ± 3.3
		$A_{3.5}$	0.054 ± 0.001	12.8 ± 0.2
		A_4	0.030 ± 0.001	23.1 ± 0.8
		B_3	0.026 ± 0.003	26.7 ± 3.1
		$B_{3.5}$	0.054 ± 0.001	12.8 ± 0.2
		B_4	0.063 ± 0.002	11.0 ± 0.3
	$B_{3.5}$	A_3	n.a.[a]	n.a.[a]
		$A_{3.5}$	0.059 ± 0.001	11.7 ± 0.2
		A_4	0.049 ± 0.001	14.1 ± 0.3
		B_3	0.034 ± 0.004	20.4 ± 2.4
		$B_{3.5}$	0.061 ± 0.002	11.4 ± 0.4
		B_4	0.067 ± 0.001	10.3 ± 0.2
	B_4	A_3	n.a.[a]	n.a.[a]
		$A_{3.5}$	0.018 ± 0.002	38.5 ± 4.3
		A_4	0.019 ± 0.002	36.5 ± 3.8
		B_3	n.a.[a]	n.a.[a]
		$B_{3.5}$	n.a.[a]	n.a.[a]
		B_4	0.012 ± 0.001	57.8 ± 4.8

[a]Data did not fit the single exponential model.

Kinetic analysis of the time-resolved displacement using DynaFit

Fluorescence data of time-resolved strand displacement were fit to a competitive binding model using the fitting software DynaFit.[2,3] DynaFit uses a general numerical method for the determination of rate constants that characterize simultaneous and competitive binding of a ligand to two receptors R_1 and R_2 presuming a dissociative pathway for receptor displacement. In the context of heterodimeric coiled coils of the type AB R_1 and R_2 can be regarded as A and A_{comp} or as B and B_{comp}, respectively. A dissociative pathway for coil-strand displacement can be presumed as the underlying coiled-coil design results in non-promiscuous coiled-coil interaction.

$$A + B \underset{k_2}{\overset{k_1}{\rightleftharpoons}} AB$$

$$A + B_{comp} \underset{k_4}{\overset{k_3}{\rightleftharpoons}} AB_{comp} \quad \text{or} \quad A_{comp} + B \underset{k_4}{\overset{k_3}{\rightleftharpoons}} A_{comp}B$$

Scheme S1. Presumed dissociative mechanism of strand-displacement in AB heterodimeric coiled coils.

The time-resolved fluorescence data was converted into concentration of free A or B in the time course of displacement by A_{comp} or B_{comp} and competitive reformation of the AB coiled coil. Therefore, F_{max} was assigned to zero conversion of the initial AB coiled-coil complex (15 µM AB and 15 µM P_{comp}) and F_{min} was assigned to full strand displacement in AB (15 µM AB_{comp} or $A_{comp}B$ and 15 µM A or B) to AP_{comp} or $P_{comp}B$, respectively. We presumed that strand-displacement kinetics are the same in coiled coils of the same lengths of the individual strands whether or not labelled or unlabelled. Fitting of both events, displacement of A or B form the initial AB complex and re-association, gives relative rate constants k_1 to k_4 from which the overall affinities (k_1k_4/k_2k_3) can be calculated. The results are summarized in tables S5 and S6. The curve fitting is shown in figures S8-S13.

Table S 7. Best-fit values of rate constants of competitive strand displacement in N-A_xB_y peptides obtained from DynaFit analysis. Ratio of overall affinities (k_1k_4/k_2k_3) matches well with the ratio of the K_D values determined from CD thermal denaturation curves. P_{comp} is the general term for peptide competitor (A_{comp} or B_{comp}).

AB peptide		P_{comp}	k_1 [µM^{-1}s^{-1}]	k_2 [s^{-1}]	k_3 [µM^{-1}s^{-1}]	k_4 [s^{-1}]	k_1k_4/k_2k_3	K_D(comp)/K_D
N-A_3	B_3	A_3	0.14 ± 0.05	0.02 ± 0.002	0.15 ± 0.04	0.02 ± 0.002	0.93	1.0
		$A_{3.5}$	0.16 ± 0.08	0.019 ± 0.004	0.05 ± 0.01	(7.0 ± 1.2) · 10^{-4}	0.12	0.18
		A_4	0.19 ± 0.07	0.064 ± 0.004	0.28 ± 0.08	(1.7 ± 0.2) · 10^{-3}	0.02	0.06
		B_3	0.10 ± 0.03	(6.0 ± 0.8) · 10^{-3}	0.10 ± 0.04	(6.0 ± 0.8) · 10^{-3}	1.0	1.0
		$B_{3.5}$	0.20 ± 0.05	0.03 ± 0.002	0.38 ± 0.06	0.01 ± 0.0008	0.18	0.43
		B_4	0.21 ± 0.05	0.04 ± 0.002	0.32 ± 0.07	(9.0 ± 0.6) · 10^{-3}	0.15	0.20
	$B_{3.5}$	A_3	0.29 ± 0.08	0.012 ± 0.0007	0.29 ± 0.06	0.012 ± 0.0007	1.0	1.0
		$A_{3.5}$	0.30 ± 0.13	0.03 ± 0.002	0.80 ± 0.24	(1.2 ± 0.2) · 10^{-3}	0.02	0.003
		A_4	0.28 ± 0.07	0.056 ± 0.002	0.88 ± 0.17	(3.4 ± 0.3) · 10^{-3}	0.02	< 0.001
		B_3	0.20 ± 0.05	0.01 ± 0.0007	0.13 ± 0.03	0.03 ± 0.002	4.6	2.3
		$B_{3.5}$	0.36 ± 0.12	0.02 ± 0.001	0.36 ± 0.10	0.02 ± 0.001	1.0	1.0
		B_4	0.36 ± 0.11	0.024 ± 0.001	0.77 ± 0.19	0.023 ± 0.002	0.45	0.46
	B_4	A_3	0.18 ± 0.08	(3.0 ± 0.5) · 10^{-3}	0.18 ± 0.21	(3.0 ± 0.5) · 10^{-3}	1.0	1.0
		$A_{3.5}$	0.20 ± 0.08	0.10 ± 0.01	0.09 ± 0.03	(1.4 ± 0.1) · 10^{-3}	0.03	0.001
		A_4	0.37 ± 0.11	0.11 ± 0.004	1.18 ± 0.29	(7.4 ± 1.2) · 10^{-4}	0.02	< 0.001
		B_3	0.22 ± 0.05	(8.5 ± 0.5) · 10^{-3}	0.14 ± 0.03	0.04 ± 0.003	7.4	5.1
		$B_{3.5}$	0.43 ± 0.21	0.022 ± 0.002	0.20 ± 0.09	0.029 ± 0.002	2.8	2,27
		B_4	0.44 ± 0.3	0.041 ± 0.02	0.43 ± 0.34	0.041 ± 0.007	1.0	1.0
N-$A_{3.5}$	B_3	A_3	0.06 ± 0.02	(6.8 ± 1.1) · 10^{-4}	0.21 ± 0.11	0.02 ± 0.004	8.4	5.5
		$A_{3.5}$	0.28 ± 0.09	0.036 ± 0.003	0.28 ± 0.08	0.037 ± 0.003	1.03	1.0
		A_4	0.46 ± 0.17	0.11 ± 0.006	0.29 ± 0.11	(9.5 ± 0.5) · 10^{-3}	0.14	0.32
		B_3	0.27 ± 0.09	0.017 ± 0.01	0.26 ± 0.3	0.017 ± 0.006	1.04	1.0
		$B_{3.5}$	0.16 ± 0.03	0.02 ± 0.0009	0.99 ± 0.14	0.01 ± 0.002	0.08	0.008
		B_4	0.25 ± 0.09	0.07 ± 0.004	0.31 ± 0.1	(4.1 ± 0.3) · 10^{-3}	0.05	0.001
	$B_{3.5}$	A_3	0.39 ± 0.24	(2.5 ± 0.4) · 10^{-3}	0.11 ± 0.06	0.03 ± 0.002	42.5	> 100
		$A_{3.5}$	0.26 ± 0.05	0.028 ± 0.006	0.31 ± 0.11	0.03 ± 0.002	0.90	1.0
		A_4	0.36 ± 0.14	0.048 ± 0.003	0.28 ± 0.09	0.01 ± 0.0006	0.30	0.14
		B_3	0.36 ± 0.19	0.01 ± 0.003	0.05 ± 0.02	0.02 ± 0.0007	14.4	> 100
		$B_{3.5}$	0.32 ± 0.07	0.037 ± 0.002	0.33 ± 0.06	0.04 ± 0.002	1.0	1.0
		B_4	0.44 ± 0.18	0.094 ± 0.007	0.16 ± 0.06	0.01 ± 0.0005	0.35	0.16
	B_4	A_3	0.17 ± 0.05	(1.4 ± 0.1) · 10^{-3}	0.38 ± 0.12	0.09 ± 0.01	28.8	> 100
		$A_{3.5}$	0.20 ± 0.11	(6.4 ± 1.0) · 10^{-3}	0.20 ± 0.10	(6.4 ± 1.0) · 10^{-3}	1.0	1.0
		A_4	0.19 ± 0.042	0.02 ± 0.0009	0.50 ± 0.07	0.01 ± 0.0009	0.19	0.55
		B_3	0.16 ± 0.09	(4.2 ± 0.2) · 10^{-3}	0.14 ± 0.03	0.07 ± 0.006	19.0	> 100
		$B_{3.5}$	0.37 ± 0.06	0.011 ± 0.0005	0.91 ± 0.12	0.09 ± 0.011	3.5	6.4
		B_4	0.28 ± 0.06	0.03 ± 0.001	0.28 ± 0.05	0.03 ± 0.001	1.0	1.0
N-A_4	B_3	A_3	0.31 ± 0.18	(1.4 ± 0.2) · 10^{-3}	0.34 ± 0.2	(9.2 ± 1.3) · 10^{-2}	6.0	5.1
		$A_{3.5}$	0.39 ± 0.06	(6.7 ± 0.4) · 10^{-3}	1.51 ± 0.3	0.21 ± 0.05	8.1	2.2
		A_4	0.58 ± 0.13	0.02 ± 0.0007	0.50 ± 0.10	0.02 ± 0.0007	1.10	1.0
		B_3	0.14 ± 0.03	0.04 ± 0.01	0.14 ± 0.12	0.04 ± 0.007	1.0	1.0
		$B_{3.5}$	0.22 ± 0.04	0.12 ± 0.004	0.51 ± 0.09	(5.7 ± 0.6) · 10^{-4}	0.002	0.004
		B_4	0.20 ± 0.04	0.096 ± 0.003	0.84 ± 0.13	(1.5 ± 0.2) · 10^{-3}	0.004	0.002
	$B_{3.5}$	A_3	0.29 ± 0.1	(4.1 ± 0.4) · 10^{-3}	0.07 ± 0.02	0.06 ± 0.003	60.6	> 100
		$A_{3.5}$	0.24 ± 0.07	0.01 ± 0.0009	0.21 ± 0.05	0.03 ± 0.002	3.4	6.9
		A_4	0.20 ± 0.05	0.026 ± 0.002	0.20 ± 0.04	0.025 ± 0.002	0.96	1.0
		B_3	0.19 ± 0.03	(9.9 ± 0.9) · 10^{-4}	0.03 ± 0.007	0.11 ± 0.003	> 100	> 100
		$B_{3.5}$	0.22 ± 0.05	0.03 ± 0.002	0.24 ± 0.04	0.04 ± 0.002	1.22	1.0
		B_4	0.27 ± 0.08	0.09 ± 0.006	0.09 ± 0.02	0.01 ± 0.0005	0.33	0.62
	B_4	A_3	0.34 ± 0.13	(8.1 ± 0.9) · 10^{-4}	0.14 ± 0.05	0.13 ± 0.008	> 100	> 100
		$A_{3.5}$	0.17 ± 0.04	0.01 ± 0.0009	0.07 ± 0.01	0.02 ± 0.001	4.9	1.7
		A_4	0.11 ± 0.24	0.013 ± 0.04	0.11 ± 0.30	0.013 ± 0.04	1.0	1.0
		B_3	0.25 ± 0.06	(1.2 ± 0.1) · 10^{-3}	0.07 ± 0.02	0.11 ± 0.005	> 100	> 100
		$B_{3.5}$	0.17 ± 0.02	0.01 ± 0.0004	0.38 ± 0.03	0.07 ± 0.006	3.1	1.6
		B_4	0.32 ± 0.08	0.013 ± 0.0006	0.32 ± 0.06	0.013 ± 0.0006	1.0	1.0

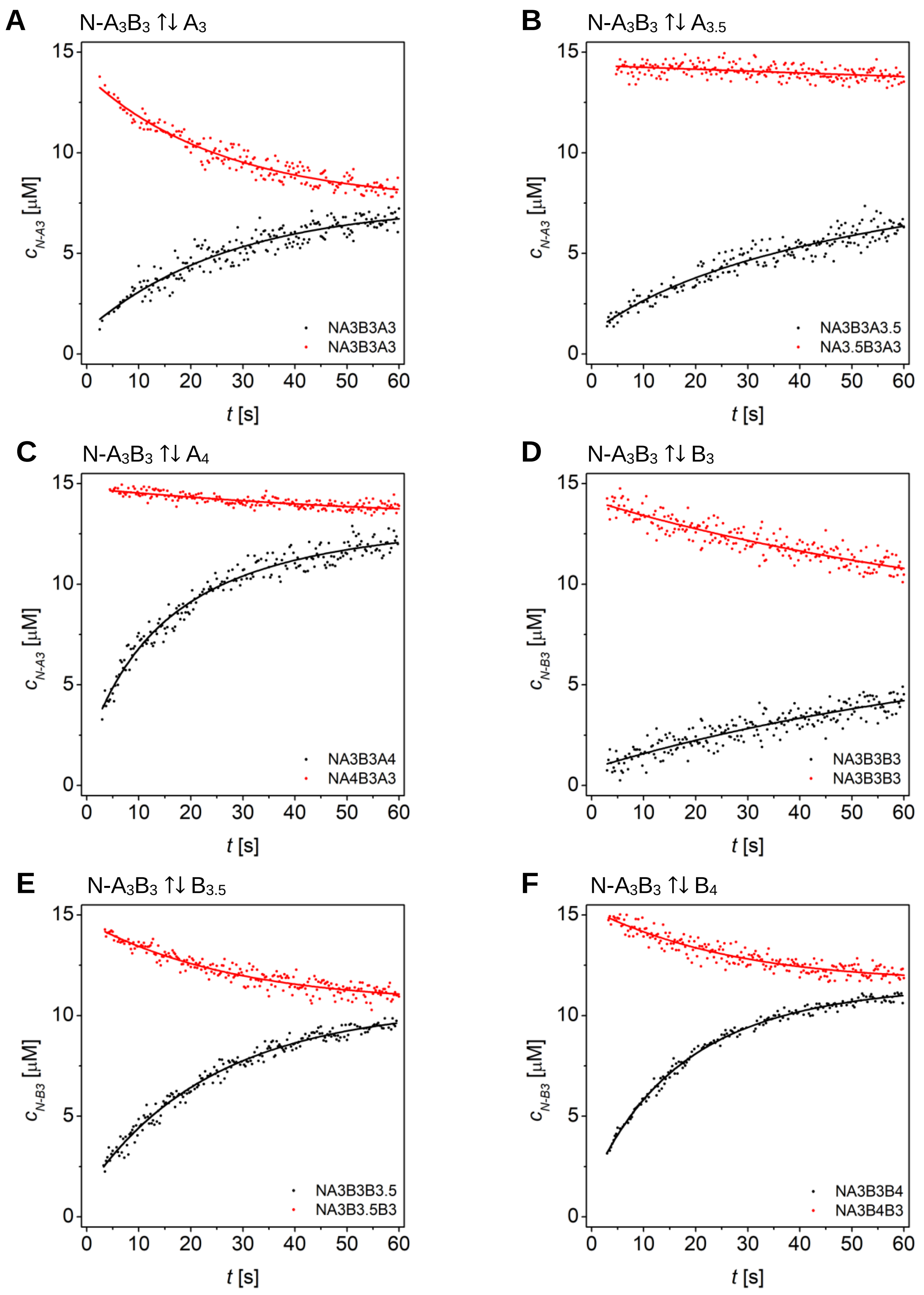
A
N-A3B3 ↑↓ A3
NA3B3A3
NA3B3A3
B
N-A3B3 ↑↓ A3.5
NA3B3A3.5
NA3.5B3A3
C
N-A3B3 ↑↓ A4
NA3B3A4
NA4B3A3
D
N-A3B3 ↑↓ B3
NA3B3B3
NA3B3B3
E
N-A3B3 ↑↓ B3.5
NA3B3B3.5
NA3B3.5B3
F
N-A3B3 ↑↓ B4
NA3B3B4
NA3B4B3
c N-A3 [μM]
c N-B3 [μM]
t [s]

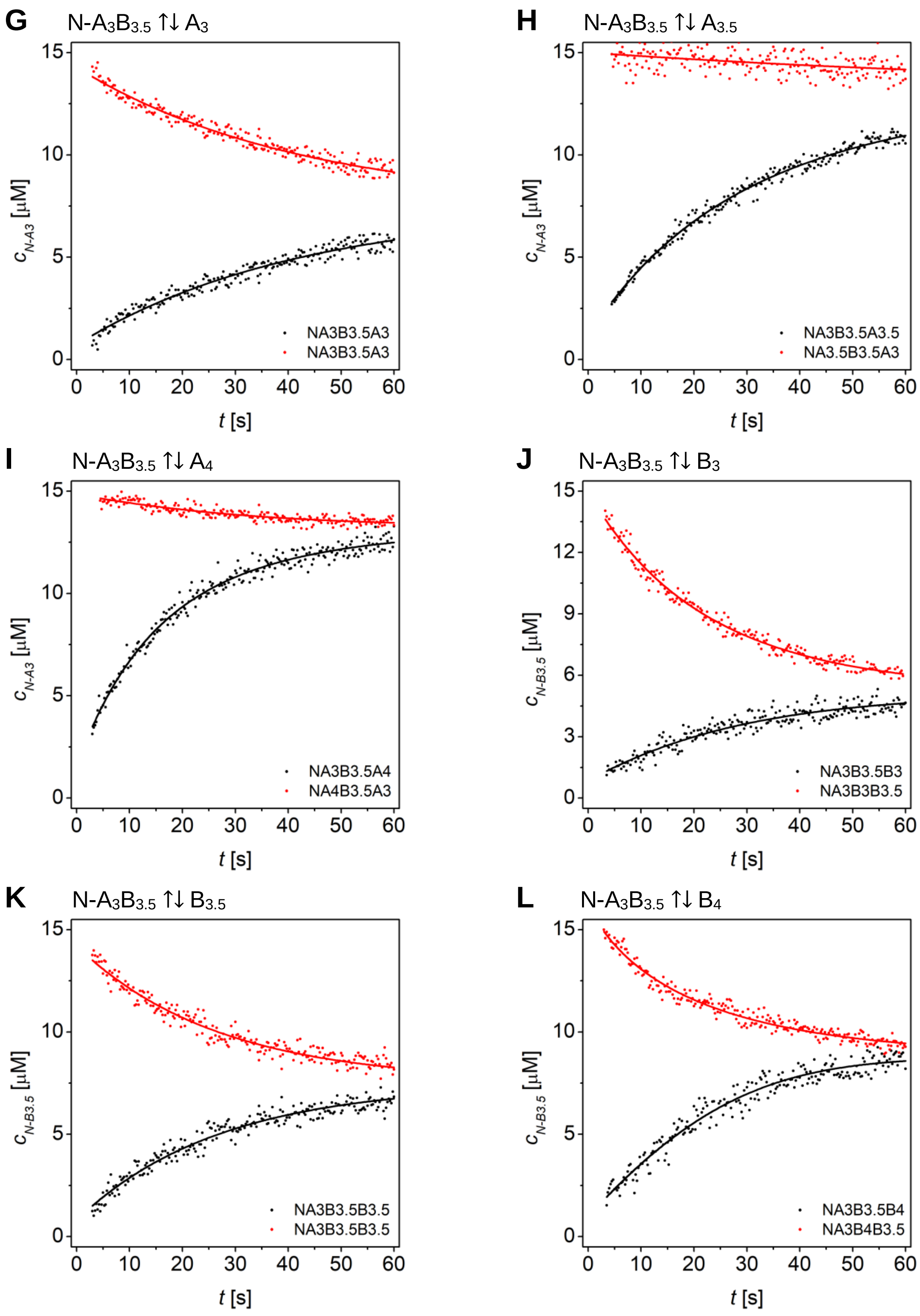
G
N-A3B3.5 ⇅ A3
c N-A3 [μM]
t [s]
NA3B3.5A3
NA3B3.5A3
H
N-A3B3.5 ⇅ A3.5
c N-A3 [μM]
t [s]
NA3B3.5A3.5
NA3.5B3.5A3
I
N-A3B3.5 ⇅ A4
c N-A3 [μM]
t [s]
NA3B3.5A4
NA4B3.5A3
J
N-A3B3.5 ⇅ B3
c N-B3.5 [μM]
t [s]
NA3B3.5B3
NA3B3B3.5
K
N-A3B3.5 ⇅ B3.5
c N-B3.5 [μM]
t [s]
NA3B3.5B3.5
NA3B3.5B3.5
L
N-A3B3.5 ⇅ B4
c N-B3.5 [μM]
t [s]
NA3B3.5B4
NA3B4B3.5

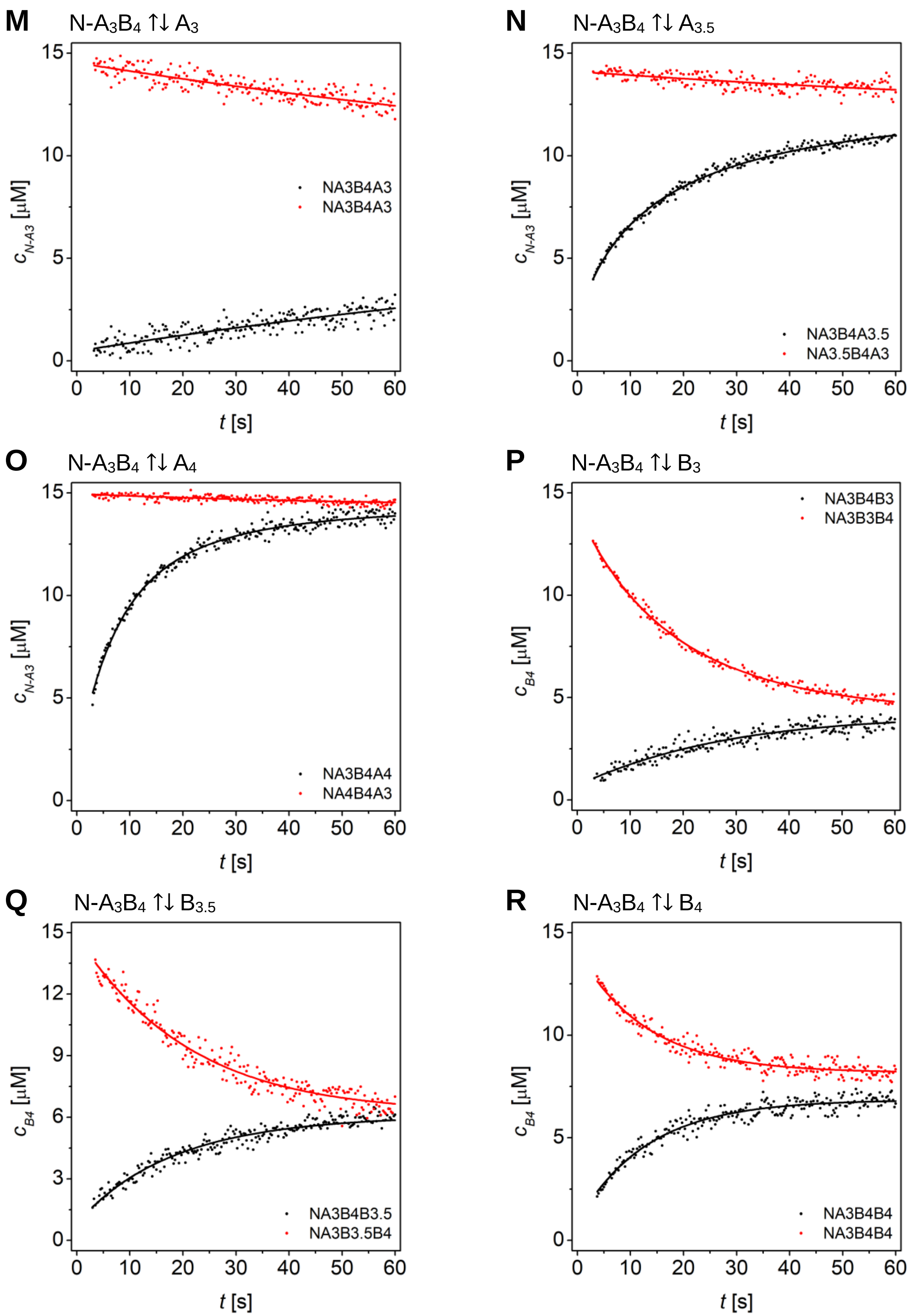

Figure S8. Dynafit results of least-squares fits of strand displacement in N-A_3B peptides using a competitive binding model.

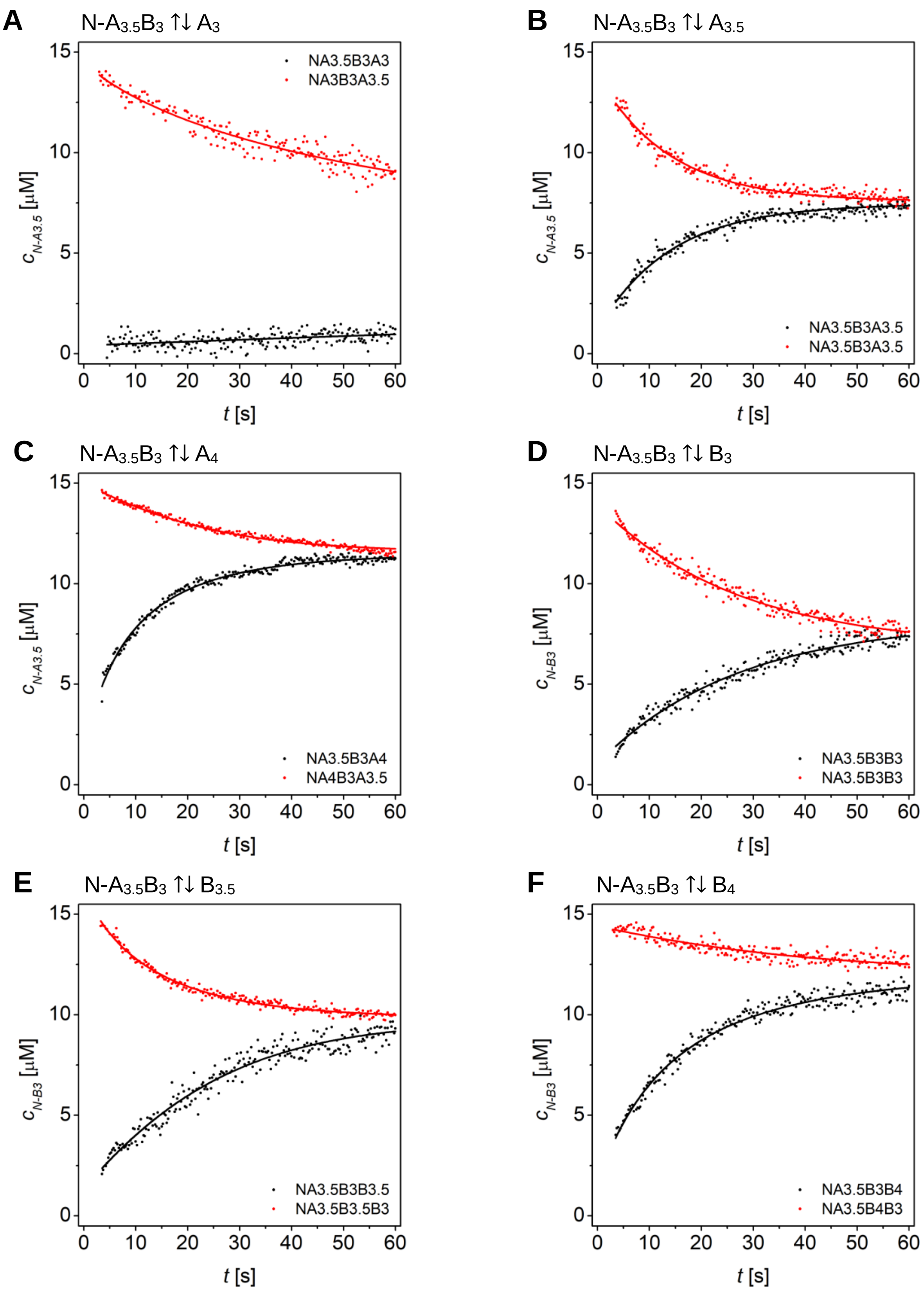
A
N-$A_{3.5}B_3$ ↑↓ A_3
$c_{N-A3.5}$ [μM]
t [s]
NA3.5B3A3
NA3B3A3.5
B
N-$A_{3.5}B_3$ ↑↓ $A_{3.5}$
$c_{N-A3.5}$ [μM]
t [s]
NA3.5B3A3.5
NA3.5B3A3.5
C
N-$A_{3.5}B_3$ ↑↓ A_4
$c_{N-A3.5}$ [μM]
t [s]
NA3.5B3A4
NA4B3A3.5
D
N-$A_{3.5}B_3$ ↑↓ B_3
c_{N-B3} [μM]
t [s]
NA3.5B3B3
NA3.5B3B3
E
N-$A_{3.5}B_3$ ↑↓ $B_{3.5}$
c_{N-B3} [μM]
t [s]
NA3.5B3B3.5
NA3.5B3.5B3
F
N-$A_{3.5}B_3$ ↑↓ B_4
c_{N-B3} [μM]
t [s]
NA3.5B3B4
NA3.5B4B3

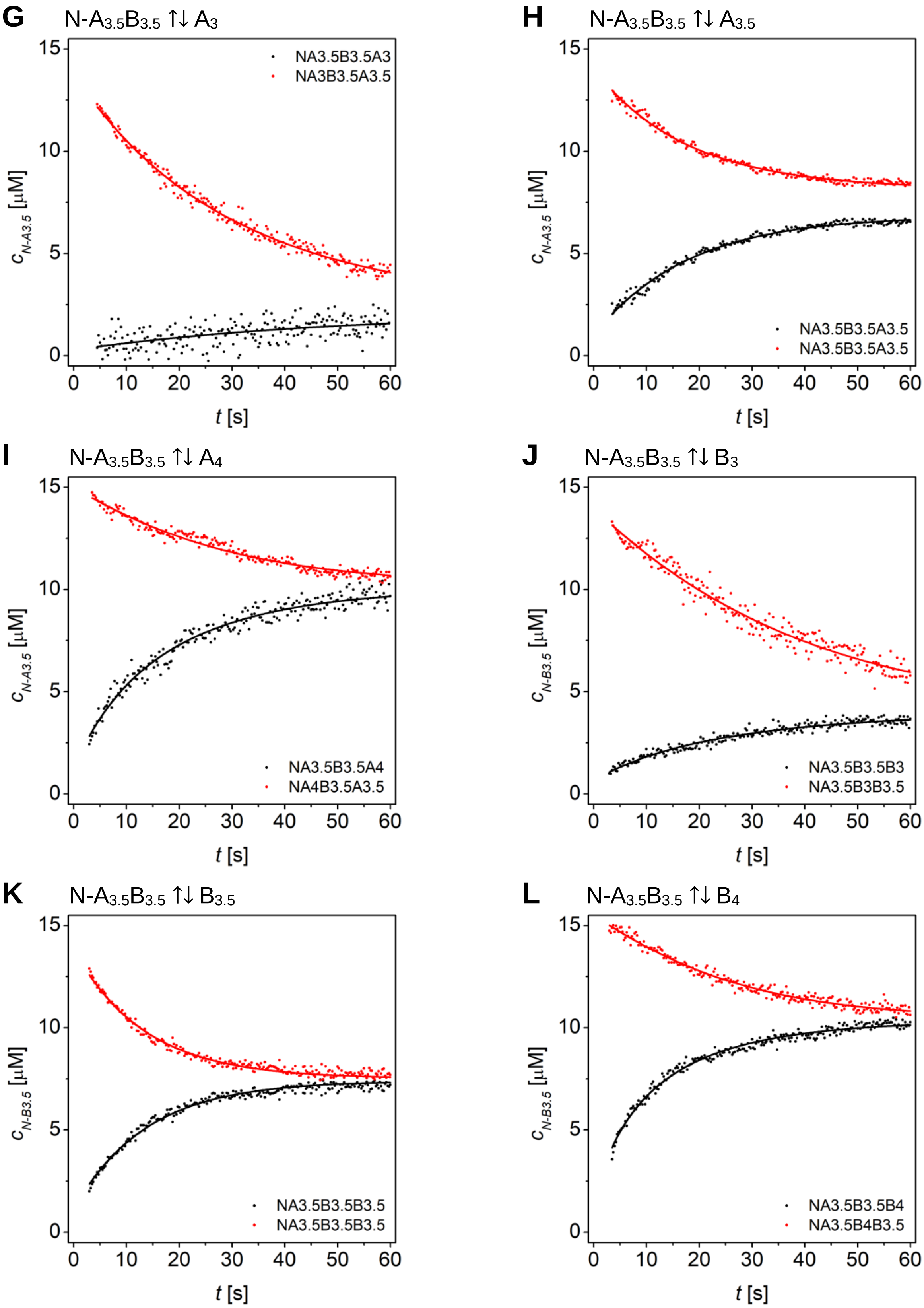
G
N-A3.5B3.5 ↑↓ A3
NA3.5B3.5A3
NA3B3.5A3.5
H
N-A3.5B3.5 ↑↓ A3.5
NA3.5B3.5A3.5
NA3.5B3.5A3.5
I
N-A3.5B3.5 ↑↓ A4
NA3.5B3.5A4
NA4B3.5A3.5
J
N-A3.5B3.5 ↑↓ B3
NA3.5B3.5B3
NA3.5B3B3.5
K
N-A3.5B3.5 ↑↓ B3.5
NA3.5B3.5B3.5
NA3.5B3.5B3.5
L
N-A3.5B3.5 ↑↓ B4
NA3.5B3.5B4
NA3.5B4B3.5
c N-A3.5 [µM]
c N-B3.5 [µM]
t [s]

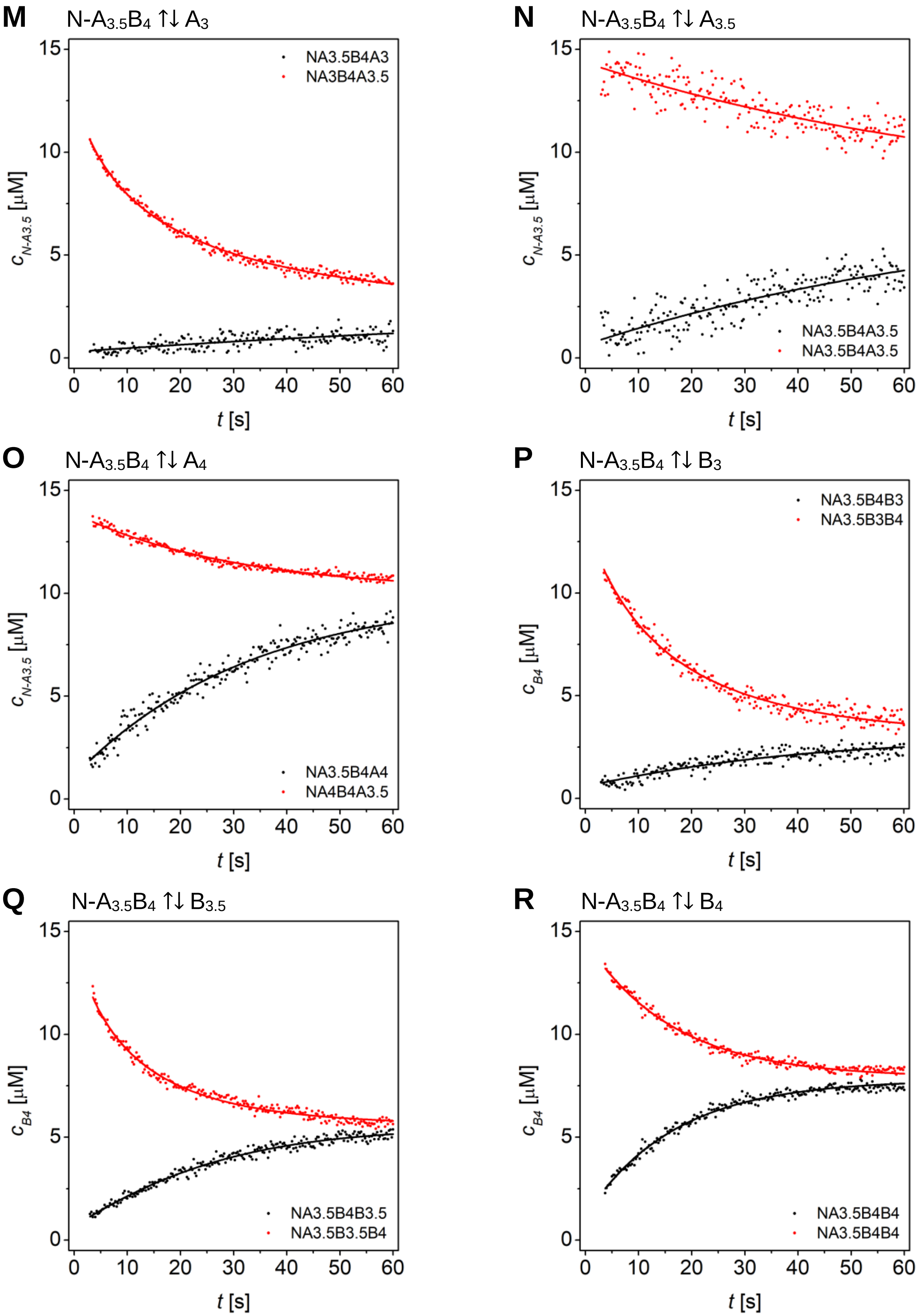

Figure S9. Dynafit results of least-squares fits strand displacement in N-$A_{3.5}$B peptides using a competitive binding model.

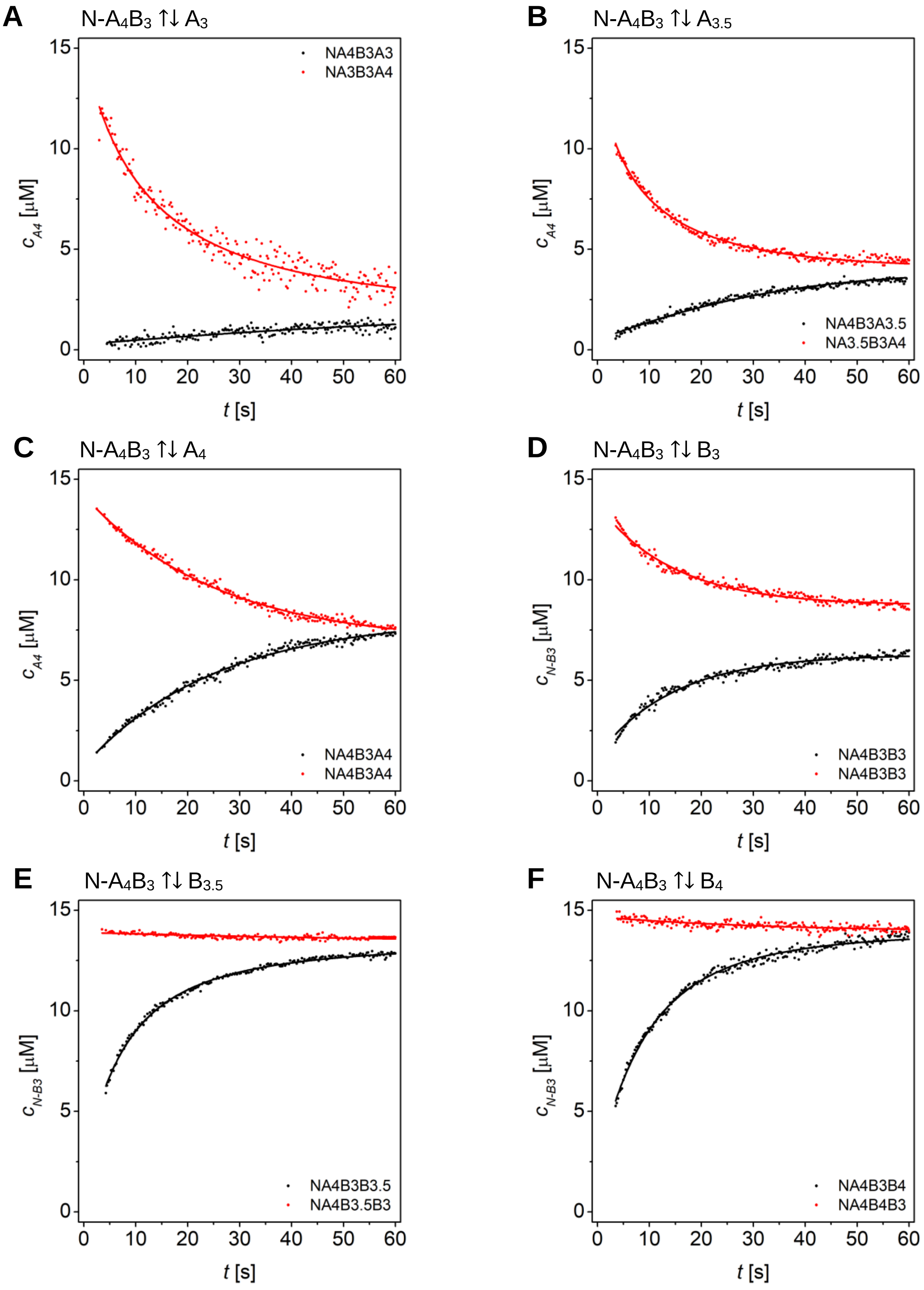
A
N-A4B3 ⇅ A3
NA4B3A3
NA3B3A4
B
N-A4B3 ⇅ A3.5
NA4B3A3.5
NA3.5B3A4
C
N-A4B3 ⇅ A4
NA4B3A4
NA4B3A4
D
N-A4B3 ⇅ B3
NA4B3B3
NA4B3B3
E
N-A4B3 ⇅ B3.5
NA4B3B3.5
NA4B3.5B3
F
N-A4B3 ⇅ B4
NA4B3B4
NA4B4B3
c_A4 [μM]
c_N-B3 [μM]
t [s]

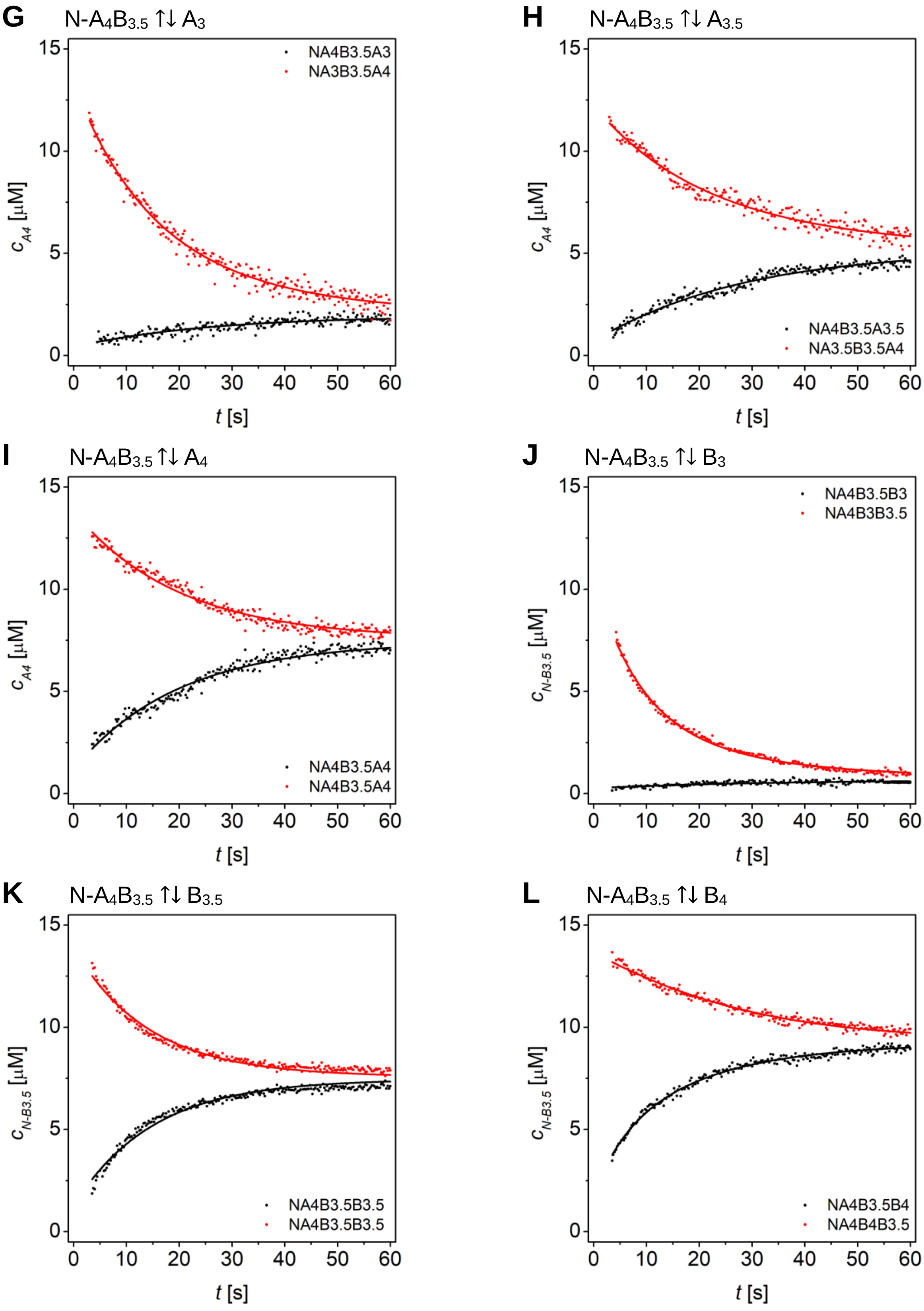
G
N-A4B3.5 ↑↓ A3
NA4B3.5A3
NA3B3.5A4
H
N-A4B3.5 ↑↓ A3.5
NA4B3.5A3.5
NA3.5B3.5A4
I
N-A4B3.5 ↑↓ A4
NA4B3.5A4
NA4B3.5A4
J
N-A4B3.5 ↑↓ B3
NA4B3.5B3
NA4B3B3.5
K
N-A4B3.5 ↑↓ B3.5
NA4B3.5B3.5
NA4B3.5B3.5
L
N-A4B3.5 ↑↓ B4
NA4B3.5B4
NA4B4B3.5
c_A4 [μM]
c_N-B3.5 [μM]
t [s]
0
5
10
15
0
10
20
30
40
50
60

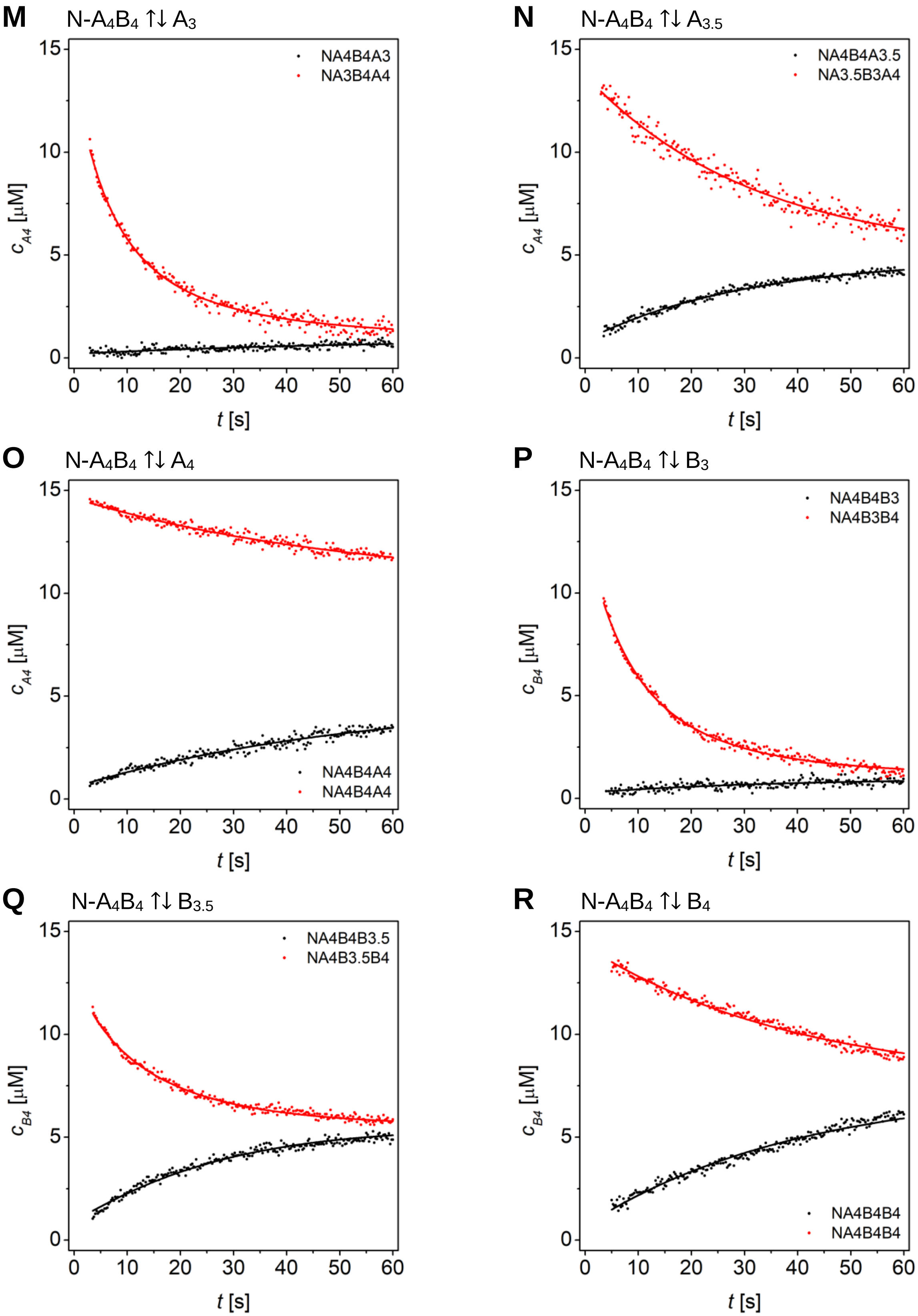

Figure S10. Dynafit results of least-squares fits strand displacement in N-A_4B peptides using a competitive binding model.

Table S8. Best-fit values of rate constants of competitive strand displacement in C-A_xB_y peptides obtained from DynaFit analysis. Ratio of overall affinities (k_1k_4/k_2k_2) matches well with the ration of the K_D values determined from CD-thermal denaturation curves. P_{comp} is the general term for peptide competitor (A_{comp} or B_{comp}).

AB peptide		P_{comp}	k_1 [µM^{-1}s^{-1}]	k_2 [s^{-1}]	k_3 [µM^{-1}s^{-1}]	k_4 [s^{-1}]	k_1k_4/k_2k_3	K_D(comp)/K_D
C-A_3	B_3	A_3	0.15 ± 0.02	0.01 ± 0.0006	0.15 ± 0.02	0.01 ± 0.0006	1.0	1.0
		$A_{3.5}$	0.15 ± 0.02	0.04 ± 0.002	0.17 ± 0.02	0.01 ± 0.0004	0.22	0.57
		A_4	0.23 ± 0.07	0.03 ± 0.002	0.17 ± 0.04	0.01 ± 0.0006	0.45	0.63
		B_3	0.13 ± 0.04	0.035 ± 0.02	0.13 ± 0.2	0.035 ± 0.01	1.0	1.0
		$B_{3.5}$	0.21 ± 0.06	0.07 ± 0.007	0.06 ± 0.01	$(8.9 \pm 0.4) \cdot 10^{-3}$	0.45	0.31
		B_4	0.21 ± 0.06	0.18 ± 0.02	0.06 ± 0.02	$(6.8 \pm 0.5) \cdot 10^{-3}$	0.13	0.18
	$B_{3.5}$	A_3	0.31 ± 0.06	0.021 ± 0.0009	0.25 ± 0.04	0.018 ± 0.0007	1.06	1.0
		$A_{3.5}$	0.13 ± 0.03	0.06 ± 0.003	0.17 ± 0.03	$(2.1 \pm 0.2) \cdot 10^{-3}$	0.03	0.06
		A_4	0.25 ± 0.03	0.047 ± 0.0007	4.9 ± 0.57	$(1.7 \pm 0.9) \cdot 10^{-4}$	< 0.001	0.04
		B_3	0.20 ± 0.03	$(8.9 \pm 0.3) \cdot 10^{-3}$	0.55 ± 0.08	0.05 ± 0.006	2.0	3.3
		$B_{3.5}$	0.17 ± 0.09	0.06 ± 0.04	0.17 ± 0.23	0.06 ± 0.01	1.0	1.0
		B_4	0.12 ± 0.02	0.02 ± 0.0006	0.59 ± 0.08	0.04 ± 0.006	0.41	0.57
	B_4	A_3	0.24 ± 0.04	0.02 ± 0.0006	0.24 ± 0.03	0.02 ± 0.0006	1.0	1.0
		$A_{3.5}$	0.11 ± 0.01	0.04 ± 0.001	0.29 ± 0.02	$(1.6 \pm 0.1) \cdot 10^{-3}$	0.02	0.008
		A_4	0.12 ± 0.02	0.04 ± 0.001	0.15 ± 0.01	$(9.4 \pm 5.1) \cdot 10^{-5}$	0.002	< 0.001
		B_3	0.12 ± 0.01	$(6.6 \pm 0.3) \cdot 10^{-3}$	0.30 ± 0.05	0.14 ± 0.03	8.5	5.7
		$B_{3.5}$	0.28 ± 0.11	0.03 ± 0.003	0.07 ± 0.02	0.02 ± 0.0007	2.7	1.7
		B_4	0.15 ± 0.02	0.02 ± 0.007	0.15 ± 0.12	0.02 ± 0.005	1.0	1.0
C-$A_{3.5}$	B_3	A_3	0.15 ± 0.02	0.01 ± 0.0004	0.12 ± 0.01	0.04 ± 0.002	5.0	1.8
		$A_{3.5}$	0.33 ± 0.05	0.02 ± 0.0007	0.33 ± 0.04	0.02 ± 0.0007	1.0	1.0
		A_4	0.29 ± 0.03	0.02 ± 0.0004	0.66 ± 0.05	0.03 ± 0.001	0.66	1.1
		B_3	0.35 ± 0.14	0.03 ± 0.018	0.35 ± 0.52	0.03 ± 0.01	1.0	1.0
		$B_{3.5}$	0.13 ± 0.03	0.19 ± 0.03	0.03 ± 0.003	$(5.2 \pm 0.2) \cdot 10^{-3}$	0.11	0.03
		B_4	0.45 ± 0.25	0.02 ± 0.002	0.19 ± 0.06	$(9.9 \pm 4.2) \cdot 10^{-5}$	0.01	0.003
	$B_{3.5}$	A_3	0.21 ± 0.06	$(1.7 \pm 0.1) \cdot 10^{-3}$	0.24 ± 0.06	0.07 ± 0.005	36	18
		$A_{3.5}$	0.21 ± 0.04	0.02 ± 0.006	0.21 ± 0.17	0.02 ± 0.004	1.0	1.0
		A_4	0.24 ± 0.03	0.03 ± 0.0007	0.40 ± 0.03	0.02 ± 0.0007	0.4	0.58
		B_3	0.26 ± 0.04	$(5.2 \pm 0.2) \cdot 10^{-3}$	0.36 ± 0.05	0.07 ± 0.005	9.7	31
		$B_{3.5}$	0.21 ± 0.06	0.05 ± 0.02	0.21 ± 0.13	0.05 ± 0.005	1.0	1.0
		B_4	0.16 ± 0.04	0.18 ± 0.02	0.05 ± 0.008	$(1.8 \pm 0.1) \cdot 10^{-3}$	0.03	0.10
	B_4	A_3	0.14 ± 0.03	$(1.4 \pm 0.09) \cdot 10^{-3}$	0.08 ± 0.02	0.04 ± 0.002	50	>100
		$A_{3.5}$	0.20 ± 0.03	0.01 ± 0.0004	0.20 ± 0.02	0.01 ± 0.0004	1.0	1.0
		A_4	0.18 ± 0.02	0.01 ± 0.002	0.92 ± 0.23	0.002 ± 0.0005	0.04	0.08
		B_3	0.15 ± 0.05	$(5.5 \pm 0.7) \cdot 10^{-4}$	0.05 ± 0.02	0.02 ± 0.0007	>100	>100
		$B_{3.5}$	0.14 ± 0.03	$(1.8 \pm 0.1) \cdot 10^{-3}$	0.27 ± 0.06	0.16 ± 0.03	46	12
		B_4	0.19 ± 0.03	0.03 ± 0.001	0.19 ± 0.02	0.03 ± 0.001	1.0	1.0
C-A_4	B_3	A_3	0.21 ± 0.04	0.01 ± 0.0006	0.25 ± 0.04	0.03 ± 0.002	2.5	1.6
		$A_{3.5}$	0.31 ± 0.05	0.03 ± 0.001	0.13 ± 0.02	0.02 ± 0.0004	1.59	0.9
		A_4	0.24 ± 0.02	0.016 ± 0.0004	0.21 ± 0.02	0.014 ± 0.0004	1.0	1.0
		B_3	0.16 ± 0.39	0.02 ± 0.07	0.17 ± 4.2	0.02 ± 0.08	1.0	1.0
		$B_{3.5}$	0.11 ± 0.07	0.46 ± 0.26	0.012 ± 0.0009	$(5.1 \pm 0.3) \cdot 10^{-3}$	0.10	0.02
		B_4	0.20 ± 0.09	0.63 ± 0.21	0.03 ± 0.004	$(1.3 \pm 0.1) \cdot 10^{-3}$	0.01	< 0.001
	$B_{3.5}$	A_3	0.24 ± 0.06	$(7.1 \pm 0.7) \cdot 10^{-4}$	$(1.6 \pm 0.5) \cdot 10^{-3}$	0.05 ± 0.0006	>100	27
		$A_{3.5}$	0.23 ± 0.03	0.01 ± 0.0004	0.17 ± 0.02	0.03 ± 0.0009	4.1	1.6
		A_4	0.29 ± 0.03	0.02 ± 0.0006	0.29 ± 0.03	0.02 ± 0.0006	1.0	1.0
		B_3	0.25 ± 0.05	$(3.3 \pm 0.1) \cdot 10^{-3}$	0.27 ± 0.05	0.06 ± 0.003	17	56
		$B_{3.5}$	0.25 ± 0.05	0.031 ± 0.002	0.20 ± 0.03	0.025 ± 0.001	1.0	1.0
		B_4	0.22 ± 0.07	0.45 ± 0.08	0.05 ± 0.009	$(9.1 \pm 0.9) \cdot 10^{-4}$	0.009	0.01
	B_4	A_3	0.16 ± 0.04	$(9.9 \pm 0.7) \cdot 10^{-4}$	0.13 ± 0.03	0.03 ± 0.001	37	>100
		$A_{3.5}$	0.15 ± 0.04	$(1.9 \pm 0.5) \cdot 10^{-3}$	0.01 ± 0.001	0.01 ± 0.0008	79	13
		A_4	0.11 ± 0.24	0.013 ± 0.04	0.11 ± 0.30	0.013 ± 0.04	1.0	1.0
		B_3	0.05 ± 0.009	$(1.1 \pm 0.06) \cdot 10^{-3}$	0.14 ± 0.03	0.21 ± 0.05	68	>100
		$B_{3.5}$	0.20 ± 0.06	$(9.2 \pm 0.8) \cdot 10^{-4}$	0.20 ± 0.06	0.13 ± 0.01	>100	97
		B_4	0.32 ± 0.08	0.013 ± 0.0006	0.32 ± 0.06	0.013 ± 0.0006	1.0	1.0

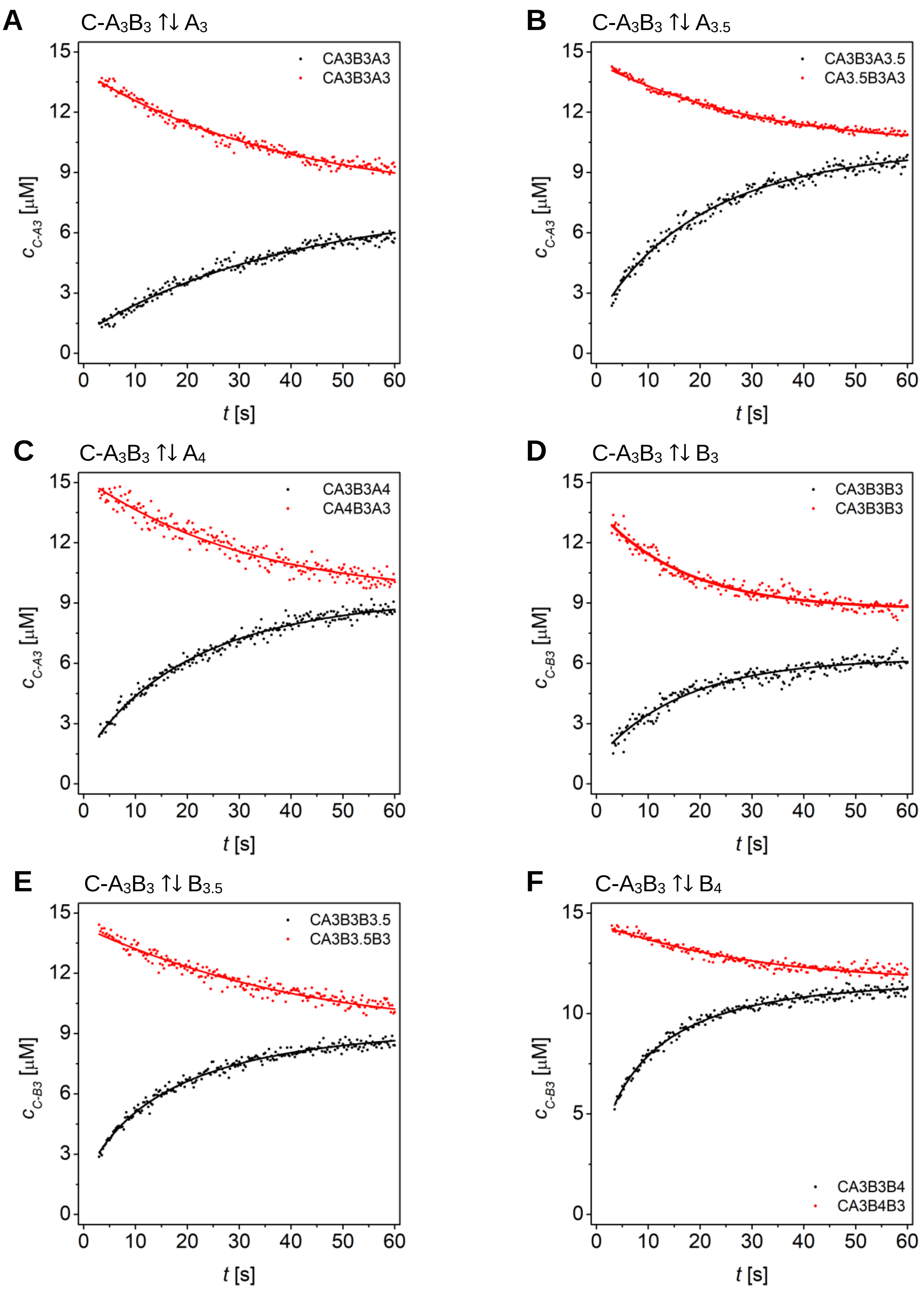
A
C-A3B3 ⇅ A3
CA3B3A3
CA3B3A3
B
C-A3B3 ⇅ A3.5
CA3B3A3.5
CA3.5B3A3
C
C-A3B3 ⇅ A4
CA3B3A4
CA4B3A3
D
C-A3B3 ⇅ B3
CA3B3B3
CA3B3B3
E
C-A3B3 ⇅ B3.5
CA3B3B3.5
CA3B3.5B3
F
C-A3B3 ⇅ B4
CA3B3B4
CA3B4B3
c C-A3 [μM]
c C-B3 [μM]
t [s]

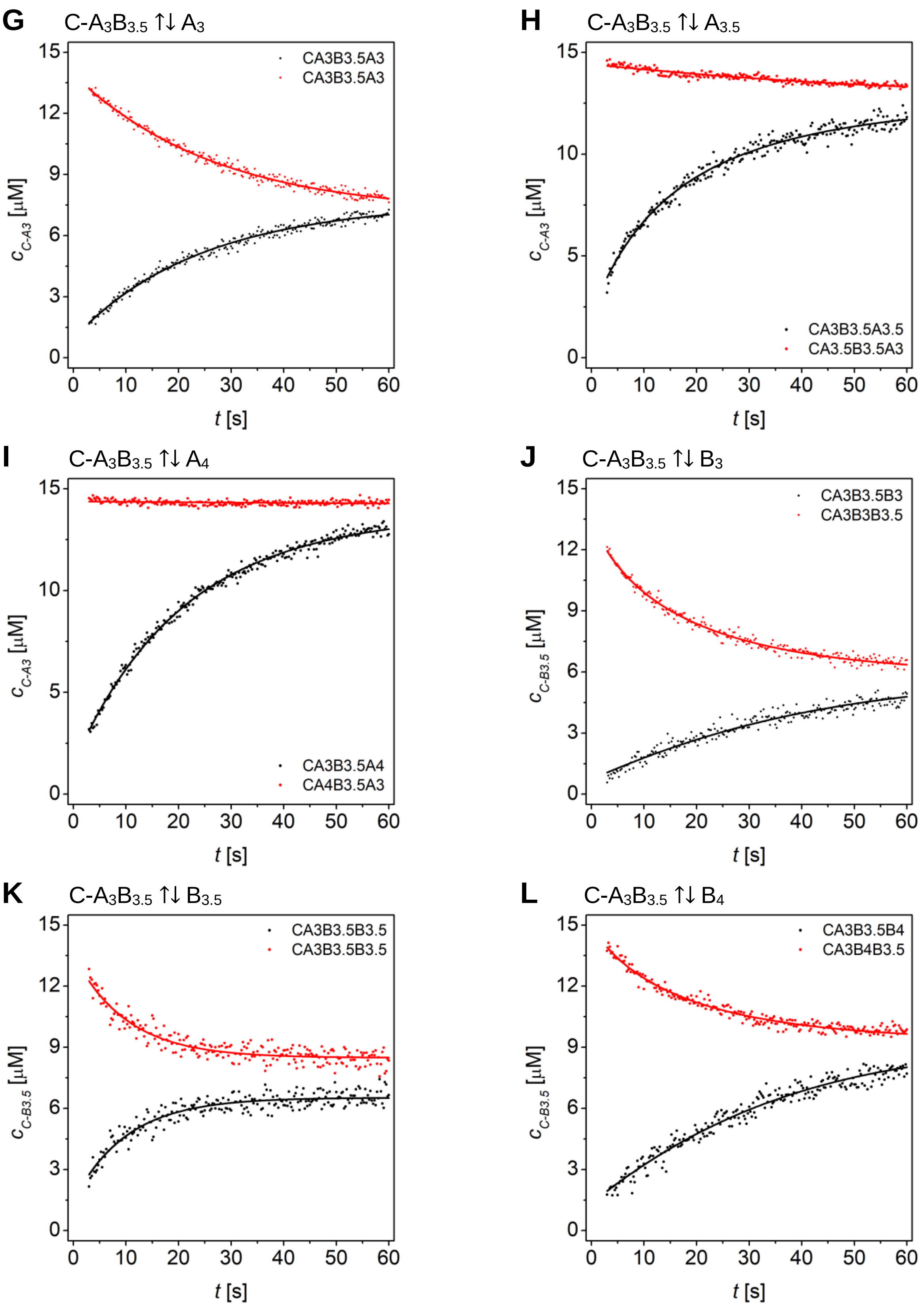
G
C-A3B3.5 ⮁ A3
CA3B3.5A3
CA3B3.5A3
cC-A3 [μM]
t [s]
H
C-A3B3.5 ⮁ A3.5
CA3B3.5A3.5
CA3.5B3.5A3
cC-A3 [μM]
t [s]
I
C-A3B3.5 ⮁ A4
CA3B3.5A4
CA4B3.5A3
cC-A3 [μM]
t [s]
J
C-A3B3.5 ⮁ B3
CA3B3.5B3
CA3B3B3.5
cC-B3.5 [μM]
t [s]
K
C-A3B3.5 ⮁ B3.5
CA3B3.5B3.5
CA3B3.5B3.5
cC-B3.5 [μM]
t [s]
L
C-A3B3.5 ⮁ B4
CA3B3.5B4
CA3B4B3.5
cC-B3.5 [μM]
t [s]

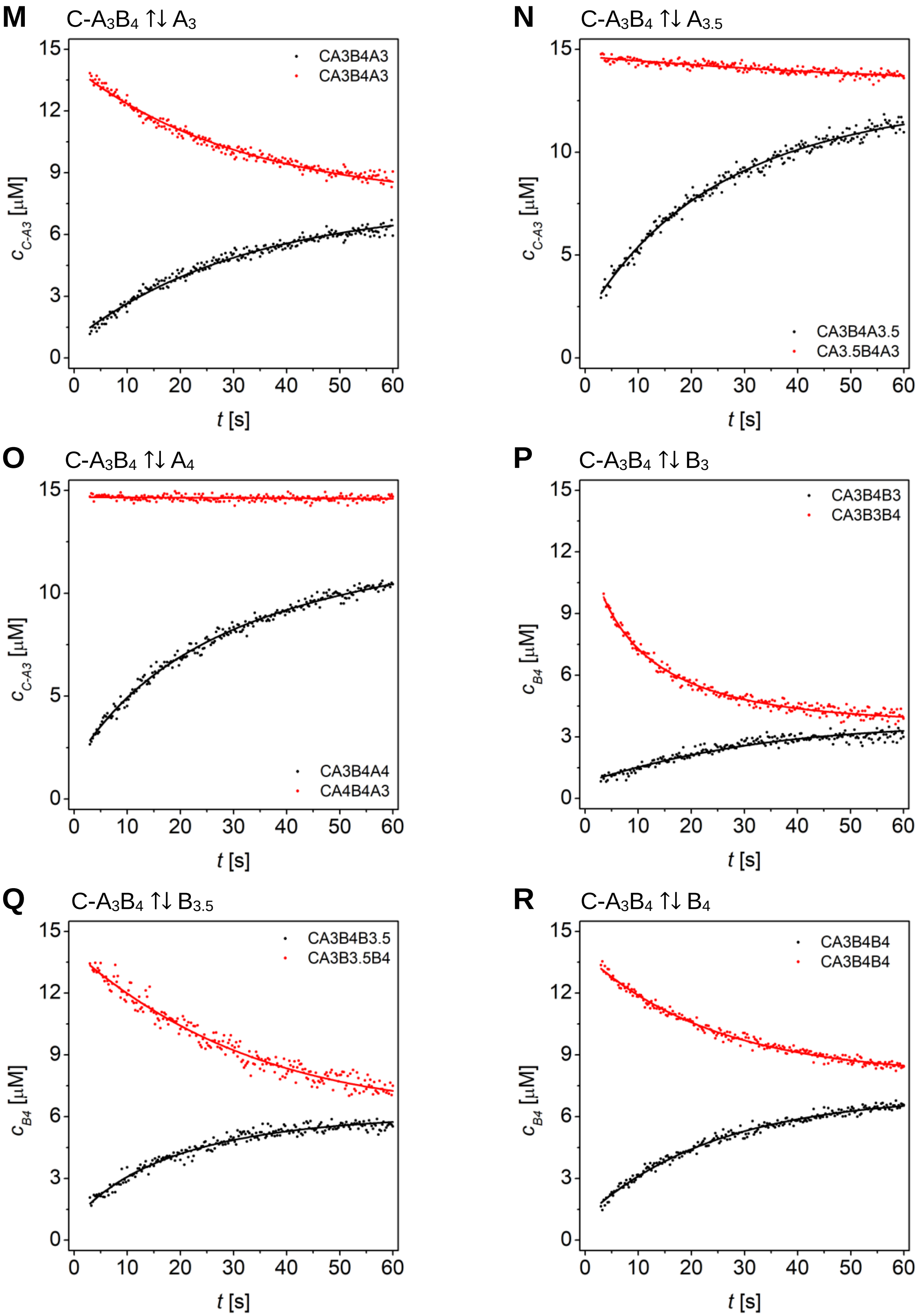

Figure S11. Dynafit results of least-squares fits strand displacement in C-A_3B peptides using a competitive binding model.

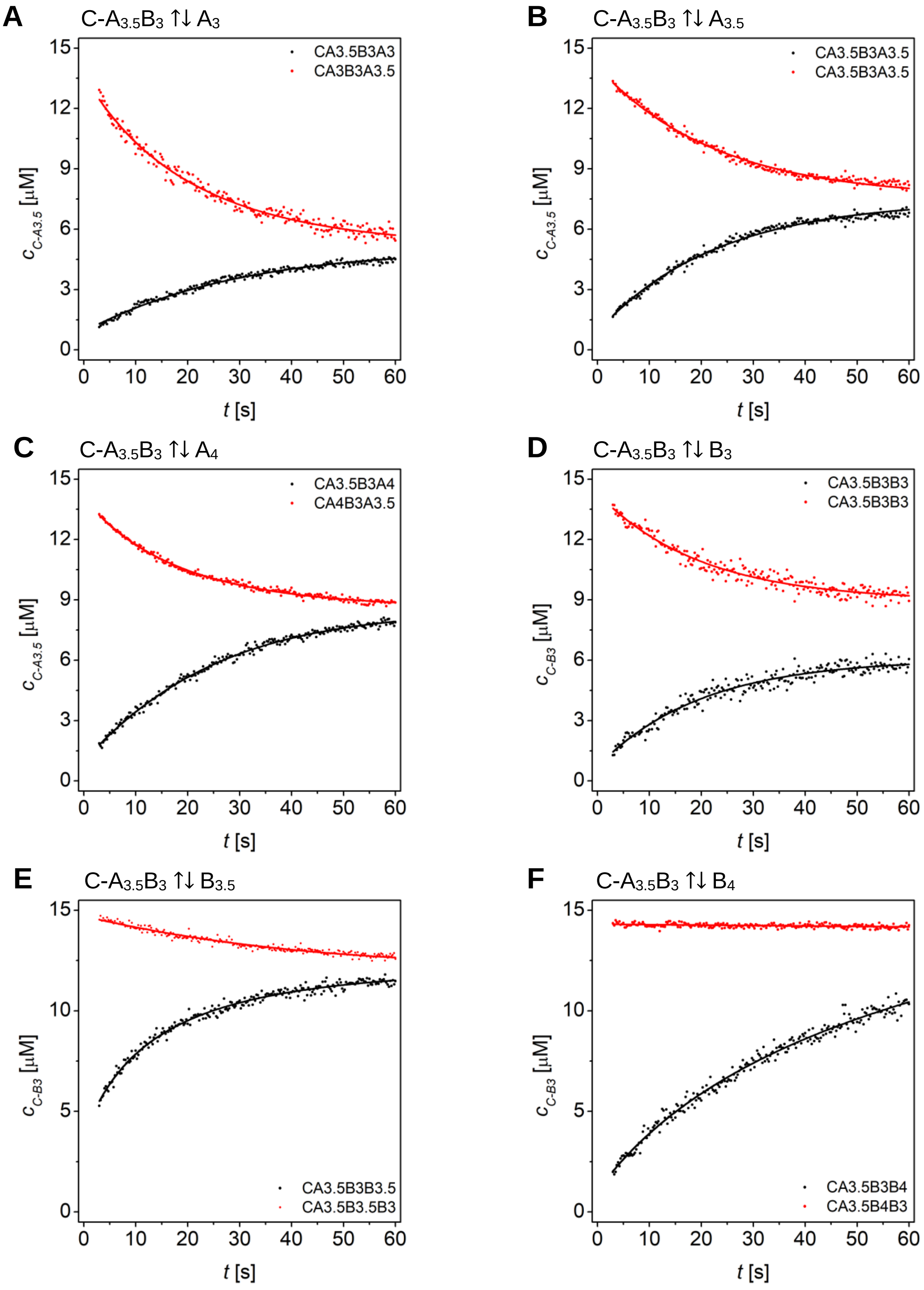
A
C-A3.5B3 ⇅ A3
CA3.5B3A3
CA3B3A3.5
B
C-A3.5B3 ⇅ A3.5
CA3.5B3A3.5
CA3.5B3A3.5
C
C-A3.5B3 ⇅ A4
CA3.5B3A4
CA4B3A3.5
D
C-A3.5B3 ⇅ B3
CA3.5B3B3
CA3.5B3B3
E
C-A3.5B3 ⇅ B3.5
CA3.5B3B3.5
CA3.5B3.5B3
F
C-A3.5B3 ⇅ B4
CA3.5B3B4
CA3.5B4B3
c C-A3.5 [μM]
c C-B3 [μM]
t [s]

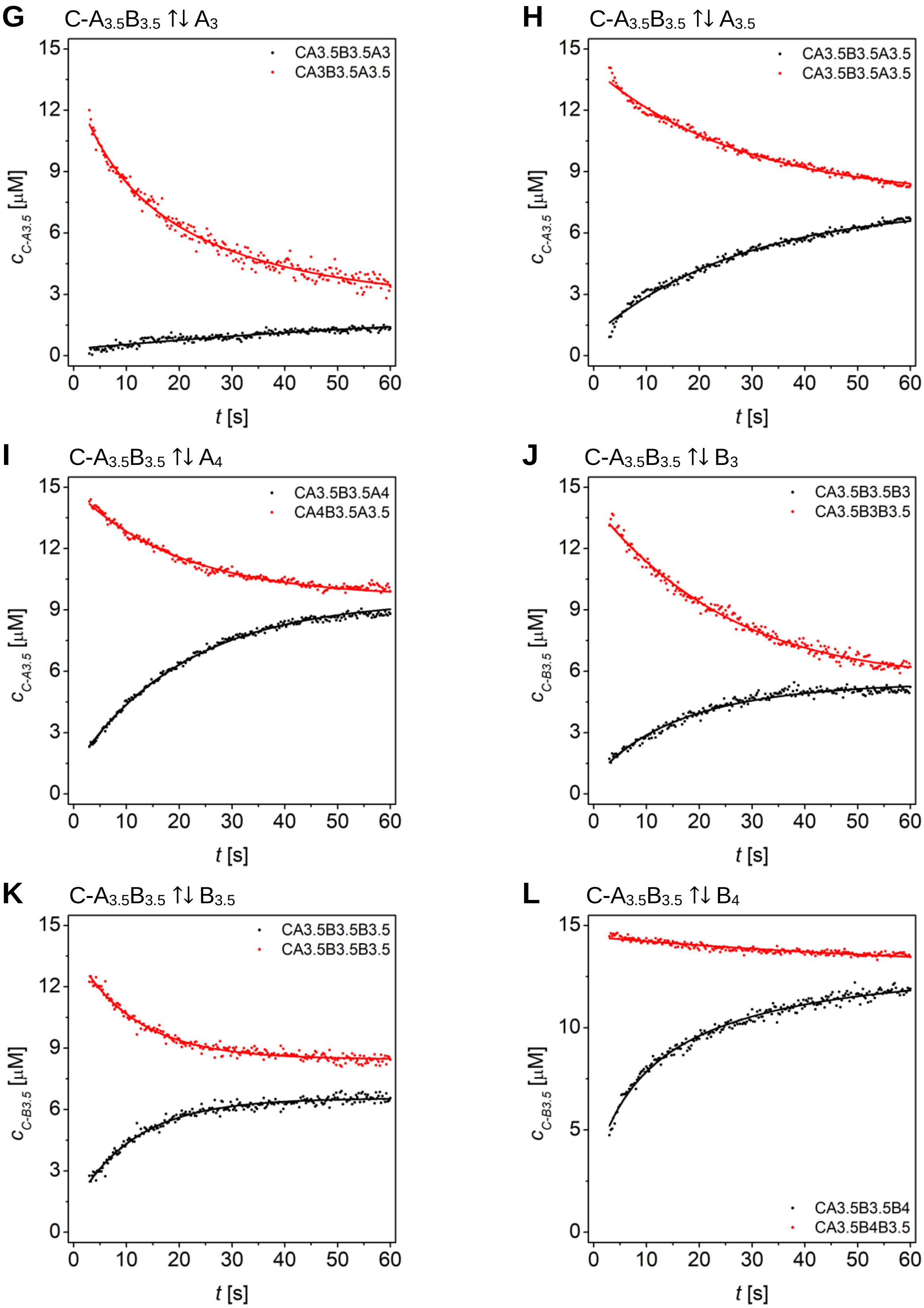
G
C-A3.5B3.5 ⇅ A3
CA3.5B3.5A3
CA3B3.5A3.5
c C-A3.5 [µM]
t [s]
H
C-A3.5B3.5 ⇅ A3.5
CA3.5B3.5A3.5
CA3.5B3.5A3.5
c C-A3.5 [µM]
t [s]
I
C-A3.5B3.5 ⇅ A4
CA3.5B3.5A4
CA4B3.5A3.5
c C-A3.5 [µM]
t [s]
J
C-A3.5B3.5 ⇅ B3
CA3.5B3.5B3
CA3.5B3B3.5
c C-B3.5 [µM]
t [s]
K
C-A3.5B3.5 ⇅ B3.5
CA3.5B3.5B3.5
CA3.5B3.5B3.5
c C-B3.5 [µM]
t [s]
L
C-A3.5B3.5 ⇅ B4
CA3.5B3.5B4
CA3.5B4B3.5
c C-B3.5 [µM]
t [s]

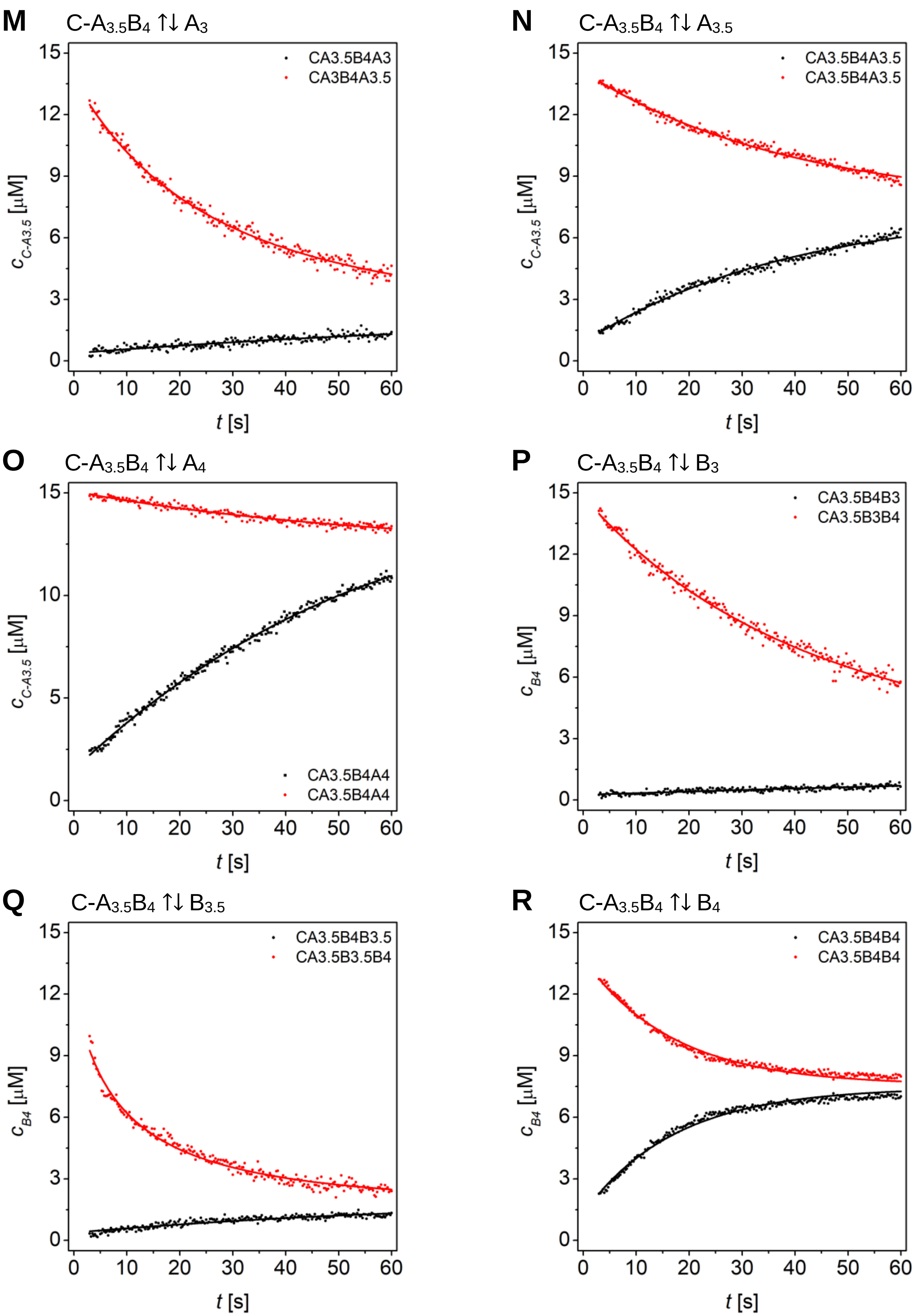

Figure S12. Dynafit results of least-squares fits strand displacement in C-$A_{3.5}$B peptides using a competitive binding model.

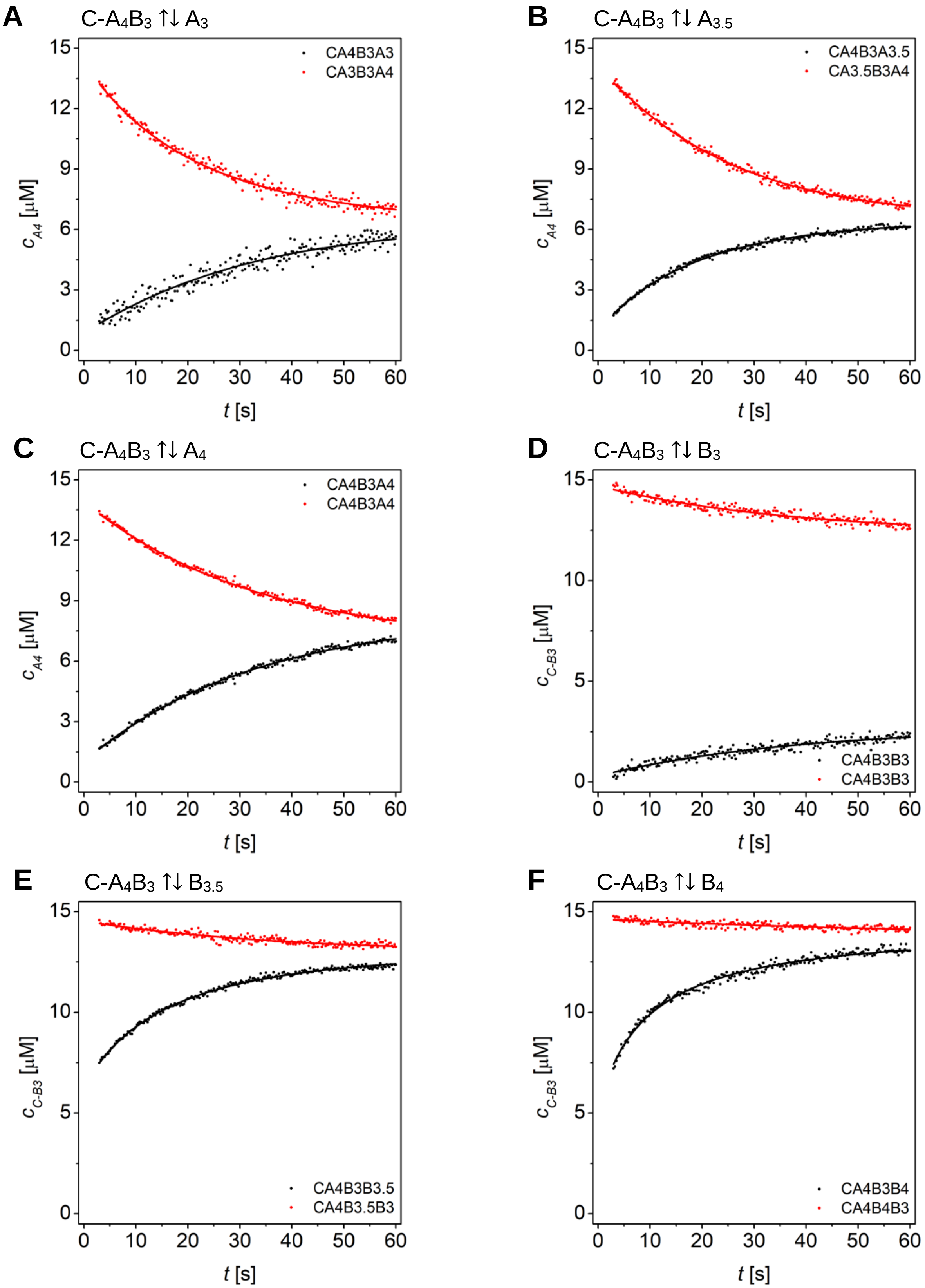
A
C-A4B3 ⇅ A3
CA4B3A3
CA3B3A4
B
C-A4B3 ⇅ A3.5
CA4B3A3.5
CA3.5B3A4
C
C-A4B3 ⇅ A4
CA4B3A4
CA4B3A4
D
C-A4B3 ⇅ B3
CA4B3B3
CA4B3B3
E
C-A4B3 ⇅ B3.5
CA4B3B3.5
CA4B3.5B3
F
C-A4B3 ⇅ B4
CA4B3B4
CA4B4B3
c_A4 [μM]
c_C-B3 [μM]
t [s]

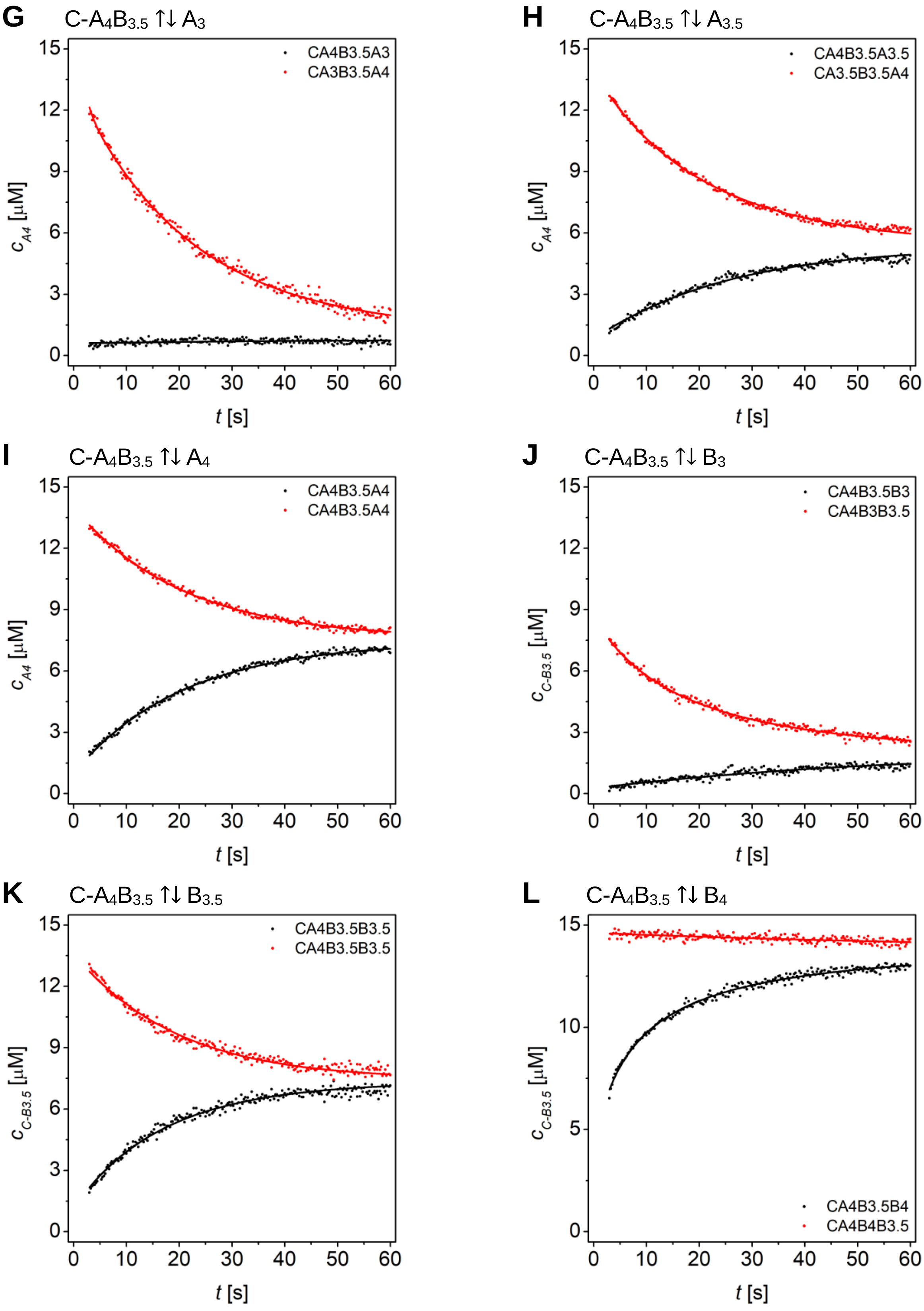
G
C-A4B3.5 ⇅ A3
CA4B3.5A3
CA3B3.5A4
H
C-A4B3.5 ⇅ A3.5
CA4B3.5A3.5
CA3.5B3.5A4
I
C-A4B3.5 ⇅ A4
CA4B3.5A4
CA4B3.5A4
J
C-A4B3.5 ⇅ B3
CA4B3.5B3
CA4B3B3.5
K
C-A4B3.5 ⇅ B3.5
CA4B3.5B3.5
CA4B3.5B3.5
L
C-A4B3.5 ⇅ B4
CA4B3.5B4
CA4B4B3.5
c_A4 [μM]
c_C-B3.5 [μM]
t [s]

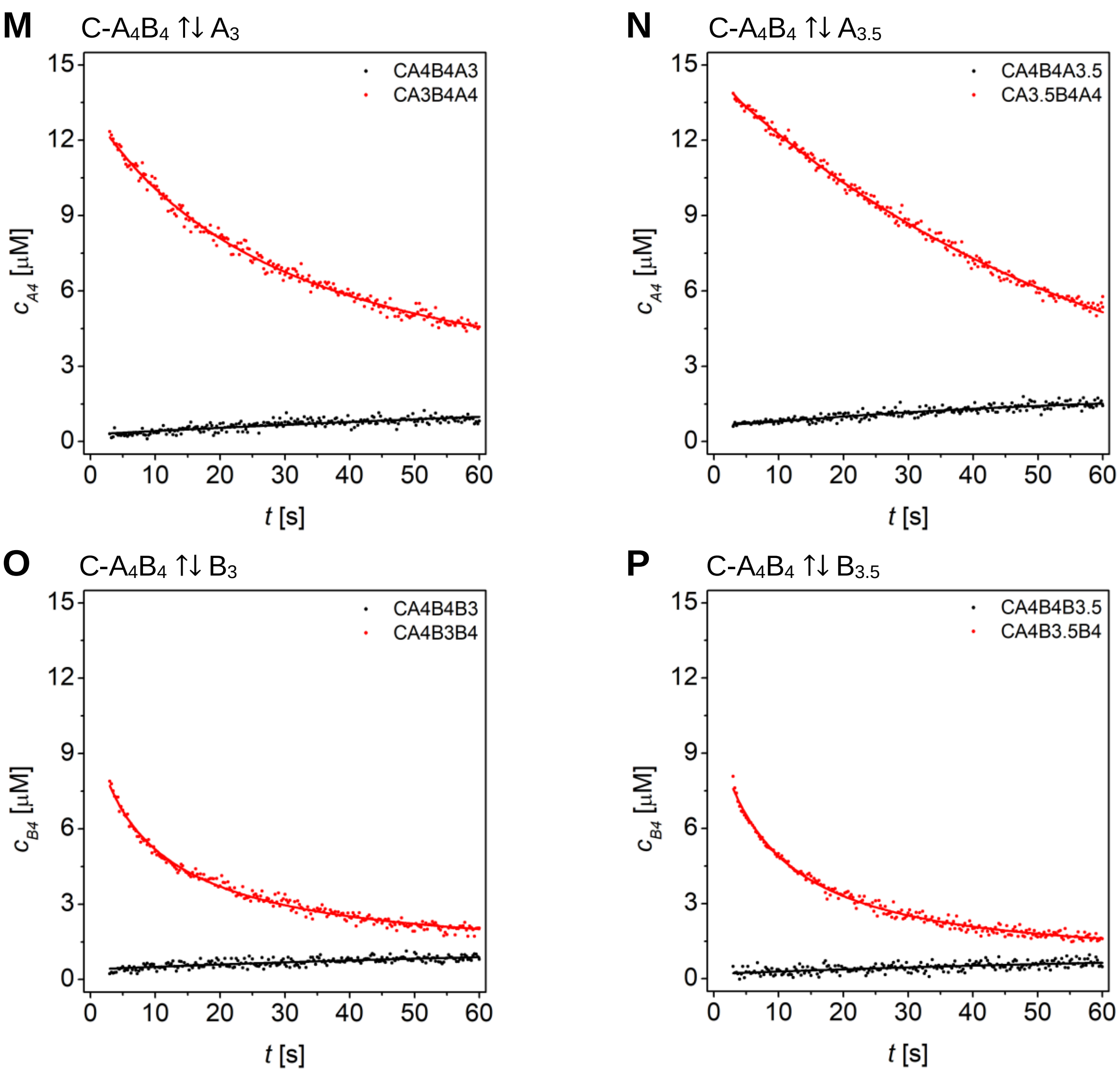

Figure S13 Dynafit results of least-squares fits strand displacement in C-A_4B peptides using a competitive binding model.

5. MALDI-TOF mass spectrometry and HPLC

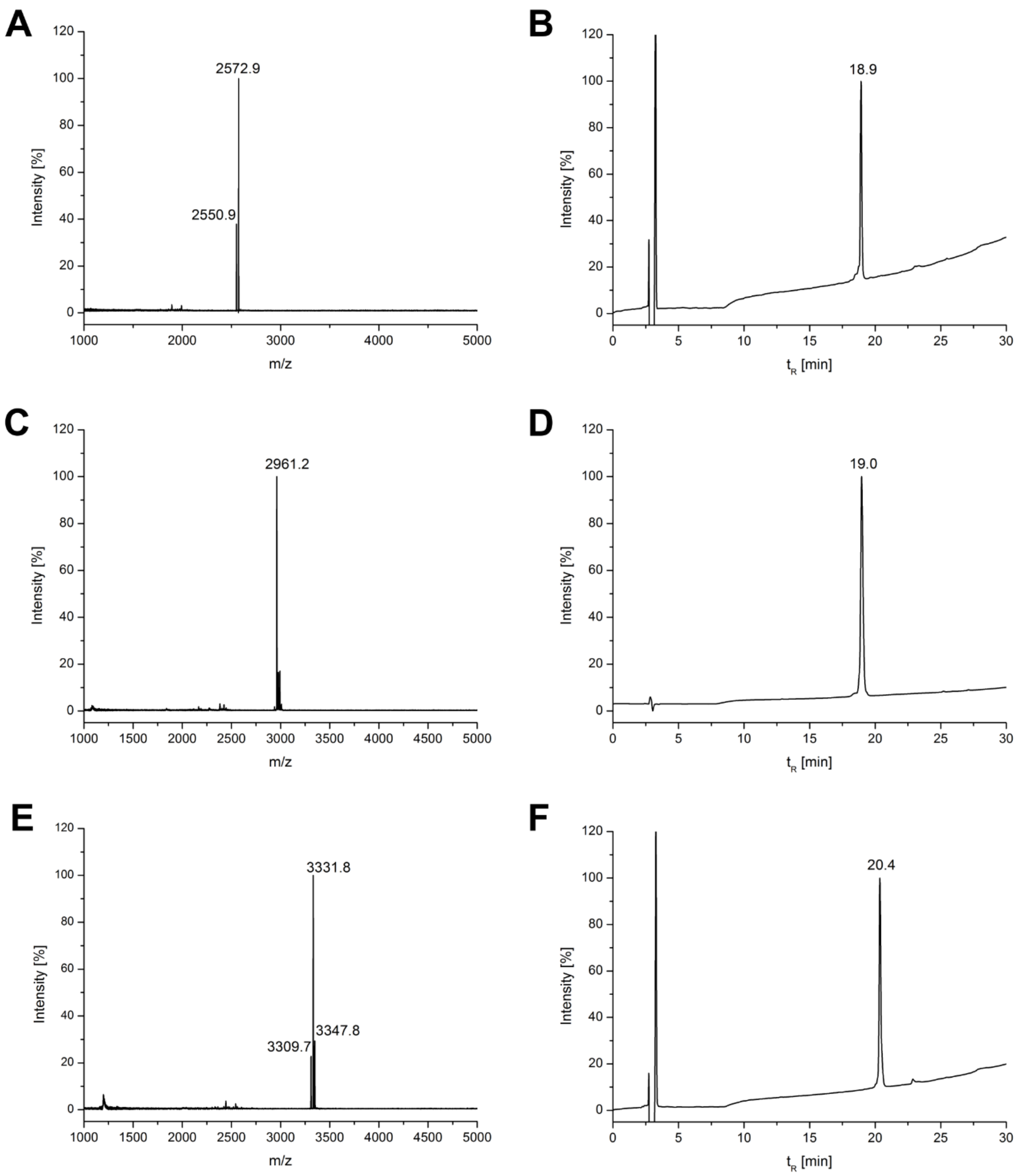

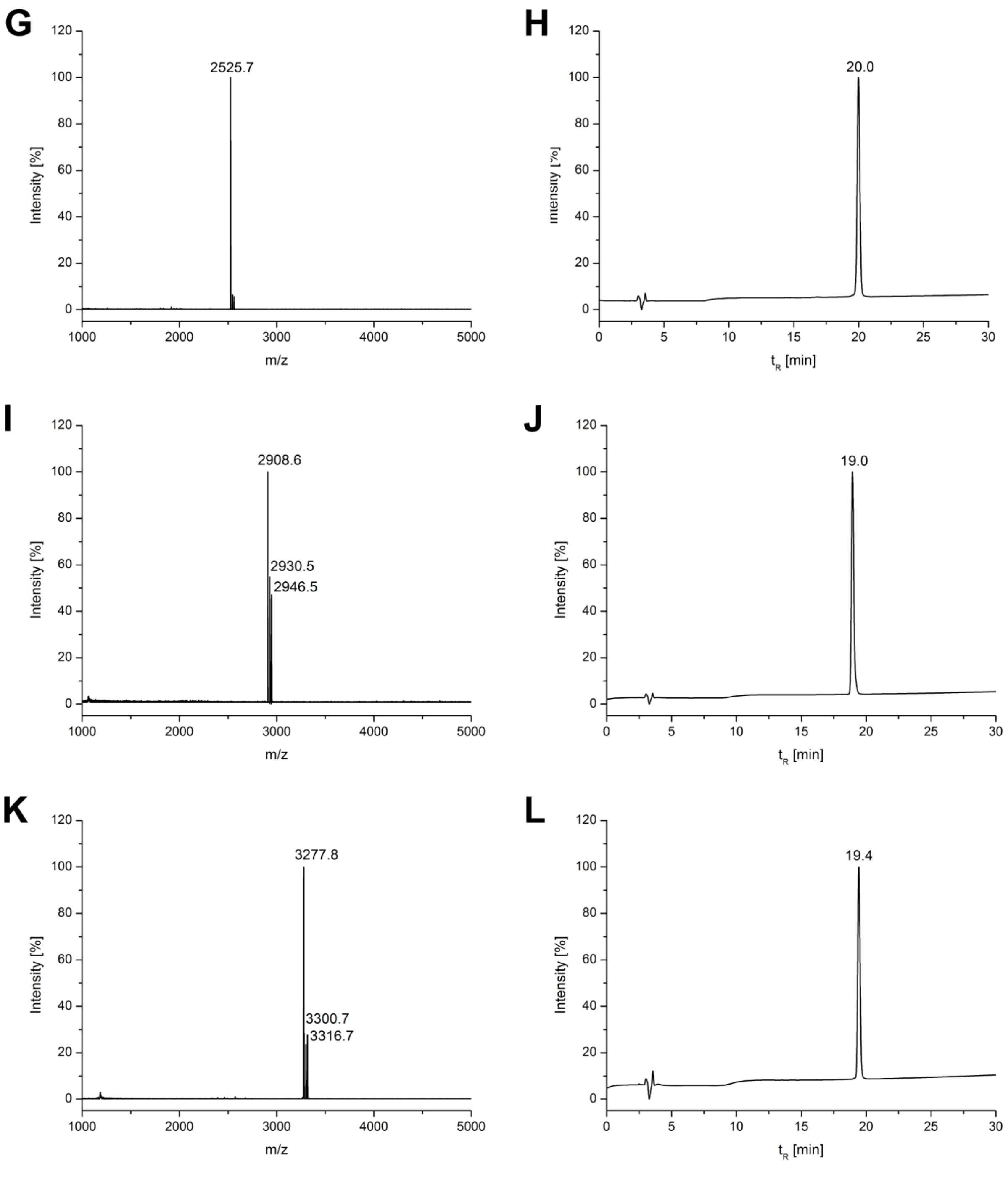

Figure S14 MALDI/TOF mass spectra and HPLC traces of coiled-coil single strands of the *C*-truncated set of heterodimeric coiled coils (A-B: C-A_3 calc. [M+H$^+$]: 2552.2, C-D: C-$A_{3.5}$ calc. [M+Na$^+$] 2961.2, E-F: A_4 calc. [M+H$^+$]: 3309.6, G-H: C-B_3 calc. [M+H$^+$]: 2525.1, I-J: C-$B_{3.5}$ calc. [M+H$^+$]: 2909.6, K-L: B_4 calc. [M+H$^+$]: 3279.0).

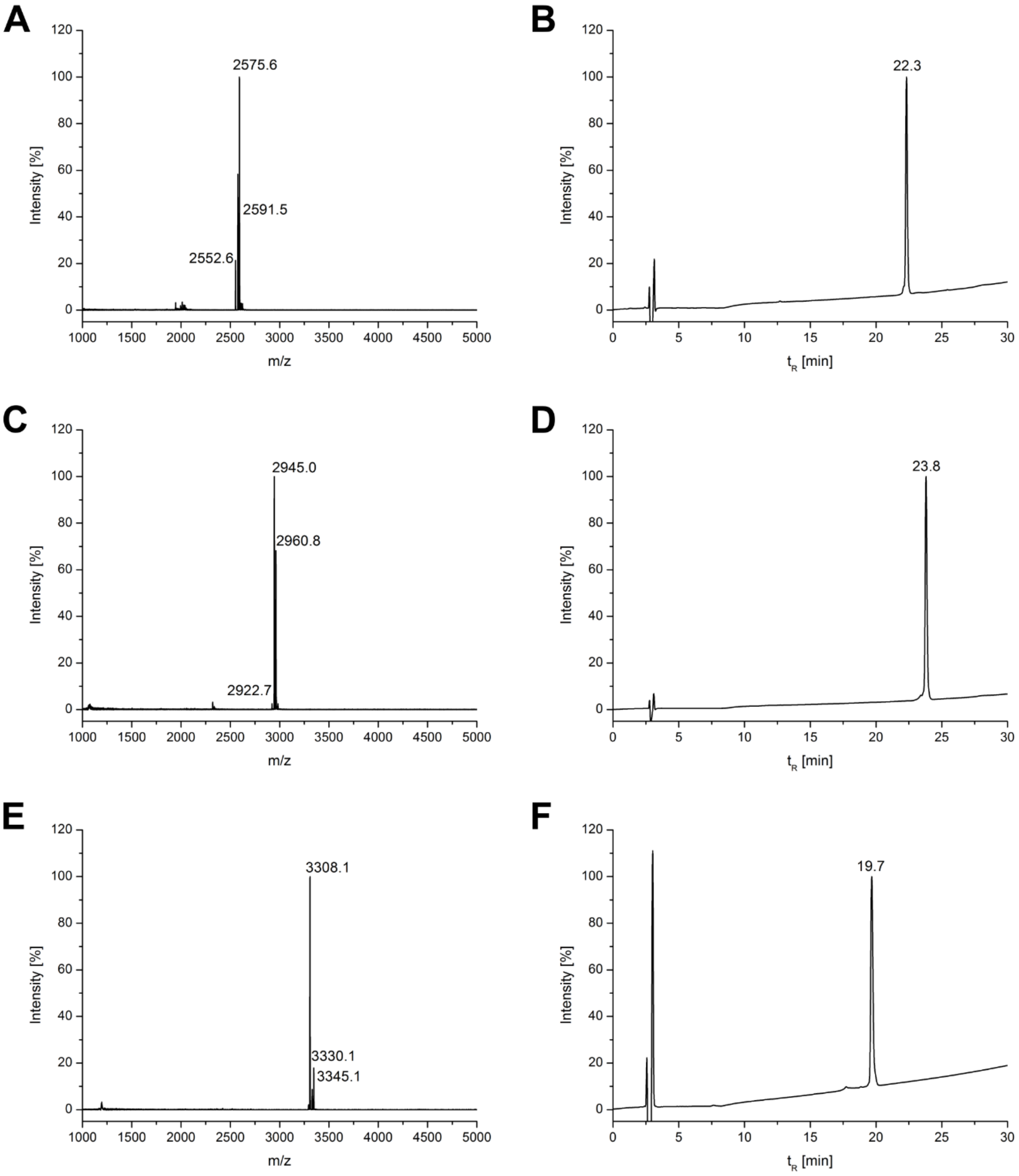
A
2575.6
2591.5
2552.6
Intensity [%]
m/z
B
22.3
Intensity [%]
t_R [min]
C
2945.0
2960.8
2922.7
Intensity [%]
m/z
D
23.8
Intensity [%]
t_R [min]
E
3308.1
3330.1
3345.1
Intensity [%]
m/z
F
19.7
Intensity [%]
t_R [min]

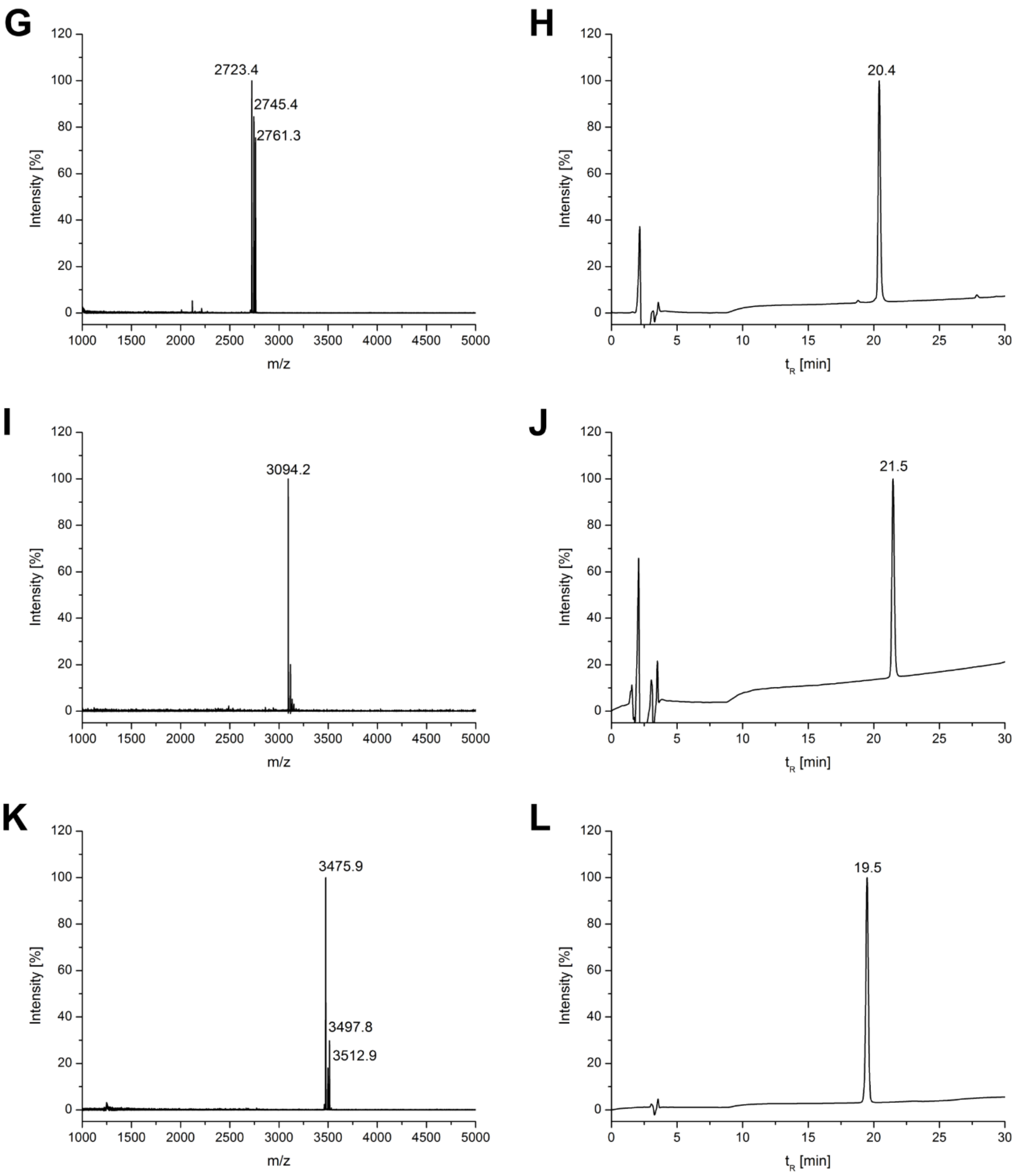

Figure S15. MALDI/TOF mass spectra and HPLC traces of coiled-coil single strands of the N-truncated set of heterodimeric coiled coils (A-B: N-A_3^* calc. [M+H$^+$]: 2553.3, C-D: N-$A_{3.5}^*$ calc. [M+Na$^+$]: 2945.5, E-F: A_4^* calc. [M+H$^+$]: 3309.6, G-H: N-B_3^* calc. [M+H$^+$]: 2723.3, I-J: N-$B_{3.5}^*$ calc. [M+H$^+$]: 3093.8, K-L: B_4^* calc. [M+H$^+$]: 3476.3).

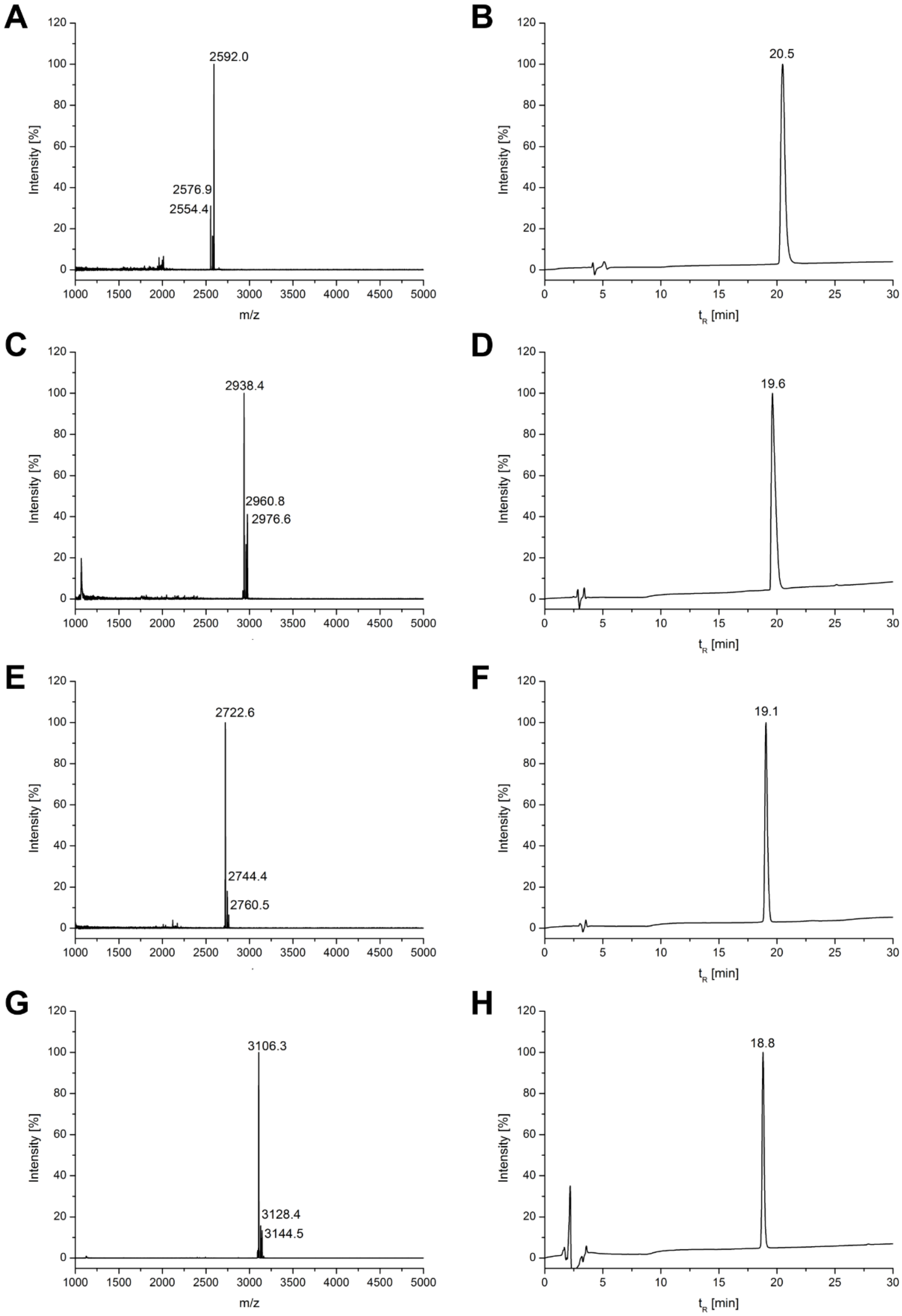

Figure S16. MALDI/TOF mass spectra and HPLC traces of coiled-coil single strands of the *C*-truncated set of heterodimeric coiled coils (A-B: C-A_3^* calc. [M+H$^+$]: 2553.2, C-D: C-$A_{3.5}^*$ calc. [M+H$^+$]: 3937.4, E-F: C-B_3^* calc. [M+H$^+$]: 2723.3, G-H: C-$B_{3.5}^*$ calc. [M+H$^+$]: 3106.9).

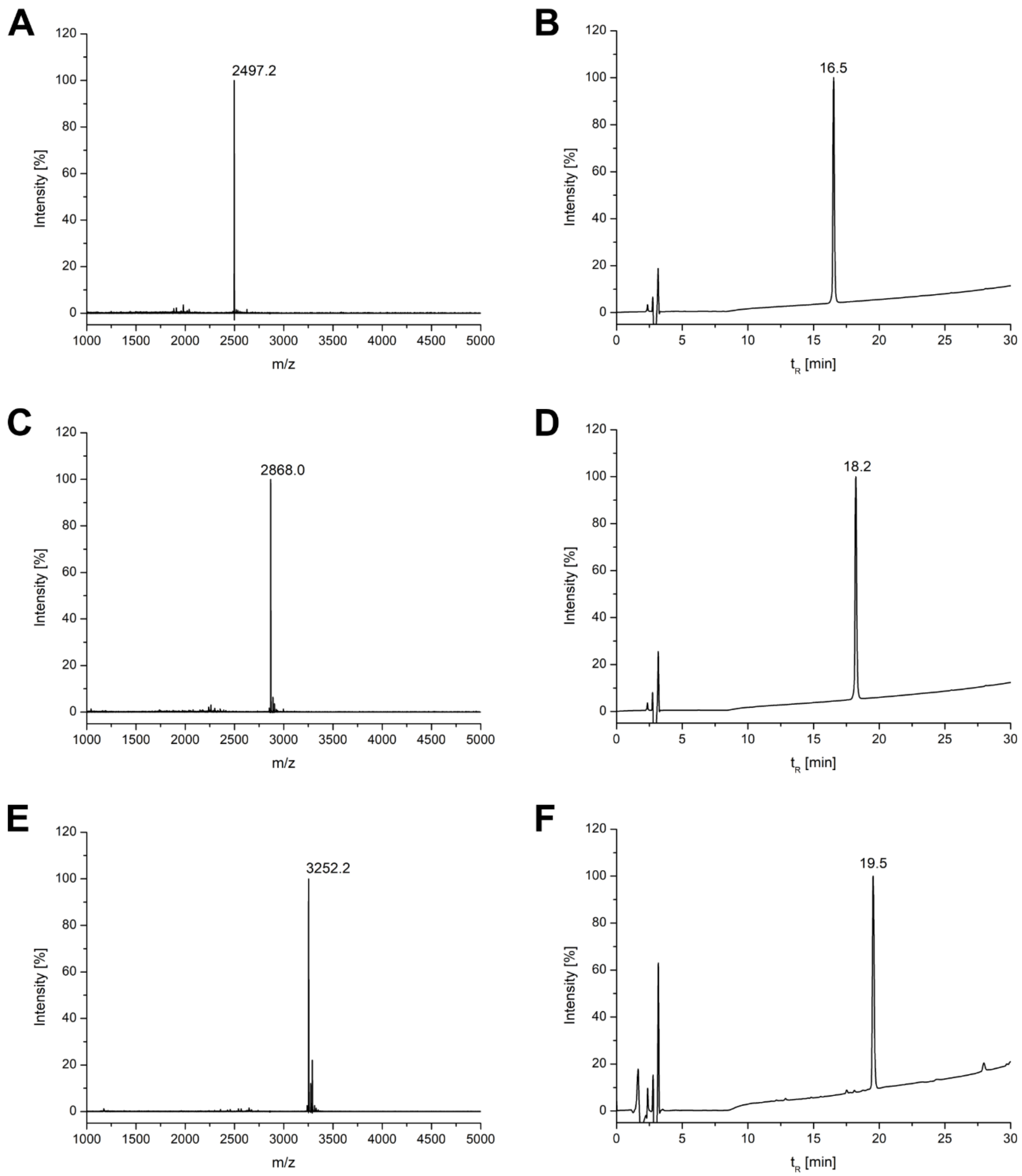

Figure S17. MALDI/TOF mass spectra and HPLC traces of non-labelled A-peptides of the *N*-truncated set of heterodimeric coiled coils (A-B: N-A3_W15K calc. [M+H$^+$]: 2496.7, C-D: N-A3.5_W18K calc. [M+H$^+$]: 2867.1, E-F: A4_W22K calc. [M+H$^+$]: 3251.6).

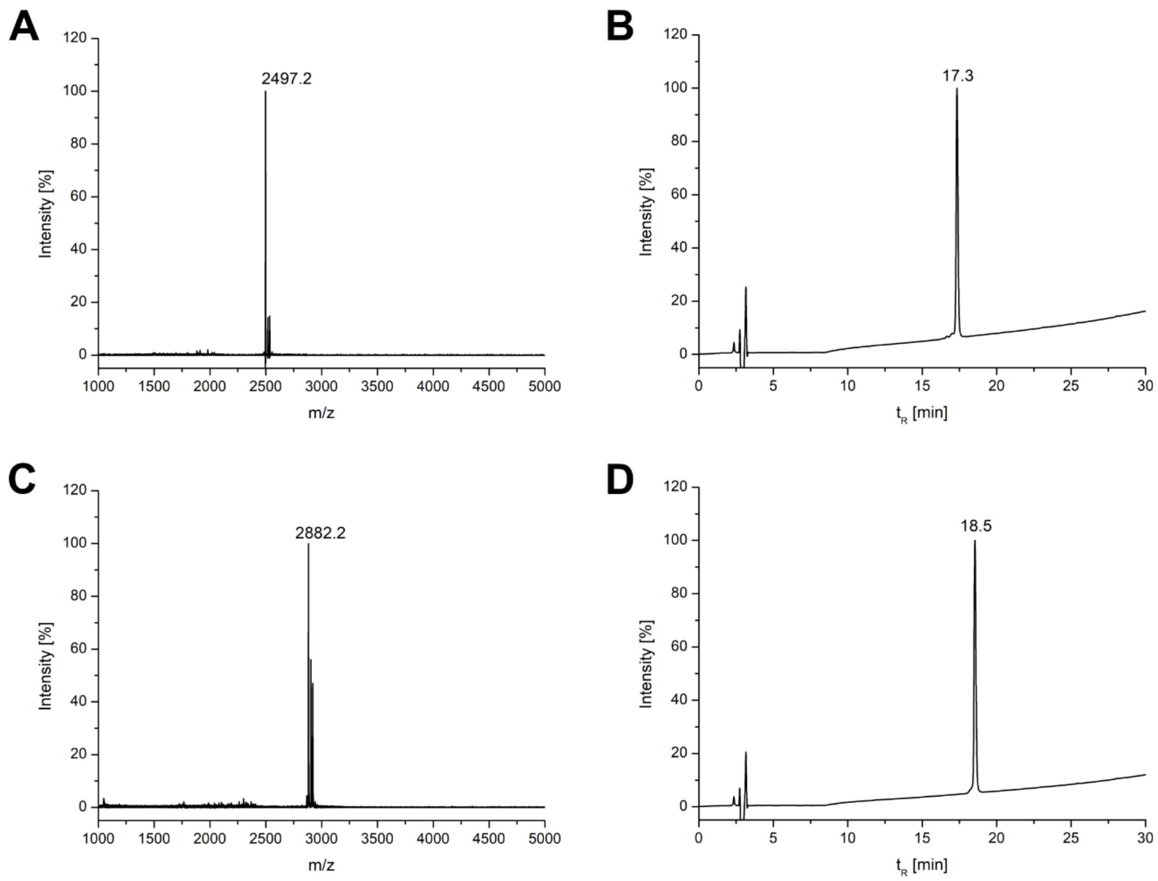

Figure S18. MALDI/TOF mass spectra and HPLC traces of coiled-coil single strands of the *C*-truncated set of heterodimeric coiled coils (A-B: C-A_3_W22K calc. [M+H$^+$]: 2496.7, C-D: C-$A_{3.5}$_W22K calc. [M+H$^+$]: 2881.2).

6. CD spectroscopy and thermal denaturation of *C*-truncated heterodimeric coiled coils

A C-A_3

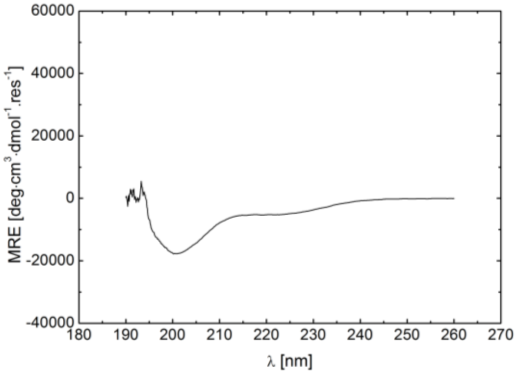

B C-A_3 CD melt

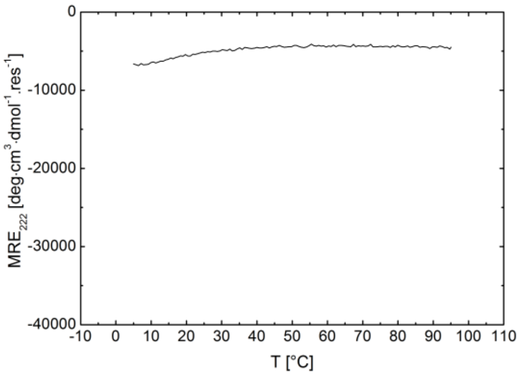

C C-$A_{3.5}$

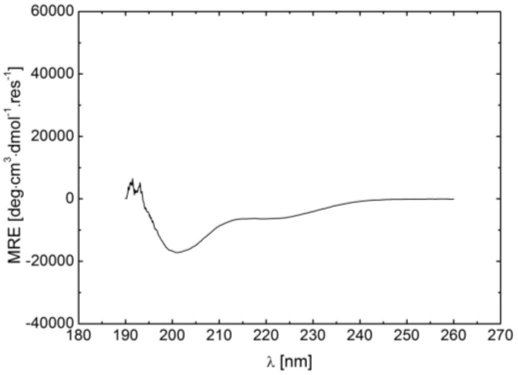

D C-$A_{3.5}$ CD melt

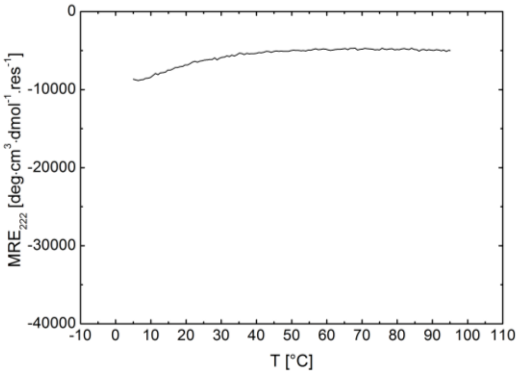

E A_4

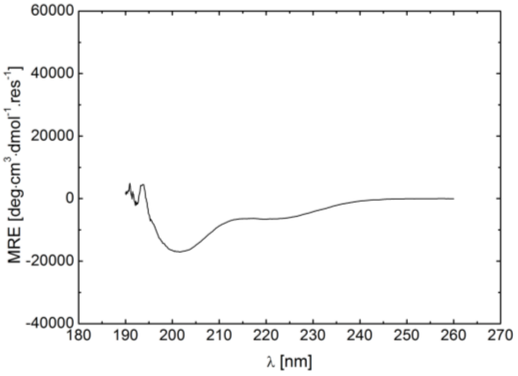

F A_4 CD melt

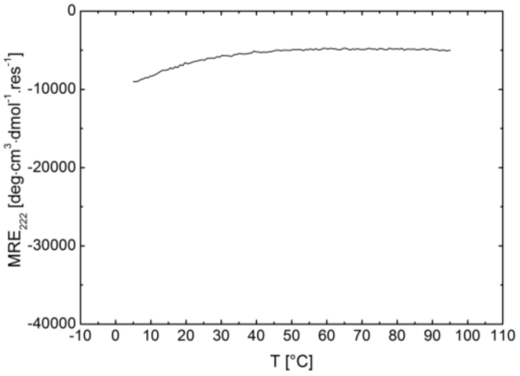

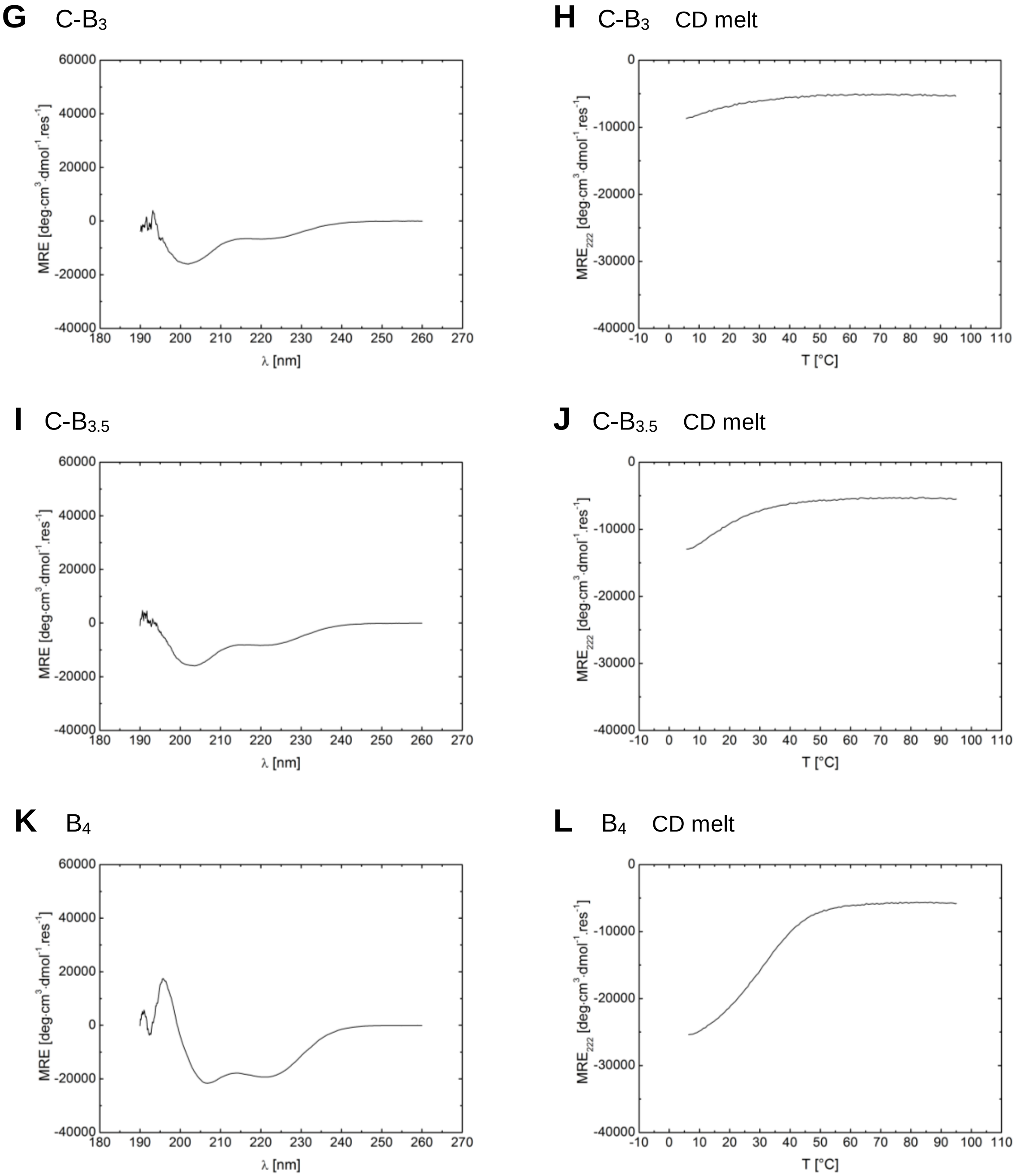

Figure S19. CD spectra (left) and thermal denaturation curves (right) of the coiled-coil single strands of the *C*-terminal truncated set of parallel heterodimeric coiled coils.

A C-A_3B_3

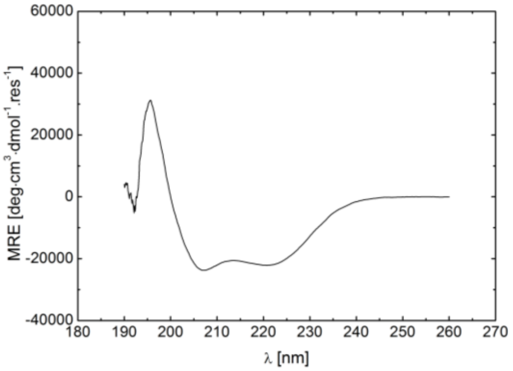

B C-A_3B_3 CD melt

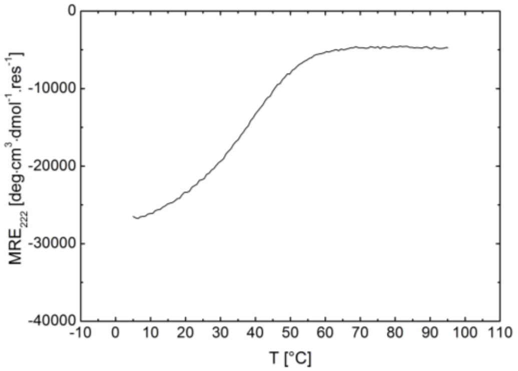

C C-$A_3B_{3.5}$

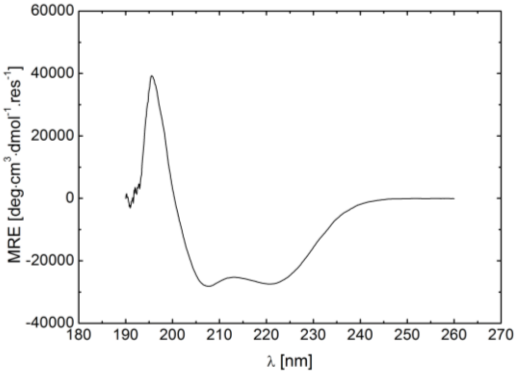

D C-$A_3B_{3.5}$ CD melt

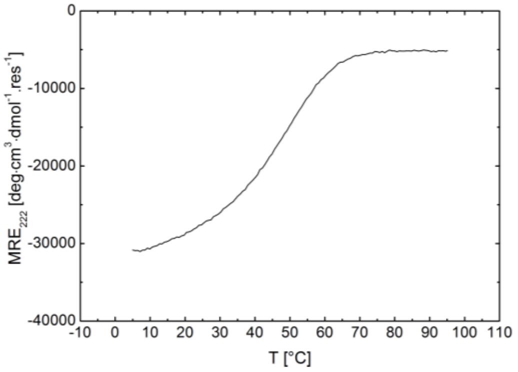

E C-A_3B_4

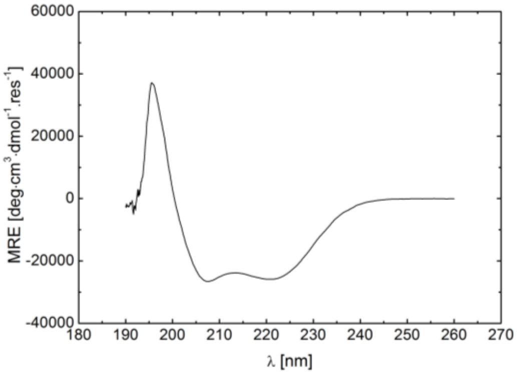

F C-A_3B_4 CD melt

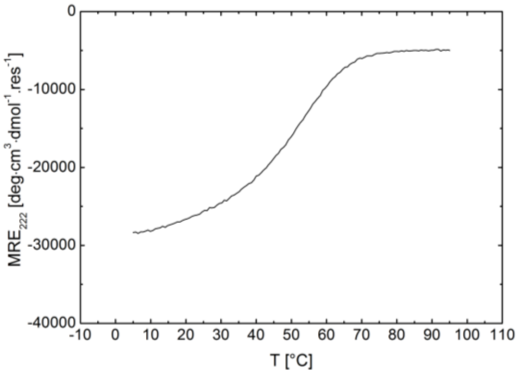

E C-$A_{3.5}B_3$

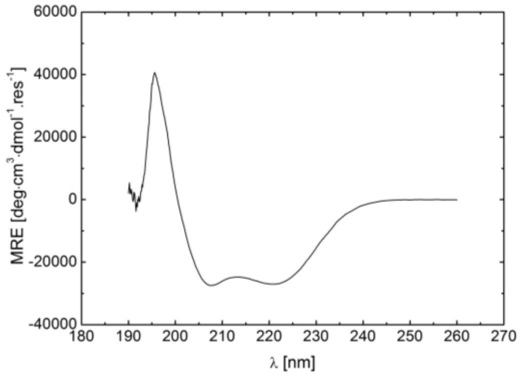

F C-$A_{3.5}B_3$ CD melt

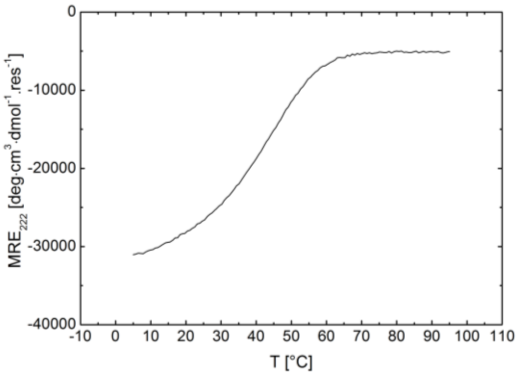

G C-$A_{3.5}B_{3.5}$

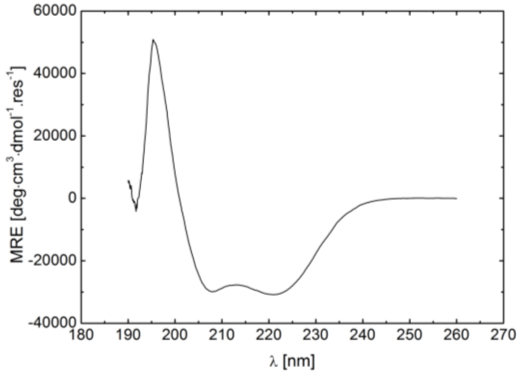

H C-$A_{3.5}B_{3.5}$ CD melt

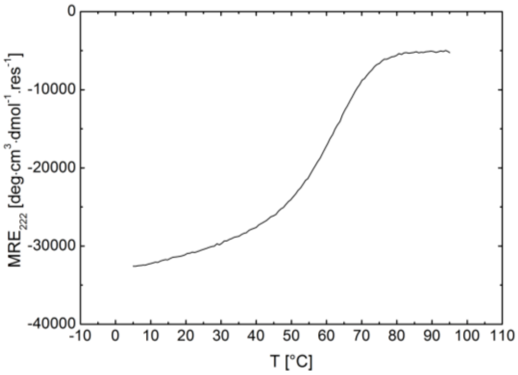

I C-$A_{3.5}B_4$

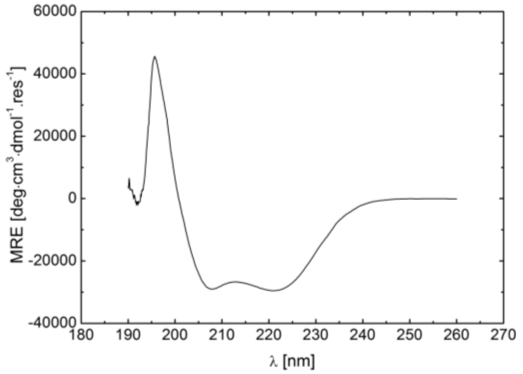

J C-$A_{3.5}B_4$ CD melt

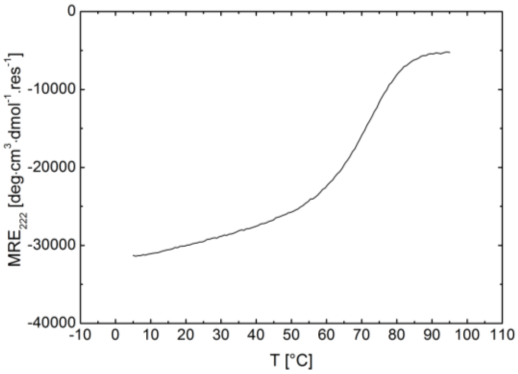

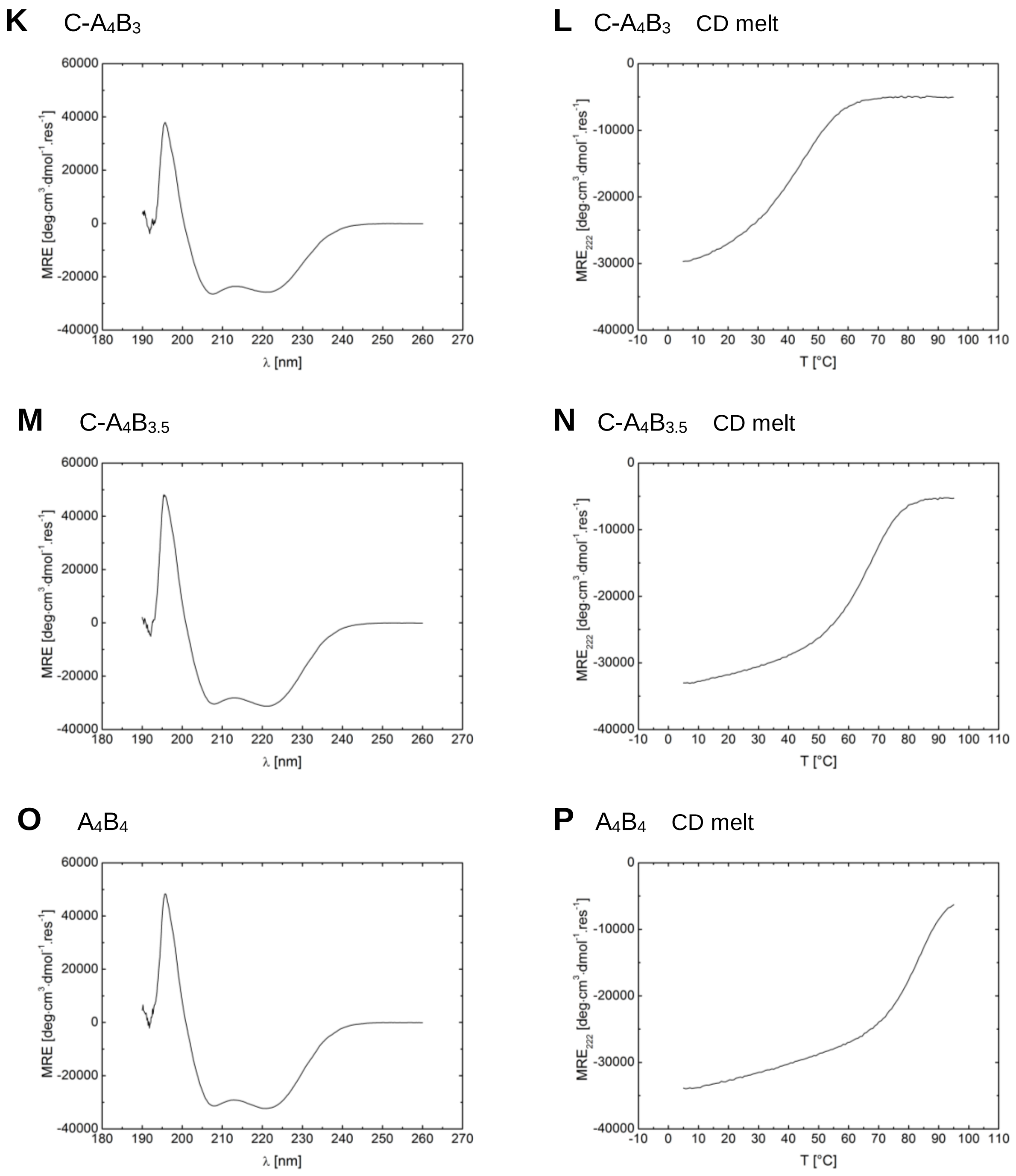

Figure S20. CD spectra (left) and thermal denaturation curves (right) of all possible combinations of *C*-terminal truncated set of parallel heterodimeric coiled coils.

7. References

1 M. Rabe, A. Boyle, H. R. Zope, F. Versluis and A. Kros, *Biopolymers*, 2015, **104**, 65–72.

2 P. Kuzmic, *Anal. Biochem.*, 1996, **237**, 260–273.

3 P. Kuzmic, *Anal. Biochem.*, 1999, **267**, 17–23.
